# Reactive oxygen species (ROS) in cancer: from mechanism to therapeutic implications

**DOI:** 10.1038/s41392-026-02583-x

**Published:** 2026-03-18

**Authors:** Sharmin Akter, Rajesh Madhuvilakku, Anik Kumar Kar, Irin Sultana Nila, Pengda Liu, Hiroyuki Inuzuka, Wenyi Wei, Yonggeun Hong

**Affiliations:** 1 Department of Biology, Indiana State University, Terre Haute, IN USA; 2 Biohealth Products Research Center (BPRC), Inje University, Gimhae, South Korea; 3 Inje DREAM Core Research Center, Inje University, Gimhae, South Korea; 4 Department of Digital Anti-aging Healthcare, Graduate School of Inje University, Gimhae, South Korea; 5 Department of Rehabilitation Science, Graduate School of Inje University, Gimhae, South Korea; 6 Department of Biochemistry and Biophysics, The University of North Carolina at Chapel Hill, Chapel Hill, NC USA; 7 Department of Pathology, Beth Israel Deaconess Medical Center, Harvard Medical School, Boston, MA USA

**Keywords:** Drug development, Drug development

## Abstract

Reactive oxygen species (ROS) act as critical secondary messengers in various intracellular signaling pathways that regulate cellular proliferation, differentiation, and survival under normal physiological conditions. However, dysregulation of redox signaling—driven by genetic mutations, epigenetic alterations, and posttranscriptional or posttranslational modifications—plays a central role in malignant transformation and cancer progression. Cancer cells typically exhibit elevated basal ROS levels due to increased metabolic activity, mitochondrial dysfunction, and oncogene activation. This moderate oxidative stress promotes tumorigenesis by inducing DNA damage, genomic instability, and aberrant activation of proliferative and survival pathways, while also contributing to resistance to conventional therapies. Paradoxically, excessive ROS accumulation can overwhelm antioxidant defenses, triggering oxidative stress-induced programmed cell death (PCD) mechanisms, including apoptosis, autophagy, and ferroptosis. Owing to its dual role—facilitating both tumor progression and suppression—ROS have emerged as compelling yet complex targets in cancer therapy. Therapeutic strategies aimed at modulating ROS homeostasis, such as enhancing ROS production, inhibiting antioxidant systems, or targeting downstream redox-regulated signaling nodes, hold promise for selectively eliminating cancer cells. Furthermore, integrating redox profiling or “redox signatures” into personalized medicine approaches may optimize therapeutic efficacy while minimizing off-target toxicity. In this review, we critically examine the Janus-faced role of ROS in carcinogenesis, dissect the molecular pathways regulated by ROS in tumor biology, and explore current advancements, limitations, and future directions in redox-based anticancer therapeutic approaches.

## Introduction

Reactive oxygen species (ROS) are an array of byproducts produced in cells through aerobic cellular metabolism that play a stimulatory role in multiple crucial signaling pathways in cells via alterations in intra- and extracellular environmental conditions.^1^ The ROS include nitric oxide (NO•), hydrogen peroxide (H_2_O_2_), superoxide anion (O_2_•^−^), hydroxyl radical (OH•), and organic peroxides that act as secondary messengers in many signaling mechanisms and are responsible for cell proliferation and differentiation.^1^ An imbalance between ROS production and antioxidant scavenging disrupts redox homeostasis, contributing to cancer onset and progression by causing genetic mutations, DNA damage, genomic instability, and altered cellular metabolism.^2^ Elevated ROS levels result in overactivation of key signaling pathways such as extracellular-regulated kinase 1/2 (ERK1/2), mitogen-activated protein kinase (MAPK), and phosphatidyl inositol 3 kinases (PI3Ks), which are crucial for regulating cell survival and propagation.^3^

On the other hand, an excessive amount of ROS may promote cellular damage, leading to cell death by regulating certain signaling cascades.^4^ This cell death signaling process involves the activation of different signaling cascades such as apoptosis signal-regulating kinase 1 (ASK1), poly (ADP-ribose) polymerase (PARP), and autophagy-related (ATG) 4 (ATG4) by ROS.^5^ Thus, the two-edged trait of ROS allows for the exertion of survival or death of cancer cells based on the intracellular levels of ROS.^6^

Manipulating ROS levels represents a promising therapeutic strategy involving the use of antioxidants, pro-oxidants, targeted ROS signaling inhibitors, combination therapies, and personalized approaches guided by ROS signatures.^7^ However, challenges remain, including achieving selectivity, determining optimal ROS thresholds, distinguishing ROS levels in healthy versus cancer cells, and bridging preclinical and clinical studies. Therefore, as an emerging research frontier, advancing redox-based cancer therapies requires a deep understanding of how aberrant redox states modulate signaling pathways that can both promote and inhibit tumor growth.

While many studies have focused on the mechanisms of ROS in cancer pathogenesis and ROS-modulating therapies,^6,7^ this review stands out by comprehensively examining the complex roles of ROS in cancer biology, exploring diverse signaling pathways and how ROS modulate them in cell survival and cell death, and offering detailed insights into therapeutic interventions guided by ROS pathogenesis and addressing challenges in cancer treatment.

## Insights into ROS

ROS are a collective term for a group of molecules derived from molecular oxygen, which are formed through redox reactions or electronic excitation that play a role in cellular signaling; however, when produced imbalanced, they can cause oxidative stress that damages cellular structures like lipids, proteins, and DNA.^7^ They can be classified into nonradical and free-radical species, with the latter having at least one unpaired electron.^8^ Among nonradical ROS, H_2_O_2_ is produced mainly by NADPH oxidases (NOX) and other enzymes and functions as a two-electron oxidant in redox signaling, with selective reactivity toward certain protein cysteines.^9^ Organic hydroperoxides (ROOHs) result from both enzymatic and nonenzymatic lipid peroxidation, contributing to cell signaling and cell death via ferroptosis.^10^ Singlet oxygen (^1^O_2_) is an electronically excited form of O_2_ generated through photoexcitation or enzyme reactions, especially in light-exposed tissues.^11^ O_2_•^−^ is a prominent ROS that is dismutated into H_2_O_2_, participating in various redox reactions, notably influencing lipid peroxidation and nitric oxide interactions.^12^ OH•, recognized as the most reactive ROS, is generated through Fenton chemistry and plays a critical role in initiating lipid peroxidation.^12^ Peroxyl radicals (ROO•) are significant in propagating lipid peroxidation chain reactions, whereas alkoxyl radicals (RO•) act as intermediates in the degradation of lipids.^13^ Each of these radicals contributes to cellular damage and oxidative stress through their highly reactive nature and involvement in complex biochemical processes, but in cancer, the O_2_•^−^, H_2_O_2_, OH•, and ROO• play major roles.^14^ O_2_•^−^ is involved in the early stages of oxidative stress and can generate more reactive species, such as H_2_O_2_, which contributes to cancer by inducing genetic mutations and chronic inflammation.^12^ OH• causes significant damage to DNA, lipids, and proteins, playing a major role in initiating lipid peroxidation and cancer development.^6^ H_2_O_2_ diffuses through membranes, disrupting signaling pathways, and promoting oxidative damage, with elevated H_2_O_2_ levels linked to increased cancer risk.^6^ ROO• propagates lipid peroxidation, leading to cellular damage and contributing to cancer.^13^

## ROS from a historical perspective

Oxygen is highly reactive and can form several types of ROS through both endogenous and exogenous factors. Among these, H_2_O_2_ was first discovered by Louis Jacques Thénard in 1818 in redox chemistry, although its role in biology was not fully understood until 1954.^15^

In 1900, catalase was recognized as a key enzyme in breaking down H_2_O_2_, marking an early discovery in antioxidant research.^16^ Selenium, identified as a toxic catalyst in 1817, was later found to be an essential component of the glutathione peroxidase (GPX) family, with GPX1 becoming widely accepted as a novel peroxidase in 1957.^17^ Thioredoxin reductase (TRXR), discovered in 1964, and peroxiredoxin (PRDX), discovered in 1968, were also noted as key components of antioxidant systems regulating cell responses to stress.^18^

That the enzyme NOX was linked to H_2_O_2_ production in phagocytes was disclosed in 1964,^17^ following earlier discoveries of superoxide radicals by Sbarra and Karnowski in 1959.^19^ Studies by Babior in 1973 confirmed O_2_•^−^ as the primary product of respiratory bursts, with H_2_O_2_ originating from its metabolism.^17^ McCord and Fridovich’s 1969 discovery of superoxide dismutase (SOD) showed that O_2_•^−^ could be converted to H_2_O_2_.^20^ The mitochondrial electron transport chain (ETC) was identified as a source of H_2_O_2_ in 1967, with Loschen in 1974 confirming that O_2_•^−^ is the precursor in mitochondria.^17^

Early studies on ROS highlighted their harmful effects, with key contributions from Gershman and Harman. In 1954, Gershman proposed that excessive oxidants cause tissue injury, linking oxidative stress to cellular damage.^21^ In 1956, Harman introduced the “free radical theory of aging,” suggesting that accumulated free radicals damage DNA, proteins, and lipids, driving aging and disease.^22^ In 1971, Loschen first demonstrated that ROS are generated during cellular respiration, a finding supported by Nohl and Hegner in 1978, emphasizing the role of ROS in biological processes.^23^ In 1977, Mittal and Murad reported that OH• activates guanylate cyclase, which leads to the production of cyclic guanosine monophosphate (GMP), a key signaling molecule.^24^ Halliwell and Gutteridge further refined the understanding of ROS in 1989, defining them as both free radicals and nonradical oxygen derivatives.^25^

As scientific understanding evolved in the late 20th century, research revealed that ROS, especially H_2_O_2_, are not only destructive agents but also essential regulators of cellular signaling.^17^ This shift in understanding demonstrated that ROS, particularly H_2_O_2_, play dual roles: as signaling molecules at low levels (1–100 nM) regulating growth, differentiation, and apoptosis and as damaging agents at higher levels (>100 nM), causing oxidative damage and cell death.^17^ The balance between ROS and antioxidant defense relies on adaptive pathways such as the nuclear factor erythroid 2–related factor 2 (Nrf2)/Keap1 and NF-κB pathways. Nrf2, which can induce the expression of phase II detoxifying enzyme-encoding genes through antioxidant response elements (AREs), was defined as a master regulator of antioxidant systems in 1997.^17^

Methionine was discovered in 1921 by John Howard Mueller,^26^ but its critical role in oxidative modification emerged decades later. In 1999, Van Patten et al. revealed the involvement of methionine in the oxidative modification of antithrombin by H_2_O_2_.^27^ Earlier, in 1981, Brot et al. identified methionine sulfoxide reductase A (MsrA) as the first enzyme to repair oxidized methionine.^28^ MsrB, which is specific for methionine-R-sulfoxide (Met-RO), was discovered in 1999 by Hans J. Forman’s team.^29^ In 2007, free methionine-R-sulfoxide reductase (fMRSR), which is capable of reducing free Met-RO but not protein-bound Met-RO, was identified.^30^ Finally, in 2013, Drazic and colleagues highlighted methionine oxidation as a novel mechanism for redox regulation of protein function.^31^

The role of ROS in cell death signaling emerged in the late 1990s. In 1998, David G. Green and colleagues identified ROS as key regulators of programmed cell death.^32^ In 2007, Scherz-Shouval et al. reported that ROS critically induce autophagy.^33^ In 2009, Cho et al. demonstrated that mitochondrial ROS are essential for necroptosis execution.^34^ In 2008, Marcus Conrad’s team reported that redox gene modulation could trigger nonapoptotic death, and Yang and Stockwell reported that this form was iron dependent.^35^ In 2012, Brent Stockwell’s group formally defined this distinct, iron-regulated, lipid peroxidation-driven cell death as “ferroptosis”.^36^

**Fig. 1:**
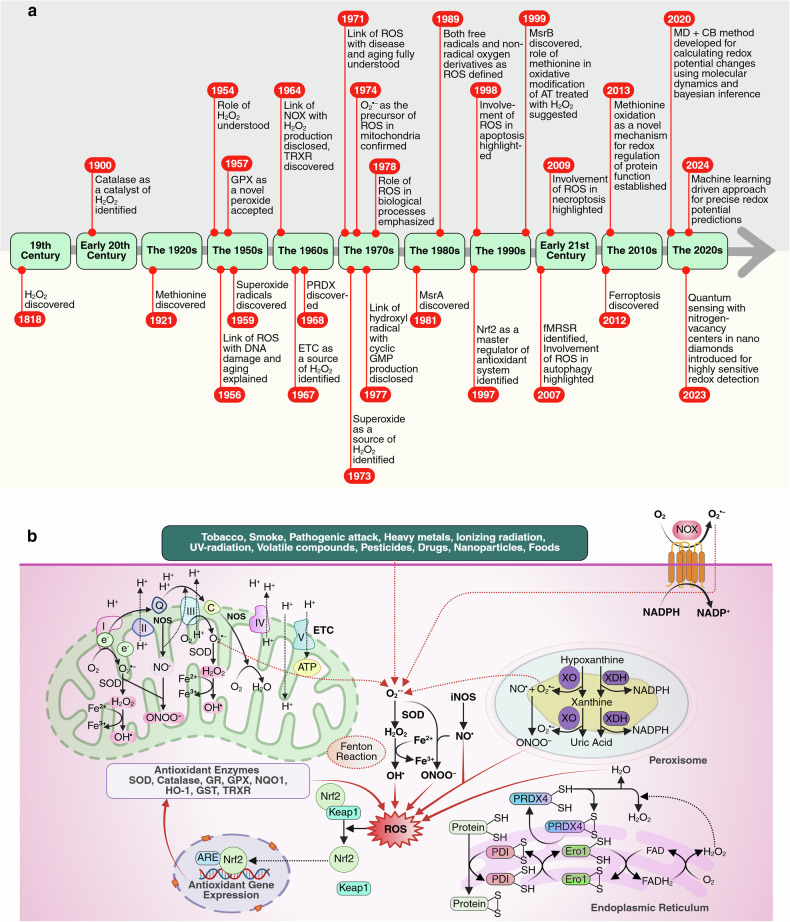
**a** Key events in the history of the ROS. **b** The generation of ROS, their regulation through antioxidant defenses, and the role of the Nrf2-Keap1 signaling pathway in oxidative stress. Mitochondria, peroxisomes, the ER, and NOX enzymes are major sources of intracellular ROS. In mitochondria, electron leaks at ETC complexes I and III produce superoxide (O_2_•^−^), which SOD converts to hydrogen peroxide (H_2_O_2_) for redox signaling or detoxification via catalase, glutathione, or thioredoxin systems. Peroxisomes generate ROS through xanthine oxidase (XO) and fatty acid metabolism, with elevated ROS linked to leukemia. ER ROS arise from oxidative protein folding via PDI and Ero1, with disruptions inducing ER stress and the UPR. External factors such as pollutants and radiation also increase ROS. Cells maintain redox homeostasis through antioxidants such as Nrf2-regulated enzymes (SOD, catalase, GPX), which neutralize ROS and prevent oxidative damage. The GSH/GSSG and TRX systems, fueled by NADPH, restore redox balance, emphasizing the interplay between ROS generation and antioxidant defenses in cellular health and disease. This figure was created in BioRender. tonu, r. (2025) (https://BioRender.com/0q9bi92). GPX glutathione peroxidase, NOX NADPH oxidase, TRXR thioredoxin reductase, ETC electron transport chain, PRDX perodoxine, GMP guanosine monophosphate, Msr methionine sulfoxide reductase, AT antithrombin, fMRSR free methionine-R-sulfoxide reductase, MD + CB molecular dynamics and Bayesian inference, CAT catalase, GST glutathione S-transferase, NQO1 NADPH quinone oxidoreductase 1, HO-1 heme oxygenase 1

Research on redox detection has progressed significantly since the early 20th century, evolving from basic electrochemical methods to advanced computational and machine learning (ML)-driven approaches. In 2020, Barton and colleagues developed a biocompatible nanoscale tool using quantum sensing with nitrogen-vacancy (NV) centers in nanodiamonds (NDs) coupled to nitroxide radicals, enabling highly sensitive detection of paramagnetic species (~10 spins per ND) and real-time monitoring of redox processes such as ascorbic acid oxidation.^37^ In 2023, Oliveira and colleagues developed a method (MD + CB) to calculate redox potential changes by integrating fluctuation relations with molecular dynamics (MD) simulations, efficiently estimating redox potentials using Bayesian inference.^38^ Tested on heme proteins, it showed reliable shifts (0.85 correlation) and matched experimental data, aiding in the understating on ROS biology by advancing the prediction and engineering of redox-active proteins and understanding redox signaling in cellular processes and diseases.^38^ In 2024, Jinnouchi and colleagues developed a method combining first-principles calculations and machine learning to predict redox potentials with high precision.^39^ The method accurately modeled Fe^3+^/Fe^2+^, Cu^2+^/Cu^+^, and Ag^2+^/Ag^+^ redox potentials, closely aligning with experimental values, advancing ROS biology by enhancing redox reaction modeling and electron transfer studies^39^ (Fig. 1a).

## Source and mechanism of ROS generation

ROS are generated from both endogenous (internal) and exogenous (external) sources. Endogenous sources include cellular processes like mitochondrial respiration and enzyme activity, whereas exogenous sources encompass environmental factors and lifestyle choices. These sources contribute to the overall cellular redox balance, and their dysregulation can lead to oxidative stress and cellular damage.

### Endogenous sources of ROS

Endogenous sources of ROS include various cellular organelles and enzymatic reactions within the body:

#### Mitochondria

Mitochondrial ROS generation primarily occurs at complexes I and III of the ETC due to electron leaks that fail to reach complex IV and instead univalently react with oxygen to form O_2_•^−^.^40^ Superoxide, a membrane-impermeable molecule, is rapidly dismutated into membrane-diffusible H_2_O_2_ by SOD.^41^ H_2_O_2_ is further detoxified into water by catalase, glutathione peroxidase, or thioredoxin peroxidase or participates in redox signal transduction. Alternatively, H_2_O_2_ can also undergo further reduction via the Fenton reaction to form OH•, a highly toxic molecule that causes oxidative damage.^41^ Additionally, mitochondrial nitric oxide synthase (NOS) produces nitric oxide (NO•), which then reacts with O_2_•^−^ to form peroxynitrite (ONOO^−^), a potent oxidant^42^ (Fig. 1b).

#### Peroxisome activity

Peroxisomes, the major sites of intracellular H_2_O_2_, represent another important source of ROS. In addition, xanthine oxidase (XO) and xanthine dehydrogenase (XDH), which are involved in purine metabolism, generate O_2_•⁻ while catalyzing the oxidation of hypoxanthine to xanthine and xanthine to uric acid (UA).^43^ XO also reduces nitrate and nitrite to NO•, which reacts with O_2_•⁻ to form ONOO⁻, a reactive nitrogen species (RNS). Additionally, an electron transport chain in the peroxisomal membrane contributes to O_2_•⁻ production. H_2_O_2_ is released during these catalytic activities and can form OH• via Fenton reactions^43^ (Fig. 1b).

#### Endoplasmic reticulum (ER)

The ER, an organelle critical for protein synthesis, folding, maturation, and assembly, is another significant source of ROS.^44^ Oxidative protein folding within the ER is facilitated by oxidoreductases such as protein disulfide isomerases (PDIs), ERp72, and ERp57, with PDI catalyzing thiol-disulfide exchange reactions to form native disulfide bonds in proteins.^45^ During this process, PDI is oxidized by endoplasmic reticulum oxidoreductin-1 (Ero1), which transfers electrons from reduced PDI to oxygen, producing H_2_O_2_. Ero1 also promotes the conversion of reduced glutathione (GSH) to glutathione disulfide (GSSG), contributing to ROS accumulation and ER stress.^46^ Disruptions in these pathways can lead to protein misfolding and accumulation, triggering ER stress and the unfolded protein response (UPR), thereby inducing cancer. While the UPR initially supports tumor cell survival and propagation, prolonged ER stress can ultimately induce apoptosis in tumor cells^44^ (Fig. 1b).

#### NOX

The NOX family of enzymes was the first system identified to produce ROS as a primary function rather than as a metabolic byproduct.^47^ Evidence strongly correlates NOX-driven ROS generation with leukemogenesis, disease progression, and drug resistance. In 2023, Germon et al. linked FLT3 mutations in AML to increased protein oxidation, phosphorylation, and elevated NOX2 complex activity.^48^ NOX2 inhibition synergistically enhances apoptosis in FLT3-mutant AML cells and reduces FLT3 phosphorylation and cysteine oxidation, identifying NOX2 as the primary source of ROS.^48^ This finding aligns with previous in vitro studies demonstrating significant reductions in intracellular ROS levels following NOX inhibition or genetic knockout of NOX isoforms or subunits^49^ (Fig. 1b).

Besides, thymidine phosphorylase (TP) enhances ROS production by increasing NADPH levels, which activate NADPH oxidase, and through metal-catalyzed oxidation of excess 2-deoxy-D-ribose-1-phosphate (DR1P).^50^ Polyamines (PAs), such as spermine and spermidine, produce H_2_O_2_ through catabolic processes involving enzymes like spermine oxidase (SMOX). Additionally, diamine oxidase (DAO) and acetylpolyamine oxidase (APAO) produce H_2_O_2_ during polyamine oxidation.^50^ The cytochrome P450 (CYP) enzymes in the endoplasmic reticulum and mitochondria, as well as the cyclooxygenases (COXs) and lipoxygenases (LOXs) involved in eicosanoid metabolism, contribute to ROS formation.^51^ The PI3K/AKT/phosphatase and TENsin homolog deleted on chromosome 10 (PTEN) signaling pathway influences NOX-derived ROS production.^52,53^ Enzymes such as protein kinase C (PKC), MAPK, and protein kinase A (PKA) modulate NOX activation, whereas the tumor suppressor p53 regulates both antioxidant and prooxidant responses, promoting ROS production and apoptosis in cancer cells.^52,53^

### Exogenous sources of ROS

Exogenous sources of ROS include external factors outside the body or environmental exposures:

External factors such as pollutants, tobacco smoke, radiation, and certain drugs induce ROS through various mechanisms.^54^ For example, tobacco smoke contains more than 4000 chemicals, such as superoxide and hydroxyl radicals. Ionizing radiation can also generate hydroxyl radicals, either directly by oxidizing water or indirectly through the formation of secondary ROS. Transition metals such as iron, copper, zinc, and aluminum catalyze the Fenton and Haber-Weiss reactions, leading to the formation of highly reactive OH• and hydroxyl anion (OH^−^) from H_2_O_2_.^55^ Carcinogenic metals like antimony, arsenic, and chromium similarly induce ROS through these reactions. Additionally, pathogenic invasion can trigger immune responses, leading to the activation of neutrophils and macrophages, which generate ROS as part of the host defense mechanism.^56^ Collectively, various intracellular and extracellular stimuli contribute to ROS formation in cancer cells (Fig. 1b).

## Redox homeostasis

**Fig. 2:**
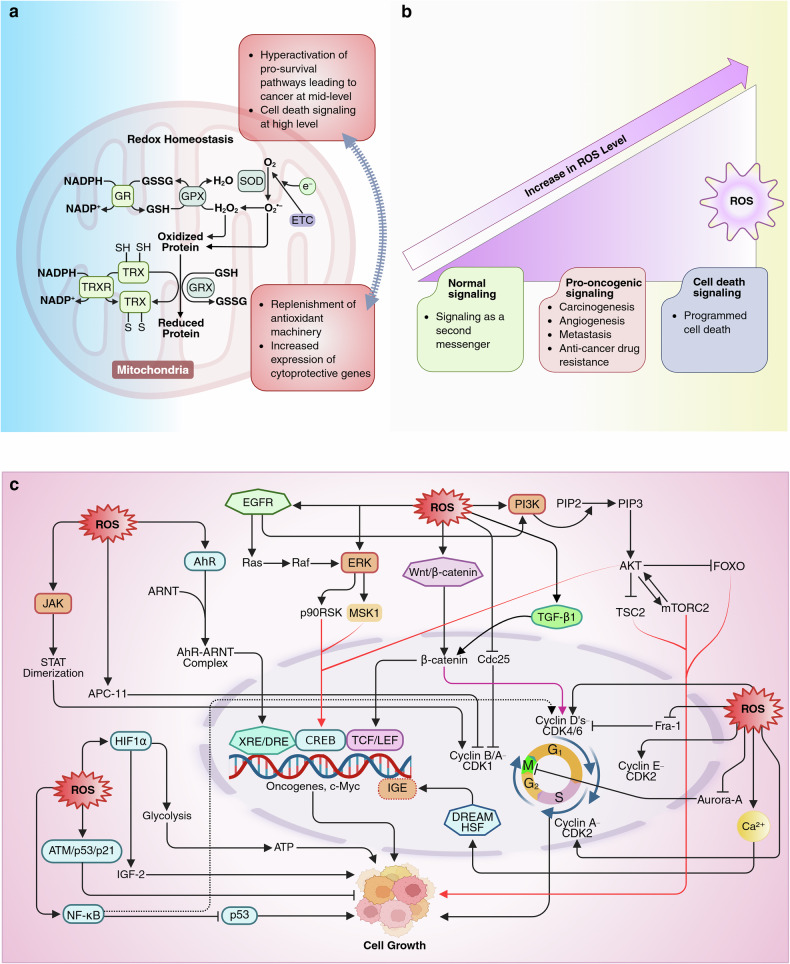
**a** Redox homeostasis, showcasing the balance between oxidative stress and the antioxidant defense system in cells. The diagram highlights key enzymatic pathways managing redox balance. Superoxide dismutase (SOD) converts O_2_•^−^ into H₂O₂, which is further detoxified into water by GPX via GSH. GSH is regenerated from its oxidized form (GSSG) by glutathione reductase (GR) via the use of NADPH as a reducing agent. Similarly, the TRX system, mediated by thioredoxin reductase (TRXR), converts oxidized thioredoxin to its reduced state to maintain protein redox states. Glutaredoxin (GRX) also participates in reducing oxidized proteins, ensuring functional protein repair, and maintaining cellular homeostasis. The figure outlines two outcomes depending on redox imbalance. On the one hand, excessive oxidative stress leads to hyperactivation of prosurvival pathways, increasing cancer risk, and at higher levels, triggers cell death signaling. On the other hand, a sufficient antioxidant response replenishes the antioxidant machinery and enhances the expression of cytoprotective genes. **b** The dual role of ROS: as mediators of normal signaling at low levels and triggers of both tumor progression and cell death depending on concentration. On the left, normal signaling is shown, where ROS act as second messengers in cellular signaling pathways, maintaining physiological processes. In the middle, at moderate ROS levels, pro-oncogenic signaling is highlighted, promoting carcinogenesis, angiogenesis, metastasis, and resistance to anticancer drugs. At the highest ROS levels, on the right, cell death signaling becomes dominant, leading to programmed cell death. **c** The mechanism of ROS-mediated regulation of cell growth and carcinogenesis. In the G1 phase, ROS promote G1/S transition via CDK2 oxidation/phosphorylation while inhibiting cyclin D–CDK4/6 through disulfide bond formation, reducing proliferation. During S phase, mitochondrial ROS accelerate replication, and moderate ROS increase CDK2 activity, bypassing senescence and promoting immortalization. In the G2/M phase, sustained ROS inactivate Cdc25 phosphatases, block CDK1 activation, hyperphosphorylate Aurora A (disrupting spindle assembly), and oxidize APC/C components like APC11, prolonging mitotic arrest. ROS also drive carcinogenesis by activating major proliferative and survival pathways, including Wnt/β-catenin, PI3K/AKT/mTOR, ERK, AhR, TGF-β, Ca^2+^ signaling, EGFR, HIF1α, JAK/STAT, NF-κB, and Nrf2 pathways. For example, ROS increase β-catenin nuclear translocation to activate c-Myc and cyclin D1, and stimulate PI3K–AKT signaling, promoting survival, metabolism, and mTORC2 feedback activation. ERK-driven CREB phosphorylation increases growth-related gene expression. AhR-ARNT complexes bind XRE/DRE elements to regulate cell cycle genes, whereas TGF-β enhances cyclin D1 via β-catenin. Elevated Ca^2+^ activates DREAM and HSFs to trigger proliferation, and HIF1α promotes glycolysis and IGF-2 expression under hypoxia. EGFR activates the Ras-Raf-MAPK and PI3K-AKT pathways; JAK/STAT signaling promotes transcription of survival genes; and NF-κB upregulates cyclin D1 while inhibiting p53. Additionally, ROS-induced oxidative DNA damage activates the ATM/p53/p21 pathway, which may contribute to genomic instability and mutagenesis. This figure was created in BioRender. tonu, r. (2025) (https://BioRender.com/0q9bi92). CDK2 cyclin-dependent kinase, APC/C anaphase-promoting complex/cyclosome, APC11 anaphase-promoting complex subunit 11, ARNT aryl hydrocarbon receptor nuclear translocator, XRE/DRE xenobiotic/dioxin response element, DREAM dimerization partner, RB-like, E2F, and multivulval class B, HSFs heat shock factors, IGF-2 insulin-like growth factor 2, ATM ataxia-telangiectasia mutated

Redox homeostasis refers to the balance between the production of ROS and the antioxidant mechanisms that neutralize these ROS.^4^ While basal ROS levels support signaling and defense, mild oxidative stress damages biomolecules, increasing the risk of mutations and diseases like cancer.^57^ Conversely, excessive ROS overwhelm the cell’s antioxidant defenses, triggering oxidative damage and, ultimately, programmed cell death.^4^ To prevent such damage, cells maintain redox homeostasis through an elaborate antioxidant defense system (Fig. 2a, b).

Nrf2 plays a critical role in maintaining redox homeostasis. It is a redox-sensitive transcription factor that regulates the expression of antioxidant and ROS-detoxifying enzymes. Under oxidative stress, ROS modify the Keap1-Nrf2 interaction, allowing Nrf2 to escape degradation, accumulate in the nucleus, and activate AREs.^57^ This triggers the transcription of genes encoding antioxidants such as SOD, catalase, GSH, glutathione reductase (GR), GSH S-transferase (GSTP), GPX, TRXR, NADPH quinone oxidoreductase 1 (NQO1), and heme oxygenase-1 (HO-1), which neutralize ROS and protect cells against oxidative stress, especially in conditions such as cancer, where ROS levels are elevated^57^ (Fig. 1b).

When initiated, SOD catalyzes the dismutation of the superoxide anion into H_2_O_2_ and oxygen (O_2_), reducing the risk of oxidative damage from superoxide, a highly reactive ROS. While H_2_O_2_ is still a reactive molecule, it is less harmful and is further detoxified by catalase and GPX, thereby protecting cells from oxidative stress.^6^ The GSH system, where GSH is the primary intracellular antioxidant, neutralizes ROS and restores oxidized protein thiols, converting GSH into GSSG.^6^ The GSH/GSSG ratio reflects the redox state of the cell, with a high ratio indicating a healthy, reducing environment. Glutathione reductase regenerates GSH from GSSG using NADPH. Similarly, the TRX system maintains redox balance by reducing oxidized proteins and scavenging ROS. TRXR, also NADPH-dependent, keeps TRX in its reduced, active state.^58^ Both systems, involving enzymes like glutathione peroxidase and peroxiredoxin, are vital for neutralizing peroxides and preventing oxidative stress damage (Fig. 2a).

While Nrf2 signaling-mediated redox balance is beneficial for healthy cells, it also regulates redox homeostasis in cancer cells, ensuring that ROS do not reach toxic levels that could hinder cell proliferation.^59^

## ROS and biology of cancer

ROS, particularly H_2_O_2,_ can induce reversible oxidation of cysteine residues in several signaling proteins. H_2_O_2_ can also directly modulate the activities of several antioxidants, protein kinases, and transcriptional regulators for redox regulation. Besides, ROS trigger different phosphatases that dephosphorylate and thus inactivate kinases, thereby promoting signaling pathways. Therefore, ROS are key modulators of a wide array of pathways and transcription factors associated with cell proliferation, differentiation, immune response, epithelial-mesenchymal transition (EMT), and cell death. These pathways vary in subcellular localization: cytoplasmic-localized pathways include epidermal growth factor receptor (EGFR), extracellular-regulated kinase 1/2 (ERK1/2), ASK1, and ATG4, although some (such as ERK1/2 and EGFR) also exert nuclear effects upon activation.^60^ In contrast, nuclear-localized factors such as p53 and PARP1, upon ROS-mediated activation, function predominantly in transcription regulation and DNA repair, although neucleocytoplasmic shuttling of p53 is also well documented.^60,61^ A third redox-sensitive pathway group—including Wnt/β-catenin, PI3K/AKT/mTOR, aryl hydrocarbon receptor (AhR), TGF-β, calcium signaling, Janus kinase (JAK)/signal transducers and activators of transcription (STAT), hypoxia-inducible factor 1 α (HIF1α), NF-κB, Nrf2, c-Jun N-terminal kinase (JNK), p38, and Ataxia-telangiectasia mutated (ATM)—features components that shuttle between the cytoplasm and nucleus, allowing complex regulation of gene expression and cellular fate.^60^

In terms of cellular progression or defining cell fate, ROS-modulated prosurvival pathways include Wnt/β-catenin, PI3K/AKT/mTOR, AhR, TGF-β, calcium signaling, EGFR, ERK1/2, JAK/STAT, HIF1α, NF-κB, and Nrf2 pathways.^3^ The prodeath pathways modulated by ROS include JNK, p38, ASK1, p53, ATG4, PARP1, ATM, and AMP-activated protein kinase (AMPK) pathways.^3^ Prosurvival pathways, when hyperactivated, driven by moderate increase in ROS, are considered to induce pro-oncogenic phenomena, while prodeath pathways, when initiated under excessive ROS, are considered to exert antitumor activity. Interestingly, categorically assigning whether ROS activate or inhibit a specific pathway is overly simplistic. For example, while moderate ROS often drive tumorigenic processes by enhancing prosurvival signaling, high ROS levels can inhibit these same pathways, such as PI3K/AKT, leading to growth arrest or cell death.^62,63^ Again, it is not uncommon for prosurvival signaling pathways to also induce the expression of cell death-related molecules; for example, JAK/STAT signaling promotes proliferation but can also mediate necroptosis via ZBP1-RIPK3-MLKL activation in the absence of RIPK1.^64^ Thus, ROS-mediated signaling is highly context dependent, influenced by ROS concentration, cellular metabolic state, and intrinsic genetic factors, underscoring the complex and dynamic interplay between redox balance and cancer biology.

### ROS, cell cycle regulation, and carcinogenesis

ROS are key regulators of the cell cycle, with their effects determined by concentration, duration, and cellular context. In the G1 phase, ROS-mediated oxidation/phosphorylation of cyclin-dependent kinase 2 (CDK2) promotes its activation, facilitating the G1/S transition.^65^ Interestingly, Auranofin-induced H_2_O_2_ inhibits cyclin D-CDK4/6 activity by inducing disulfide bond formation between CDK4 and cyclin D, reducing cell proliferation.^66^ During the S phase, ROS modulate CDK2-cyclin A activity and intra-S phase checkpoints, with mitochondrial ROS accelerating replication onset.^67^ A moderate increase in ROS levels may increase CDK2 activity, which is vital for bypassing senescence and enabling cancer cell immortalization.^67^ Conversely, in the G2/M transition (particularly redox-sensitive), sustained ROS have also been reported to oxidize and inactivate Cdc25 phosphatases, preventing CDK1 activation and blocking mitotic entry, potentially leading to cell death.^68^ Moreover, excessive ROS hyperphosphorylate Aurora A, disrupting spindle assembly,^69^ and oxidize APC/C components like APC11, inhibiting cyclin B degradation and prolonging mitotic arrest.^70^

Additionally, physiological levels of ROS, particularly H_2_O_2,_ can modulate numerous prosurvival pathways and transcription factors involved in cell cycle progression. For example, Wnt/β-catenin signaling promotes β-catenin nuclear translocation, where it interacts with T-cell factor/lymphoid enhancer-binding factor (TCF/LEF) transcription factors to activate the expression of target genes regulating cell proliferation, such as c-Myc and cyclin D1.^71^ AhR translocates to the nucleus, forms a complex with ARNT, and binds to xenobiotic response elements (XREs) or dioxin response elements (DREs) located in the promoter regions of target genes involved in cell cycle progression and proliferation.^72^ However, the ability of the AhR pathway to induce a G1/S cell cycle block, as evidenced following human cytomegalovirus (HCMV) infection, highlights the need for further research considering its role in various cancers and cellular contexts.^72^ TGF-β enhances cyclin D1 transcription via β-catenin signaling, enhancing cyclin D1 promoter activity.^73^ Ca²⁺ signaling activates transcription factors such as the DREAM complex and heat shock transcription factors (HSFs), driving immediate early gene (IEG) expression.^74^ These IEGs are responsible for transitioning cells from a resting state (G0) back into the cell cycle, initiating proliferation. HIF1α promotes cell growth by enhancing glycolysis under hypoxia and regulating growth factors like IGF-2 to support proliferation and survival.^75^ Once activated, EGFR triggers multiple downstream pathways, including the Ras-Raf-MAPK pathway and the PI3K-AKT pathway, ultimately leading to cell division and growth.^76^ JAKs phosphorylate receptors and STAT proteins, leading to STAT dimerization and nuclear translocation to drive transcription of genes for growth, proliferation, and survival.^77^ NF-κB promotes the G1-S transition by upregulating cyclin D1 and inhibiting p53, driving cell growth and apoptosis resistance.^67^

The PI3K/AKT pathway is a key signaling cascade linked to cell proliferation and is often dysregulated in cancer. Regarding PI3K/AKT pathway, activated PI3K phosphorylates PIP2 into PIP3, which recruits AKT to the plasma membrane, where it is phosphorylated at Thr308 and Ser473, triggering full activation.^78^ AKT activation regulates cell survival, growth, and metabolism by inhibiting p27, activating cAMP-response element binding protein (CREB), inactivating tuberous sclerosis complex 2 (TSC2), sequestering FOXO in the cytoplasm, and engaging a feedback loop with mTORC2 (AKT and mTORC2 form a critical feedback loop in which mTORC2 activates AKT by phosphorylating it at serine (Ser)473. This increased AKT activity might subsequently promote further mTORC2 stimulation).^79^ These coordinated activities collectively promote protein synthesis and cell cycle progression. The ERK pathway activates kinases such as p90RSK and MSK1, which phosphorylate CREB at Ser133.^80^ When CREB is phosphorylated, it acts as a transcriptional activator of various genes involved in cell growth and proliferation, including c-Myc and CDK1. As described earlier, hyperactivation of these pathways due to increases in ROS levels may contribute to carcinogenesis.

Conversely, excessive ROS concentrations can exert an antitumor effect by either modulating prodeath signaling pathways or inhibiting prosurvival pathways. For example, supraphysiological levels of ROS induces G1 arrest by stabilizing c-Fos binding to chromatin, suppressing Fra-1-mediated cyclin D1 expression, and activating p21 through the ATM/p53 signaling pathway.^81^ During S-phase, oxidative DNA damage, including 8-oxoG lesions and strand breaks, activates the MRN-ATM-Chk2 cascade, halting replication to allow repair via p53/p21 activation.^82^ Besides, drug-induced ROS can inhibit the PI3K/AKT pathway to suppress cancer cell proliferation by disrupting key pathways critical for tumor growth and survival, as evidenced by studies with (+)-anthrabenzoxocinone in NSCLC and 6-methoxydihydrosanguinarine in breast cancer.^62,63^ While the exact basis for this paradoxical role remains unclear, excessive ROS levels induced by these drugs may underlie their inhibitory effects (Fig. 2c).

### ROS and angiogenesis

Elevated ROS levels (particularly NOX4-induced H_2_O_2_) promote angiogenesis through redox-mediated pathways, with HIF1α playing a central role by directly upregulating VEGF and angiogenic factors like angiopoietin-2 and endothelin-1 (EDN1), particularly in colon cancer.^83^ AKT-mediated FOXO inhibition prevents VEGF suppression while enhancing Sp1-driven VEGF transcription.^78^ Wnt/β-catenin signaling similarly activates VEGF and EDN1 expression via TCF/LEF complexes, as evidenced in HEK293T cells.^84^ In addition to activating VEGF, NF-κB initiates interleukin-8 (IL-8) and matrix metalloproteinase 9 (MMP9), and interacts with the PI3K pathway to enhance the expression of angiogenic factor with G patch and FHA domains 1 (AGGF1).^85^ TGF-β1 induces angiogenesis primarily by enhancing VEGF secretion under hypoxia and upregulating signaling proteins such as Smad3/4 and HIF1α, which further increase VEGF transcriptional activity through histone acetyltransferase (p300), emphasizing the role of chromatin remodeling.^86^ TGF-β1 also upregulates Flt-1, a key VEGF receptor, further amplifying the angiogenic response.

**Fig. 3:**
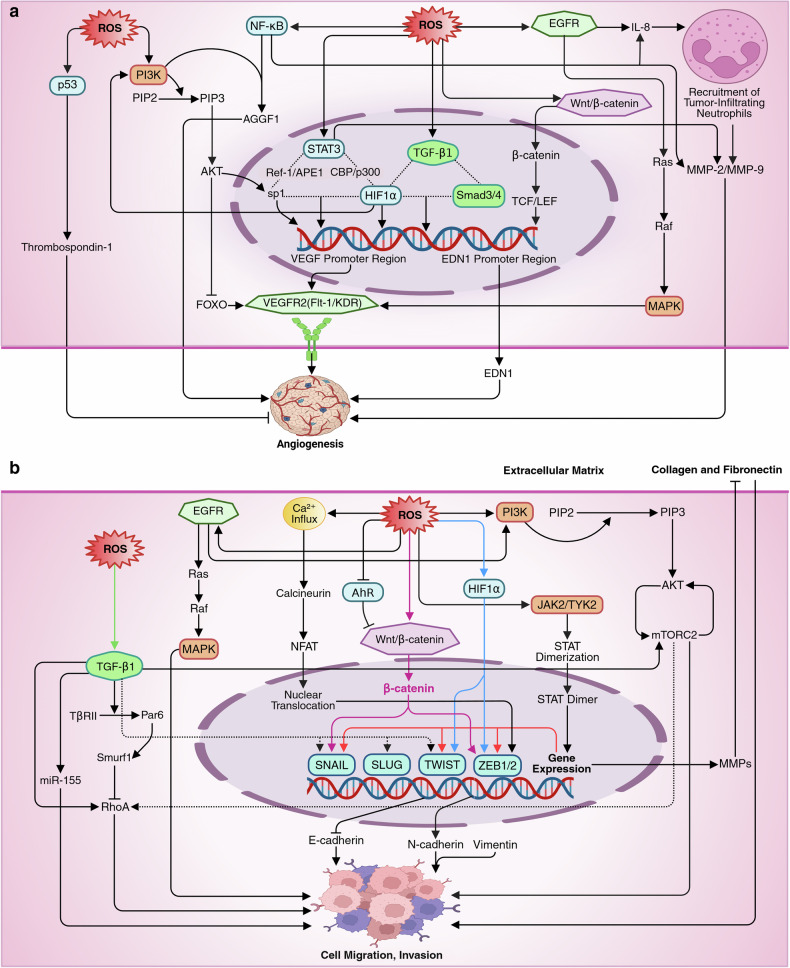
**a** The mechanism of the ROS-mediated induction of angiogenesis. ROS induce angiogenesis primarily through redox-mediated pathways, particularly under hypoxic conditions. HIF1α plays a central role by upregulating VEGF and other angiogenic factors, such as angiopoietin-2 and endothelin-1 (EDN1). AKT activation inhibits FOXO proteins, preventing VEGF suppression, while enhancing Sp1-mediated VEGF transcription. Stabilized β-catenin interacts with TCF/LEF complexes to drive VEGF and EDN1 expression. NF-κB enhances angiogenesis by inducing IL-8, MMP9, and AGGF1 expression. TGF-β1 increases VEGF secretion under hypoxia and facilitates promoter activation via Smad3/4 and HIF1α, supported by chromatin remodeling through p300. EGFR signaling promotes angiogenesis via the Ras-Raf-MAPK pathway or neutrophil-mediated VEGF activation. Additionally, under hypoxic conditions, STAT3 forms transcriptional complexes with HIF1α and co-factors such as Sp1 to enhance VEGF expression and regulate ECM degradation through MMP2 and MMP9, further driving vascular growth. **b** The mechanism of ROS-mediated induction of metastasis. ROS activate Wnt/β-catenin signaling, enabling the nuclear translocation of β-catenin and its interaction with transcription factors such as ZEB1/2 and SNAIL to repress epithelial markers and promote mesenchymal traits. PI3K/AKT signaling stimulates mTORC2, a key regulator of EMT. TGF-β signaling disrupts cell polarity through TβRII-mediated Par6 phosphorylation and Smurf1-dependent RhoA degradation, while inducing miR-155 to weaken tight junctions. Paradoxically, it also activates RhoA and mTORC2 to promote stress fiber formation, EMT, and motility via the upregulation of SNAIL, SLUG, and ZEB, driving mesenchymal transition. ROS-mediated calcium signaling activates calcineurin, which modulates NFAT to induce ZEB1/2 expression, thereby enhancing mesenchymal marker expression (e.g., N-cadherin and vimentin) while repressing E-cadherin. EGFR signaling triggers the Ras-Raf-MAPK and PI3K-AKT pathways to regulate EMT markers, whereas hypoxia-induced HIF1α activates transcription factors such as TWIST and ZEB1 to induce mesenchymal traits. Additionally, activated STATs enhance EMT by upregulating matrix metalloproteinases (MMPs) and EMT-related transcription factors, such as SNAIL, TWIST, and ZEB1. This figure was created in BioRender. tonu, r. (2025) (https://BioRender.com/0q9bi92). VEGF vascular endothelial growth factor, FOXO forkhead box O (transcription factor), TCF/LEF T-cell factor/Lymphoid enhancer factor, MMP9 matrix metalloproteinase-9, AGGF1 angiogenic factor with G-patch and FHA domains 1, ZEB1/2 zinc finger E-box-binding homeobox 1/2, NFAT nuclear factor of activated T cells, TWIST twist-related protein (transcription factor)

EGFR signaling promotes the Ras-Raf-MAPK pathway or recruits tumor-infiltrating neutrophils via IL-8, which enhances the permeability of newly formed vessels, facilitating tumor cell migration.^76^ Neutrophils also deliver a specialized form of MMP9 that enzymatically activates VEGF. Under hypoxia, STAT3 forms transcriptional complexes with HIF1α, CBP/p300, and Ref-1/APE to enhance VEGF expression, as observed in multiple cancers.^87^ STAT3 also directly binds to the VEGF promoter, as observed in NSCLC, and regulates MMP2 and MMP9 secretion, facilitating ECM degradation and angiogenesis.^88^ Additionally, cofactors like HIF1α and Sp1 collaborate with STAT3 in endothelial cells to further promote VEGF expression, supporting angiogenesis^87^ (Fig. 3a).

Conversely, excessive ROS can inhibit VEGF signaling by damaging receptors and depleting NO• through ONOO^−^ formation, disrupting vasodilation and endothelial function.^89^ ROS may also activate antiangiogenic pathways, such as p53-mediated production of thrombospondin-1.^90^ Furthermore, elevated ROS levels can trigger endothelial apoptosis via mitochondrial dysfunction and caspase activation.^91^ The ability of excessive ROS to inhibit angiogenesis is now being leveraged in the development of ROS-mediated cancer therapeutics.

### ROS and metastasis

Moderate ROS levels promote metastasis and cancer cell invasion by hyperactivating prosurvival pathways like Wnt/β-atenin pathway, which drives EMT through β-catenin’s nuclear interaction with transcription factors such as ZEB1/2 and SNAIL to repress epithelial markers.^92^ PI3K/AKT activation triggers mTORC2, a key regulator of EMT,^79^ whereas TGF-β signaling disrupts cell polarity via Par6 phosphorylation by TGF-β type II receptor (TβRII) and RhoA degradation, which is mediated by Smurf1, and induces miR-155 expression to destabilize tight junctions.^93^ Interestingly, TGF-β signaling activates RhoA, promoting stress fiber formation to enhance migration and invasion, while its activation of mTORC2 further supports RhoA activity, facilitating EMT and cell motility.^94^ Additionally, TGF-β signaling activates SNAIL, SLUG, and ZEB, thus promoting a more migratory mesenchymal phenotype.^94^ Calcium signaling through calcineurin activates the nuclear factor of activated T cell (NFAT), which translocates into the nucleus to induce ZEB1/2 expression, modulating E-cadherin downregulation and N-cadherin and vimentin upregulation.^95^ EGFR activation triggers the MAPK and PI3K-AKT pathways, which directly regulate the expression of mesenchymal and epithelial markers.^76^ HIF1α drives EMT via TWIST and ZEB1,^96^ whereas activated STATs promote MMP expression and the expression of EMT-associated transcription factors such as SNAIL, TWIST, and ZEB1.^88^ A recent study reported that chronic oxidative stress disrupts the circadian rhythm of neutrophils and promotes neutrophil extracellular trap (NET) formation via glucocorticoid release, creating a metastasis-supporting microenvironment.^97^

Conversely, excessive ROS have been well documented to inhibit metastasis in several studies. GPX2-knockdown-induced ROS suppress gastric cancer progression and metastasis by disrupting the KYNU-Kyn-AhR signaling pathway,^98^ which is known to promote metastasis, as evidenced in chronic lymphocytic leukemia (CLL) in mice.^99^ Similarly, a cholesterol oxidase-loaded Co–PN3 single-atom nanozyme effectively suppresses tumor metastasis by enhancing ROS generation^100^ (Fig. 3b).

### ROS and drug resistance

**Fig. 4:**
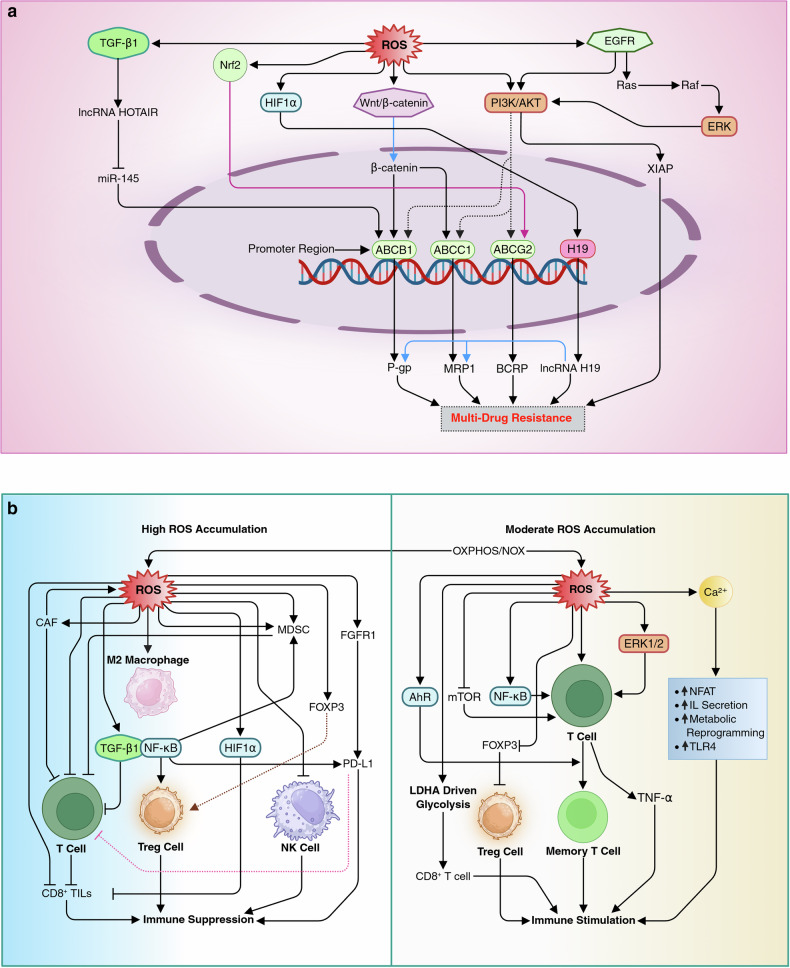
**a** The mechanism of ROS-mediated induction of drug resistance. Elevated levels of ROS are linked to the upregulation of drug efflux pumps such as P-glycoprotein (P-gp) and MRP1 through the regulation of multiple signaling pathways, including Wnt/β-catenin, EGFR, ERK, TGF-β, PI3K/AKT, HIF1α, and MAPK pathways. ROS-induced β-catenin translocates to the nucleus, where it enhances the expression of drug resistance genes such as ABCB1 (P-gp) and ABCC1 (MRP1) by interacting with transcription factors. The PI3K/AKT pathway further drives multidrug resistance by upregulating ABC transporters, including ABCG2 (BCRP), and activating antiapoptotic proteins such as XIAP, which promote tumor survival, proliferation, and metastasis. Hypoxia-induced HIF1α increases P-gp and MRP1 expression via H19 transcription, with TGF-β1 contributing through the HOTAIR/miR-145 axis. Additionally, the activation of Nrf2 by ROS promotes drug efflux by enhancing ABCG2 transporter expression. EGFR and ERK pathways also contribute to the upregulation of P-gp and MRP1 via PI3K signaling, further enhancing the resistance phenotype. **b** Interplay between ROS and immunomodulation. In the tumor microenvironment, excessive ROS accumulation results in immune suppression, impairing T-cell responses, promoting the apoptosis of cytotoxic CD8^+^ tumor-infiltrating lymphocytes and natural killer cells, and upregulating immune checkpoints such as PD-L1 via FGFR1 signaling, thus suppressing T-cell activity and enabling immune evasion of cancer cells. ROS also favor the survival of immunosuppressive cells like myeloid-derived suppressor cells and M2 macrophages, further suppressing immune responses. ROS contribute to immune dysfunction by impairing T-cell receptor functionality. Additionally, ROS enhance the TGF-β/Smad2/3 pathway and induce NF-kB activation, suppressing antitumor immunity, promoting Treg and MDSC expansion, and stabilizing HIF1α, which fosters a hypoxia-driven immunosuppressive microenvironment, thereby promoting cancer progression and immune evasion. Moderate ROS play a pivotal role in immune stimulation by promoting T-cell proliferation and interleukin secretion. Moderate ROS levels activate nuclear factors of activated T cells, stimulating IL-2 and IL-4 secretion, while ROS can also revitalize exhausted CD8^+^ T cells through LDHA-driven glycolysis. ROS modulate key TCR signaling pathways, including calcium, NF-kB, ERK1/2, and mTOR pathways, enhancing T-cell activation, proliferation, and metabolic reprogramming. Furthermore, ROS-mediated activation of AhR supports the transition of effector T cells into memory T cells. Additionally, ROS reduce FOXP3 stability, thereby decreasing suppressive function of Tregs. This figure was created in BioRender. tonu, r. (2025) (https://BioRender.com/0q9bi92). P-gp P-glycoprotein, MRP1 multidrug resistance-associated protein 1 (encoded by ABCC1), ABC ATP-binding cassette, XIAP X-linked inhibitor of apoptosis protein, FGFR1 fibroblast growth factor receptor 1, Treg regulatory T cell, MDSC myeloid-derived suppressor cell, LDHA lactate dehydrogenase A, TCR T-cell receptor, FOXP3 forkhead box P3

Moderate levels of ROS are linked to the upregulation of drug efflux pumps like P-gp and MRP1. ROS regulate several signaling pathways involved in regulating drug efflux pumps. For example, β-catenin, after nuclear translocation, upregulates the expression of drug resistance genes such as ABCB1 (encoding P-gp) and ABCC1 (encoding MRP1).^101^ The PI3K/AKT pathway promotes multidrug resistance by upregulating ABCB1, ABCC1, and ABCG2 (encoding BCRP), and activating antiapoptotic proteins like X-linked inhibitor of apoptosis (XIAP).^102^ HIF1α activates H19 transcription in NSCLC cells, with long noncoding RNA H19 overexpression linked to increased P-gp and MRP1 expression.^103^ Furthermore, the EGFR and ERK pathways induce p-gp expression by activating PI3K signaling, while TGF-β1 enhances P-gp through the HOTAIR/miR-145 axis, providing a novel mechanism for drug resistance in cancer.^104,105^ A study demonstrated that oxidative stress-induced Nrf2 promotes drug efflux by upregulating ABCG2, as observed in biliary tract cancer cells.^106^ Interestingly, targeting the Nrf2 pathway to overcome drug resistance in liver cancer involves the induction of excessive ROS.^107^ Another recent study suggested that the 2D-CuPd nanozyme overcomes Tamoxifen resistance in breast cancer by leveraging the ROS-mediated inhibition of the PI3K/AKT/mTOR pathway.^108^ Many other studies are also exploring the vulnerability of cancer cells to excessive ROS to overcome drug resistance. (Fig. 4a).

### ROS in immunosuppression

In the tumor microenvironment (TME), excessive ROS exert profound immunosuppressive effects, undermining antitumor immunity and promoting cancer progression. Cancer cells, cancer-associated fibroblasts (CAFs), and myeloid-derived suppressor cells (MDSCs) are major contributors to elevated ROS levels, which suppress T-cell responses and promote the apoptosis of T cells due to their weak antioxidant systems.^109^ ROS impair the function of cytotoxic CD8^+^ tumor-infiltrating lymphocytes (TILs) by inducing mitochondrial dysfunction, which reduces their ability to attack cancer cells.^110^ Similarly, natural killer (NK) cells are rendered less effective, as ROS increase their apoptosis and functional impairment.^111^ Furthermore, ROS contribute to the upregulation of immune checkpoint molecules like PD-L1 on cancer cells via FGFR1 signaling, suppressing T-cell activity and enabling immune evasion.^112^ ROS also favor the survival of immunosuppressive cells such as MDSCs and M2 macrophages, which have enhanced antioxidant capacities, allowing them to thrive under oxidative conditions.^113^ Through nitration and nitrosylation, ROS impair antigen presentation and TCR functionality, exacerbating immune dysfunction.^114^ Hydrogen sulfide, an RSS, promotes regulatory T (Treg) cell differentiation by activating FOXP3.^115^ ROS enhance the TGF-β/Smad2/3 pathway and induce NF-κB activation, collectively suppressing antitumor immunity by inhibiting cytotoxic T cells and NK cells, promoting the expansion of Tregs and MDSCs, and upregulating immune checkpoints like PD-1 and PD-L1.^116^ Additionally, ROS stabilize HIF1α, promoting immunosuppression in NSCLC through inducing EMT, reducing CD8^+^ TILs, and activating the HIF1α/LOXL2 pathway, which fosters a hypoxia-driven immunosuppressive microenvironment^117^ (Fig. 4b).

Conversely, the immunostimulatory role of moderate levels of ROS is also well evidenced. ROS, including O_2_•⁻, H_2_O_2_, and OH•, serve as critical signaling molecules that bolster immune responses through their role in both innate and adaptive immunity. Upon T-cell receptor (TCR) activation, mitochondrial oxidative phosphorylation (OXPHOS) and NOXs are upregulated, leading to increased ROS production, which fuels T-cell proliferation and interleukin (IL) secretion.^118^ Moderate ROS levels also activate NFAT to stimulate IL-2 secretion, promoting T-cell proliferation and survival.^119^ Besides, ROS can also induce the type 2 cytokine Fc–IL-4, which revitalizes exhausted CD8^+^ T cells by increasing LDHA-driven glycolysis and NAD+ generation, effectively restoring their antitumor activity.^120^ ROS play crucial modulatory roles in amplifying distal TCR pathways, including Ca^2+^-calcineurin-NFAT, IKK-NF-κB, Ras-ERK1/2, and mTOR cascades, which in turn enhance T-cell activation, proliferation, and cytokine production (e.g., IL-2).^121^ Additionally, ROS-activated AhR in effector T cells supports their transition into memory T cells, ensuring long-term immune surveillance.^122^ Peroxynitrite stimulates TLR4 and NF-κB activation and cytokines (release of TNF-α, IL-1β, and IL-8)^123^, and NOX-induced ROS play a role in inflammasome activation, such as the NLRP3 inflammasome, which drives caspase activity and cytokine production to enhance immune cell recruitment and function.^124^ Additionally, the loss of transmembrane p24 trafficking protein 4 (TMED4)-induced ROS reduces FOXP3 stability and the suppressive function of Tregs in an IRE1α/XBP1 axis–dependent manner^125^ (Fig. 4b).

### ROS, cellular metabolism and cancer

#### Carbohydrate metabolism

**Fig. 5:**
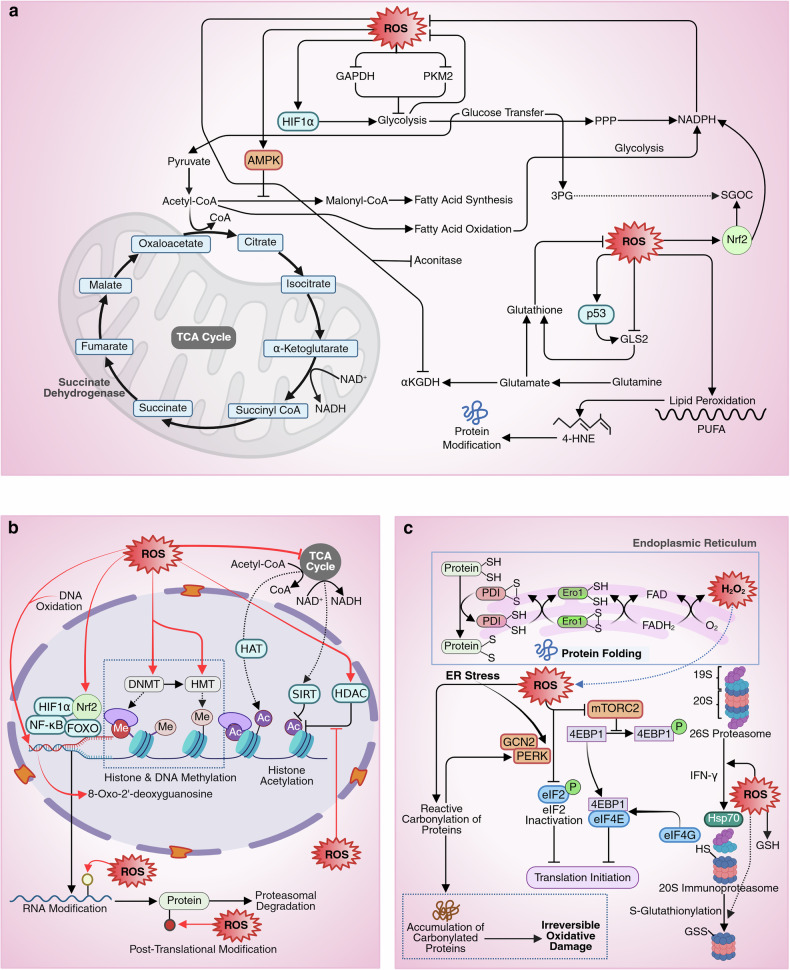
**a** ROS in carbohydrate and lipid metabolism. ROS modulate cancer metabolism by altering key pathways essential for tumor survival and proliferation. In glycolysis, ROS oxidize GAPDH and PKM2, inhibiting their activity and redirecting glucose flux from glycolysis to the oxidative pentose phosphate pathway (PPP), thereby increasing NADPH production to combat oxidative stress. Simultaneously, ROS stabilize HIF1α, enhancing glucose uptake and metabolism. In mitochondria, ROS impair TCA cycle enzymes such as aconitase and α-ketoglutarate dehydrogenase, and superoxide from the ETC amplifies redox signaling. In glutaminolysis, ROS oxidize glutaminase and, via p53 activation, upregulate GLS2 to support glutathione regeneration. ROS also influence serine‒glycine one-carbon metabolism (SGOC), with Nrf2 upregulating this pathway to generate NADPH and nucleotides. In lipid metabolism, elevated ROS activate AMPK, which inhibits ACC, reducing malonyl-CoA levels and increasing fatty acid oxidation (FAO). This shift sustains ATP and NADPH production, supporting tumor survival and therapy resistance. However, ROS also induce lipid peroxidation, attacking PUFAs, generating 4-HNE, which forms toxic adducts with proteins, leading to dysfunction. **b** ROS in nucleic acid metabolism. ROS significantly impact nucleic acid metabolism through direct oxidative damage and modulation of transcriptional, epigenetic, and posttranscriptional processes. ROS regulate redox-sensitive transcription factors such as Nrf2, NF-κB, HIF1α, and FOXO via posttranslational modifications (PTMs) or upstream signaling pathways, influencing the expression of genes and ncRNAs. ROS-induced activation of Nrf2 leads to the expression of ncRNAs, some of which promote tumorigenesis, therapeutic resistance or regulated cell death. Epigenetically, ROS alter DNA and histone methylation and acetylation through effects on enzymes like DNMTs, HATs, HDACs, and sirtuins. Redox modifications such as S-nitrosylation and S-glutathionylation modulate chromatin structure and gene accessibility, while mitochondrial ROS influence the NAD^+^/NADH ratio, which governs sirtuin activity. Furthermore, ROS induce epigenetic regulation through oxidative DNA lesions such as 8-oxo-dG and promote RNA modifications, including N6-methyladenosine, which alter mRNA stability and translation, contributing to drug resistance. **c** ROS in protein metabolism. Redox signaling tightly regulates protein metabolism by modulating protein synthesis, folding, and degradation. H₂O₂ impairs protein synthesis by activating oxidative stress-sensitive kinases such as PERK and GCN2, leading to eIF2 phosphorylation and translation repression, and by inhibiting mTORC1, which stabilizes 4EBP1–eIF4E interaction, blocking translation initiation. In the ER, protein folding depends on redox-regulated disulfide bond formation catalyzed by Ero1 and PDI, both using redox-active cysteines. H_2_O_2_ also influences protein degradation by enhancing immunoproteasome formation via IFN-γ signaling and promoting 20S proteasome activity through S-glutathionylation. Additionally, ROS induce protein carbonylation—irreversible oxidative damage, disrupting proteostasis and driving tumor progression. However, excessive ROS can also trigger ER stress, which overwhelms cancer cell defenses, exerting potential antitumor effects. This figure was created in BioRender. tonu, r. (2025) (https://BioRender.com/0q9bi92). GAPDH glyceraldehyde-3-phosphate dehydrogenase, PKM2 pyruvate kinase M2, αKGDH α-ketoglutarate dehydrogenase, GLS2 glutaminase 2, ACC acetyl-CoA carboxylase, PERK protein kinase R-like ER kinase, GCN2 general control nonderepressible 2, eIF2 eukaryotic initiation factor 2, 4EBP1 eukaryotic translation initiation factor 4E-binding protein 1, eIF4E eukaryotic translation initiation factor 4E, Ero1 endoplasmic reticulum oxidoreductin 1, PDI protein disulfide isomerase

ROS modulate cancer metabolism by influencing multiple pathways critical for tumor survival and proliferation. In glycolysis, ROS oxidize GAPDH and PKM2, altering their activity, thereby inhibiting glucose uptake, while simultaneously enhancing glucose uptake and metabolism through HIF1α stabilization.^126^ Interestingly, inhibition of GADPH and PKM2 redirects glucose-6-phosphate (G6P) flux away from glycolysis and towards the oxidative arm of the PPP, which produces NADPH, thereby boosting NADPH production to counter oxidative stress.^127^ In mitochondria, ROS impact the TCA cycle by oxidizing enzymes such as aconitase and α-ketoglutarate dehydrogenase (αKGDH), modulating metabolic flux, whereas superoxide generated at the ETC amplifies redox signaling.^128^ Glutaminolysis supports ROS detoxification by enhancing glutathione regeneration. ROS directly oxidize the glutaminase protein, altering its structure and function, and modulating metabolic pathways, including elevated mitochondrial ROS production, which further affects glutaminase activity. Interestingly, ROS can increase glutaminase activity by activating p53, which increases GLS2 expression and activity.^129^ Additionally, serine‒glycine one-carbon metabolism (SGOC) provides NADPH and nucleotides, with Nrf2 upregulating this pathway to mitigate oxidative damage.^130^ Cancer cells balance ROS levels to promote redox signaling and survival while avoiding oxidative stress, a vulnerability that therapies targeting these pathways aim to exploit, selectively inducing oxidative damage and cell death in tumors (Fig. 5a).

#### Lipid metabolism

Elevated ROS activate AMPK, which phosphorylates and inhibits acetyl-CoA carboxylase (ACC), reducing malonyl-CoA levels and thereby relieving the inhibition of carnitine palmitoyltransferase 1 (CPT1), the rate-limiting enzyme in fatty acid oxidation (FAO).^131^ This metabolic shift promotes FAO, leading to increased ATP production and regeneration of NADPH via TCA cycle-linked pathways. In many tumors, enhanced FAO provides metabolic flexibility, sustaining ATP and NADPH levels during oxidative and metabolic stress, which promotes survival, therapy resistance, and metastatic potential.^132^ Concurrently, ROS, particularly OH•, initiate lipid peroxidation by attacking polyunsaturated fatty acids (PUFAs) within cellular membranes. This peroxidation process leads to the formation of 4-hydroxy-2-nonenal (4-HNE).^133^ 4-HNE readily forms covalent adducts with nucleophilic amino acid residues—particularly cysteine, histidine, and lysine—through Michael addition or Schiff base formation, frequently resulting in protein misfolding, unfolding, or aggregation. Notably, in glioma cells, fatty acid-binding protein 7 (FABP7) binds and internalizes PUFAs to promote lipid droplet formation, whereas deletion of FABP7 decreases lipid droplets accumulation and leads to elevated ROS levels^6^ (Fig. 5a).

#### Nucleic acid metabolism

ROS significantly influence nucleic acid metabolism through direct oxidative modifications and by regulating transcriptional, epigenetic, and posttranscriptional mechanisms.

##### Redox regulation of transcription

ROS modulate the activity of redox-sensitive transcription factors (TFs), such as Nrf2, NF-κB, HIF1α, and FOXO, either through posttranslational modifications (PTMs) or by impacting upstream signaling pathways (such as Keap1 in Nrf2 signaling, IKK in NF-κB signaling, and PHD2 in HIF1α signaling).^134^ Under ROS stress, Nrf2 binds to ARE, which then induces noncoding RNA (ncRNA). While ncRNAs play a major role in therapeutic resistance, they can function as regulated cell death (RCD) accelerators that modulate therapeutic sensitivity. Besides, translation of ncRNAs can produce unwanted peptides in ROS-associated diseases, including cancer. Aberrant polyadenylation events within introns of tumor suppressor genes such as DICER1 and FOXN3 result in the production of truncated proteins lacking essential tumor-suppressive functions, thereby promoting tumorigenesis.^135^ Additionally, certain long ncRNAs (lncRNAs) can be translated into peptides bearing hydrophobic C-terminal tails, enabling their localization to cellular membranes, where they may act as tumor-associated antigens.^136^ Interestingly, normal cells and cancer cells seem to have different lncRNA transcripts in response to H_2_O_2_ stress, which might contribute to the different sensitivities to ROS observed in normal and cancer cells. ROS-induced PTMs also activate NF-κB signaling, leading to the upregulation of the antiapoptotic proteins BCL-XL and IAP^137^ (Fig. 5b).

##### Redox regulation of epigenetics

Redox signaling exerts long-term cellular effects through epigenetic mechanisms, including DNA and histone methylation, acetylation, and other PTMs. Mito-ROS regulate the expression of DNA methyltransferases (DNMTs), leading to changes in the expression of oxidative stress-related genes.^138^ Furthermore, the methyl donor of epigenetic methylation is from methionine metabolism, which can also be regulated by H_2_O_2_. Acetylation of histones at lysine residues serves as a transcriptional activator. This reaction is catalyzed by histone acetylases (HATs) using acetyl-CoA as a donor substrate. Histone deacetylases (HDACs), on the other hand, are regulated by redox modifications—for instance, S-nitrosylation of HDAC2 at cysteine residues induces its release from chromatin, which promotes chromatin remodeling and transcriptional activation of nearby genes.^9^ Furthermore, S-glutathionylation of histone 3 affects nucleosome stability and alters chromatin structure, which enhances the binding of the replication machinery to DNA.^139^ Sirtuins are an additional class of NAD^+^-dependent HDACs whose activity is indirectly redox-regulated through their dependence on the NAD^+^/NADH ratio maintained by the TCA cycle and affected by mitochondrial ROS production.^140^ Besides, 8-oxo-2′-deoxyguanosine, a product of DNA oxidation, functions as an epigenetic modifier that regulates gene expression. Additionally, RNA modification is a posttranscriptional event that can be regulated by ROS.^6^ The generation of N6-methyladenosine is a very common type of RNA modification affected by H_2_O_2_ through the activation of methyltransferases or demethylases.^141^ Notably, N6-methyladenosine modification can alter the stability and translational efficiency of mRNAs, leading to drug resistance (Fig. 5b).

#### Protein metabolism

Redox signaling intricately regulates protein metabolism at multiple stages, including protein synthesis, folding, and degradation. ROS, particularly H_2_O_2_, negatively influence protein synthesis by inducing phosphorylation of eukaryotic initiation factor (eIF2) via oxidative stress-sensitive kinases such as PERK and GCN2, thereby repressing translation.^142^ Additionally, H_2_O_2_ suppresses mTORC1 activity, reducing phosphorylation of 4EBP1, which then binds eIF4E, preventing interaction with eIF4G, thereby blocking translation initiation. In the ER, protein folding is redox-regulated through the formation of structural disulfide bonds, which are catalyzed by Ero1 and PDI, both of which utilize redox-active cysteine residues.^143^ H_2_O_2_ also modulates protein degradation by promoting the formation of the immunoproteasome through IFN-γ signaling, which involves dissociation of the 19S regulatory particle from the 20S core, assisted by Hsp70.^144^ Moreover, H_2_O_2_ enhances proteasome activity via S-glutathionylation of cysteine residues in the 20S subunit.^145^ ROS also induce protein carbonylation by oxidizing amino acid residues such as proline, arginine, lysine, and threonine, forming reactive carbonyl groups—markers of irreversible oxidative damage.^146^ Accumulation of carbonylated proteins disrupts proteostasis and contributes to tumor progression; however, excessive ROS-induced ER stress can also exert antitumor effects by overwhelming cancer cell survival mechanisms (Fig. 5c).

### ROS and cell death

The specific type of RCD that cancer and immune cells undertake in response to ROS stress is determined by the intrinsic genetic wiring, metabolic state, and level and type of ROS. Intrinsic apoptosis occurs in stressed epithelial or cancer cells via mitochondrial BAX/BAK activation and caspase-9-mediated pathways when antioxidant defenses fail but the apoptotic machinery remains intact.^147^ Superoxide and hydroxyl radicals drive intrinsic apoptosis by disrupting mitochondrial membrane potential, whereas H_2_O_2_ promotes extrinsic apoptosis by inducing c-FLIP ubiquitination and proteasomal degradation, enabling caspase-8 activation. Extrinsic apoptosis dominates as ROS synergize with death receptor signaling (e.g., TNFR/Fas), activating caspase-8 through DISC formation.^148^

Necroptosis and extrinsic apoptosis share initial signaling pathways involving death receptors such as TNFR1 or Fas, but necroptosis occurs in caspase-8-deficient contexts (e.g., inflamed tissues or resistant cancers) via RIPK1/RIPK3/MLKL activation.^149^ Under specific conditions, ROS can bypass these pathways to induce pyroptosis, driven by inflammasome activation (e.g., NLRP3) in response to PAMPs/DAMPs, the predominance of inflammatory caspases (caspase-1/4/5/11) over caspase-8 or RIPK3, and cell-specific mechanisms prioritizing cytokine release (IL-1β/IL-18) in immune cells like macrophages.^150^ In such cases, ROS-activated inflammasomes cleave gasdermin-D to form membrane pores, enabling pyroptosis while suppressing apoptosis and necroptosis through mechanisms like c-FLIP degradation or RIPK1 ubiquitination.

Ferroptosis, characterized by GPX4 inhibition and iron-dependent lipid peroxidation, occurs in iron-rich cells such as glioblastomas and neurons, and is often triggered by decreased SLC7A11 expression or cystine depletion.^151^ In contrast, disulfidptosis arises in cancer cells under glucose deprived conditions with high SLC7A11 expression, leading to cystine accumulation, disulfide stress, and cytoskeletal collapse, ultimately resulting in cell death.^152^

Oxeiptosis occurs when extreme oxidative damage causes lysosomal membrane permeabilization, particularly when other death pathways are impaired.^153^ In contrast, cuproptosis, which is regulated by cuproptosis-related genes (CRGs), is promoted by genes such as FDX1, LIAS, and PDHA1, which increase cellular sensitivity, whereas genes like MTF1 and CDKN2A reduce sensitivity.^154^ Cells with mutations affecting Fe-S cluster formation or stability are especially vulnerable to cuproptosis because of weakened mitochondrial function and increased susceptibility to copper-induced toxicity.^155^ And ROS-induced NETosis occurs when tumor-derived factors like cytokines and DAMPs activate neutrophils via NOX2, resulting in excessive ROS production.^156^

### Apoptosis

Excessive intracellular ROS activate proapoptotic BH3-only proteins (e.g., BAD, BIM, PUMA, NOXA), which displace antiapoptotic BCL-2 family members (e.g., BCL-2, BCL-XL), enabling BAX/BAK activation and mitochondrial outer membrane permeabilization (MOMP).^157^ This releases cytochrome c and apoptogenic factors such as AIF, Smac/Diablo, and endonuclease G, initiating apoptosome formation and caspase (-9, -3, -6, and -7) activation.^158^ Effector caspases then cleave cellular proteins, causing DNA fragmentation, cytoskeletal dismantling, and controlled cell death.

In addition, ROS modulate several signaling pathways to promote apoptosis. These pathways include the ASK1 pathway, which initiates proapoptotic signaling through JNK and p38 pathways, leading to the phosphorylation of proapoptotic proteins like BAD, BIM, and BAX.^159^ PARP1 also activates BAX and BAK, contributing to apoptosis via mitochondrial dysfunction and chromatin remodeling.^160^ Additionally, PARP1 drives apoptosis via interactions with signaling molecules such as apoptosis-inducing factor (AIF), p53, caspase-3, caspase-7, and ATM.^161^ ATG4 plays a significant role in apoptosis, as ATG4 may shift cells from survival to apoptosis when autophagy is inhibited.^162^ Additionally, p53, through its activation by AMPK, ATM and other stress-related pathways, promotes BAX, PUMA, and NOXA,^163^ whereas TGF-β enhances the expression of BIK while downregulating BCL-XL, causing an increased apoptotic response.^164^ HIF1α binds to the hypoxia-responsive element (HRE) of the BCL-2 interacting protein 3 (BNIP3) gene, upregulating the expression of this apoptosis-inducing factor in various cancers.^165^ ROS also interact with calcium signaling at the mitochondrial–ER interface, where Ca^2+^ surges induce mitochondrial swelling and rupture via disrupted ion exchange and adenine nucleotide translocase (ANT) channel dysfunction.^166^ Besides, Ca^2+^ overload activates calpain, which cleaves BCL-2 proteins and activates BID and AIF, thereby promoting apoptosis. Ca^2+^-activated calcineurin dephosphorylates BAD, enhancing its proapoptotic activity.^166^

ROS promote extrinsic apoptosis by accelerating the ubiquitin-mediated degradation of c-FLIP, thereby enhancing DISC formation and caspase-8 activation through adapter proteins such as Fas-associated death domain (FADD) and TNF receptor-associated death domain (TRADD). ROS also activate pathways such as the AhR, p53, JNK, and ASK1 pathways, enabling extrinsic apoptosis. Recent findings revealed that ROS-driven AhR/CYP1B1 signaling induces Fas via p53 activation, contributing to 6PPDQ-induced cardiac dysfunction in zebrafish embryos.^167^ Additionally, p53 can bypass classical death receptors by upregulating Reprimo (RPRM), which activates the Hippo–YAP/TAZ–p73 axis, leading to the expression of proapoptotic genes.^168^ JNK and ASK1 modulate death receptor signaling, amplifying extrinsic apoptosis.^169^

**Fig. 6:**
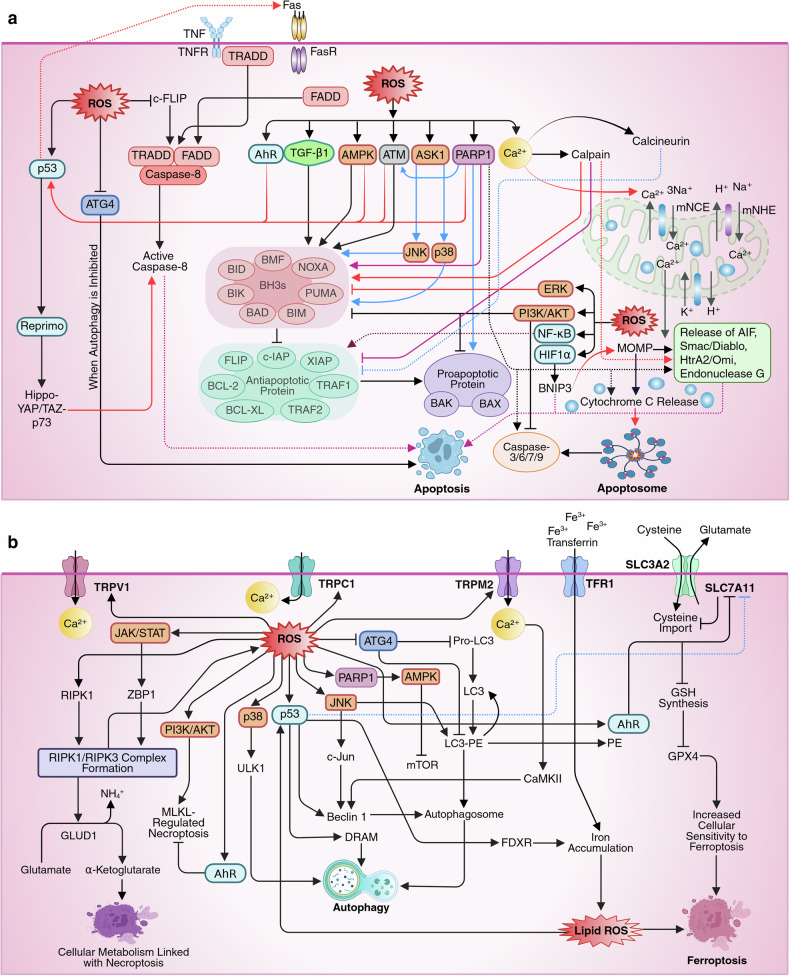
**a** The mechanism of ROS-mediated induction of apoptosis. Excessive intracellular ROS induce apoptosis by modulating key signaling molecules, such as ASK1, PARP1, ATG4, p53, JNK, p38, AMPK, ATM, and calcium signaling, in a context-dependent manner. In the intrinsic (mitochondrial) pathway, ROS activate proapoptotic BH3-only proteins (e.g., BAD, BID, BIM, PUMA, NOXA), displacing antiapoptotic BCL-2 family members (e.g., BCL-2, BCL-XL) and triggering mitochondrial outer membrane permeabilization (MOMP). This leads to cytochrome c release, apoptosome formation, caspase-9 activation, and downstream executioner caspases (3, 6, 7), with AIF, Smac/Diablo, and HtrA2/Omi further amplifying apoptosis. ASK1 activates JNK/p38, enhancing BAX activation via phosphorylation of BAD, BIM, and BAX. PARP1 promotes apoptosis through BAX/BAK activation and chromatin remodeling, while its cleavage by caspases conserves ATP. ROS-induced mitochondrial Ca^2+^ overload activates calpain- and AIF-mediated DNA fragmentation; calcineurin dephosphorylates BAD, inhibiting BCL-XL. In contrast, moderate ROS levels activate survival pathways (PI3K/AKT, ERK, and NF-κB). ROS also promote extrinsic apoptosis by degrading c-FLIP, enhancing DISC formation and caspase-8 activation via FADD and TRADD. Additionally, ROS activate AhR/p53 signaling to induce Fas, and p53 can trigger extrinsic-like apoptosis via Reprimo-mediated Hippo–YAP/TAZ-p73 activation. **b** The mechanism of ROS-mediated induction of autophagy, necroptosis, and ferroptosis. ROS regulate multiple cell death and survival pathways in a context-dependent manner. During autophagy, H_2_O_2_ oxidizes ATG4 under starvation, enhancing LC3/ATG8 lipidation and autophagosome formation. ROS also activate MAPK pathways, with JNK upregulating Beclin 1 and modulating LC3, whereas p38β phosphorylates ULK1 (Ser555) to initiate autophagy, especially in cancer. p53 induces the expression of autophagy-related genes (Beclin 1, DRAM), and ROS-activated TRP channels (e.g., TRPM2) promote Beclin 1 phosphorylation via CaMKII. PARP1 activation from DNA damage triggers AMPK, suppresses mTOR, and promotes autophagy. In necroptosis, ROS enhance RIPK1/RIPK3 aggregation and necrosome formation, while sustained JAK/STAT signaling upregulates ZBP1, driving necroptosis via MLKL or apoptosis via caspase-8. RIPK3 also links necroptosis to metabolism through GLUD1. Additionally, ROS might induce necrosis by initiating the PI3K/AKT pathway. In ferroptosis, ROS inhibit the xCT system, deplete GSH, impair GPX4, and promote lipid peroxidation. Dysregulated iron metabolism (TFR1, ferritin) drives Fenton reactions. p53 suppresses SLC7A11 and promotes iron accumulation via FDXR, whereas AhR signaling modulates ferroptosis via SLC7A11. This figure was created in BioRender. tonu, r. (2025) (https://BioRender.com/0q9bi92). AIF apoptosis-inducing factor, DISC death-inducing signaling complex, FADD Fas-associated death domain protein, TRADD TNF receptor-associated death domain protein, DRAM damage-regulated autophagy modulator, CaMKII calcium/calmodulin-dependent protein kinase II, MLKL mixed lineage kinase domain-like pseudokinase, GLUD1 glutamate dehydrogenase 1, FDXR ferredoxin reductase

Moderate levels of ROS, on the other hand, inhibit apoptosis by activating prosurvival pathways such as the PI3K/AKT, ERK, and NF-κB pathways. The PI3K/AKT pathway inactivates BAD and BAX while also suppressing caspase-9 and caspase-3 activation.^170^ Similarly, ERK signaling phosphorylates and neutralizes BAD, BIM, and BMF (Ser77), preventing their interaction with BCL-2 family proteins and enhancing the expression of BCL-2 and BCL-XL.^171^ Additionally, NF-κB upregulates antiapoptotic genes, including FLIP, BCL-XL, c-IAP, XIAP, TRAF1, and TRAF2, further blocking cell death^172^ (Fig. 6a).

#### Autophagy

Autophagy is a tightly regulated cellular degradation process in which lysosomes eliminate protein aggregates, damaged organelles, and invading pathogens.^173^ ATG4 uniquely regulates autophagy by processing and deconjugating LC3/ATG8-PE. ROS-induced ATG4 oxidation enhances LC3 lipidation and early autophagosome formation,^174^ although phagophore growth can occur independently of LC3/ATG8.^162^ ROS also regulate autophagy indirectly through the activation of MAPKs, including JNK and p38.^175^ JNK phosphorylates c-Jun, enhancing transcription of Beclin 1, crucial for autophagosome formation. and regulates LC3, facilitating autophagy.^176^ p38β MAPK induces autophagy by phosphorylating ULK1 at Ser555, particularly in response to cancer-related stimuli.^177^ The p53 pathway induces autophagy by upregulating Beclin 1 and damage-regulated autophagy modulator (DRAM).^178^ ROS-Ca^2+^ signaling also facilitates autophagy via calcium-permeable ion channels such as those of the transient receptor potential (TRP) family.^179^ TRPV1 mediates prosurvival autophagy in thymocytes in response to capsaicin,^180^ whereas TRPC1-driven Ca^2+^ entry triggers autophagy under hypoxia and nutrient deprivation.^181^ Additionally, TRPM2 induces Beclin 1 phosphorylation at Ser90 via CaMKII, increasing hepatocyte vulnerability to cell death during oxidative stress.^181^ Besides, In response to oxidative DNA damage, PARP1 activates AMPK, which in turn suppresses mTOR signaling—a key inhibitor of autophagy.^5^ This pathway induces a prosurvival form of autophagy in NPC cells (Fig. 6b).

Interestingly, autophagy might modulate ROS to promote cancer cell survival and drug resistance, depending on the context. In pancreatic cancer, autophagy mitigates Tetrandrine-induced ROS, preserving mitochondria and reducing apoptosis, thereby contributing to drug resistance.^182^ HIF1α-BNIP3-mediated mitophagy reduces mitochondrial ROS and NLRP3 inflammasome activation, which is protective in conditions like renal fibrosis but potentially promotes tumor progression.^183^ Therefore, context-based autophagy targeting could be a potential antineoplastic therapeutic strategy.

#### Necroptosis

ROS amplify RIPK1 activation via a feedback loop. TNF-α-induced RIPK3 activation generates ROS, which promote RIPK1 autophosphorylation at Ser161 and facilitate the formation of disulfide bond-linked aggregates through cysteines 257, 268, and 586.^184^ This enhances RIPK1 recruitment of RIPK3, further increasing ROS production and driving necrosome formation. Additionally, ROS oxidize RIPK1 and reduce the levels and activation of executioner caspases-3, -6, and -7, priming the hyperglycemic shift from apoptosis to necroptosis before TNF-α engages death receptors.^185^ Besides, ROS-driven JAK/STAT signaling upregulates Z-DNA binding protein 1 (ZBP1), which forms a complex with RIPK3 in the absence of RIPK1, triggering MLKL-mediated necroptosis.^64^ Notably, RIPK3 links necroptotic signaling to cellular metabolism by interacting with glutamate dehydrogenase 1 (GLUD1), which catalyzes the conversion of glutamate to alpha-ketoglutarate.^186^ Interestingly, insulin-induced ROS might induce necrosis by initiating PI3K/AKT, a prosurvival pathway,^187^ suggesting context-specific ROS modulation of necroptosis (Fig. 6b).

#### Ferroptosis

This process begins with the inhibition of the cysteine/glutamate antiporter system xCT, which is composed of solute carrier family 7 member 11 (SLC7A11) and solute carrier family 3 member 2 (SLC3A2) and limits the extracellular cysteine uptake required for GSH synthesis.^188^ Depletion of GSH impairs GPX4, halting the detoxification of lipid hydroperoxides and triggering unchecked lipid peroxidation^189^. Lipid peroxides react with ferrous iron (Fe^2+^) through Fenton reactions, generating ROS that damage cellular membranes.^190^ Dysregulated iron metabolism, characterized by increased transferrin receptor 1 (TFR1) expression and decreased ferritin levels, leads to iron overload and further amplifies ROS production.^190^ Antioxidant systems, including ferroptosis suppressor protein 1 (FSP1)-ubiquinol (CoQH2)-vitamin K hydroquinone (VKH2), dihydroorotate dehydrogenase (DHODH)-CoQH2, and GTP cyclohydrolase 1 (GCH1)-tetrahydrobiopterin (BH4), provide protection by mitigating oxidative damage and inhibiting ferroptosis.^188^

Under excessive ROS, p53 plays a crucial role in promoting ferroptosis by negatively regulating SLC7A11, leading to decreased GSH and increased sensitivity to ferroptosis by limiting the antioxidant capacity of cells.^191^ Additionally, p53 influences iron homeostasis through enzymes like ferredoxin reductase (FDXR), enhancing iron accumulation, which is pivotal for ferroptosis.^192^ Paradoxically, ROS can modulate ferroptosis by regulating SLC7A11 expression through the Nrf2-mediated pathway.^193^ Interestingly, a recent study identified a GPX4- and FSP1-independent regulation of ferroptosis involving the phospholipid-modifying enzymes MBOAT1 and MBOAT2, which are regulated by sex hormones.^194^ Ferroptosis may induce immunosuppression, as ferroptosis in MDSCs results in the release of oxygenated lipids that impair T-cell function (Fig. 6b).

#### Disulfidptosis

**Fig. 7:**
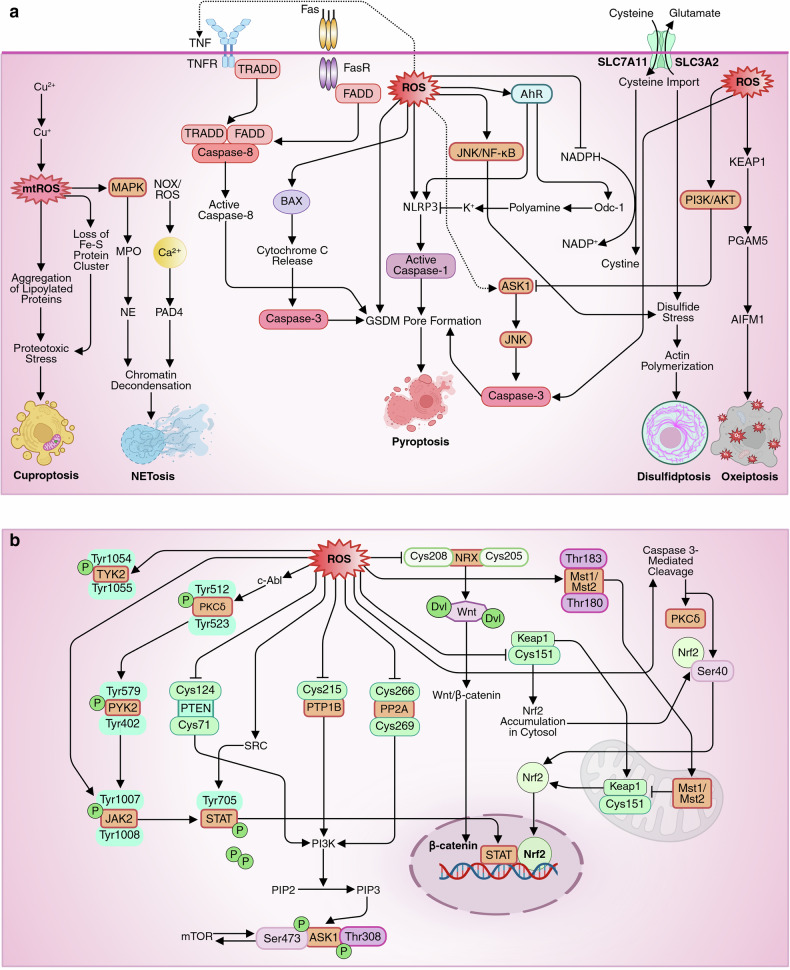
**a** The mechanism of ROS-mediated induction of disulfidptosis, pyroptosis, oxeiptosis, NETosis, and cuproptosis. In disulfidptosis, glucose starvation in SLC7A11-overexpressing cells causes ROS-mediated NADPH depletion, impairing cysteine-to-cystine conversion, resulting in abnormal disulfide bond formation in cytoskeletal proteins and structural collapse. ROS-induced JNK and NF-κB signaling may further regulate disulfidptosis. In pyroptosis, ROS activate the NLRP3 inflammasome via TXNIP dissociation, leading to caspase-1-mediated cleavage of gasdermin (GSDM) pore formation and the release of proinflammatory cytokines. ROS also oxidize mitochondrial membranes and activate the JNK-caspase pathway, enhancing pyroptosis or, conversely, inhibiting it through PI3K/AKT-mTORC2 signaling. Oxeiptosis is triggered when ROS oxidize KEAP1, releasing PGAM5, which dephosphorylates AIFM1 to initiate this caspase-independent cell death that selectively eliminates ROS-damaged cells. In NETosis, ROS generated by NOX or mitochondria activate MAPKs and PAD4, promoting chromatin decondensation and the release of neutrophil extracellular traps (NETs), which contribute to chronic inflammation and cancer progression. Finally, in cuproptosis, ROS exacerbate oxidative stress through copper redox cycling, causing toxic hydroxyl radical production, aggregation of lipoylated TCA cycle proteins, and mitochondrial dysfunction. **b** Mechanisms of ROS-mediated modulation of Wnt/β-catenin, PI3K/AKT, Nrf2, and JAK/STAT signaling pathways. In PI3K/AKT signaling, ROS oxidize cysteine residues in phosphatases such as PTEN, PTP1B, SHP2, and PP2A, inhibiting their activity and sustaining AKT activation. In Wnt signaling, ROS oxidatively inactivate nucleoredoxin (NRX), releasing its suppression of disheveled (Dvl) and enabling Wnt/β-catenin pathway activation. ROS also trigger Nrf2 signaling by directly oxidizing Keap1 or through Mst1/Mst2 autophosphorylation, which inhibits Keap1; Nrf2 is then phosphorylated at Ser40 by PKCδ to become transcriptionally active. In JAK/STAT signaling, ROS initiate the phosphorylation of JAK2 and TYK2, promoting inflammation and tumor progression. Additionally, ROS activate c-Abl in vascular smooth muscle cells, which phosphorylates PKCδ and subsequently PYK2, leading to JAK2 transphosphorylation. Oxidation of Cys215 in PTP1B inhibits its phosphatase activity, prolonging STAT activation and amplifying oncogenic signaling. This figure was created in BioRender. tonu, r. (2025) (https://BioRender.com/0q9bi92). NLRP3 NLR family pyrin domain containing 3, TXNIP thioredoxin-interacting protein, PGAM5 phosphoglycerate mutase family member 5, AIFM1 apoptosis-inducing factor mitochondria-associated 1, PAD4 protein arginine deiminase 4, PTEN phosphatase and tensin homolog, PTP1B protein tyrosine phosphatase 1B, PP2A protein phosphatase 2A, PKCδ protein kinase C delta, PYK2 proline-rich tyrosine kinase 2

Disulfidptosis is a form of PCD induced by glucose starvation in SLC7A11-overexpressing cells, where reduced NADPH production impairs cysteine-to-cystine conversion, causing cysteine accumulation and disulfide stress.^195^ Excessive OH• depletes NADPH, leading to abnormal disulfide bond formation in actin cytoskeletal proteins, resulting in F-actin aggregation, cytoskeletal contraction, detachment from the plasma membrane, and structural disruption, ultimately culminating in cell death.^196^ Besides, ROS might induce disulfidptosis by regulating the JNK and NF-κB pathways.^197,198^ Recent studies have shown that cells with high disulfidptosis scores are more vulnerable to glucose deprivation^199^. This metabolic vulnerability can enhance sensitivity to certain anticancer drugs, although in some contexts, disulfidptosis induction might paradoxically contribute to drug resistance in cultured cells through adaptive stress responses^199^ (Fig. 7a).

#### Pyroptosis

ROS play dual roles in pyroptosis, which is mediated by inflammasomes and gasdermin proteins (GSDMs).^200,201^ Under ROS stress, thioredoxin-interacting protein (TXNIP) dissociates from TRX and activates NLR family pyrin domain containing 3 (NLRP3) inflammasomes, leading to caspase-1 activation and GSDMD cleavage, forming membrane pores and releasing proinflammatory cytokines.^202,203^ Mito-ROS oxidize GSDMD cysteine residues, enhancing its cleavage by caspase-1/caspase-11.^204^ Interestingly, NLRP3 and GSDM pore formation in the mitochondrial membrane can induce ROS production by causing mitochondrial dysfunction.^205^ Iron-induced ROS recruit BAX to mitochondria via translocase of outer mitochondrial membrane 20 (TOMM20), promoting cytochrome C release, caspase-3 activation, GSDME cleavage and pyroptosis.^206^ ROS-induced TNF signaling can also drive RIPK/FADD-mediated caspase-8 activation to cleave GSDMD or GSDMC and induce pyroptosis.^207^ ROS elevate intracellular Ca^2+^ levels, triggering ER stress, Caspase-3 activation, and GSDME-mediated pyroptosis in TNBC cells.^208^ Additionally, ROS can trigger the ASK1-JNK-caspase-3-GSDME pathway to promote pyroptosis.^209^ Interestingly, ROS can inhibit pyroptosis through the PI3K/AKT-mTORC2 pathway, suppressing ASK1 activation. ROS-p53 signaling transcriptionally upregulates GSDME and caspases, switching apoptosis to pyroptosis.^210^ ROS stress can activate NF-κB, which induces the expression of inflammasome-associated genes. Additionally, ROS-induced AhR signaling can either activate the NLRP3 inflammasome,^211^ or inhibit macrophage pyroptosis by upregulating ornithine decarboxylase 1 (Odc-1), enhancing polyamine biosynthesis (e.g., spermine), which suppresses K^+^ efflux and inflammasome assembly, as observed in ulcerative colitis patients^212^ (Fig. 7a).

However, acute NLRP3 activation induces tumor-suppressive pyroptosis and antitumor immunity, whereas chronic activation promotes metastasis through IL-1β/IL-18-driven inflammation.^213^ Notably, GSDME expression enhances tumor cell phagocytosis by tumor-associated macrophages and boosts the activity of tumor-infiltrating natural killer cells and CD8^+^ T lymphocytes, amplifying the antitumor immune response.^214^

#### Oxieptosis

ROS oxidize specific cysteine residues (e.g., Cys151, Cys273, and Cys288) on KEAP1, which acts as a cellular ROS sensor.^215^ At low to moderate ROS levels, KEAP1 releases Nrf2, while at excessive ROS levels, KEAP1 releases PGAM5, a mitochondrial serine‒threonine phosphatase that dephosphorylates AIFM1 at Ser116 to activate the oxeiptosis pathway.^216^ This caspase-independent cell death process selectively removes ROS-damaged cells, preventing inflammation and preserving tissue homeostasis (Fig. 7a).

#### NETosis

ROS induce NETosis through both NOX-dependent and NOX-independent mechanisms. In NOX-dependent NETosis, NOX generates ROS, activating the MAPKs-ERK, p38 MAPK, and JNK pathways.^217^ This promotes the migration of myeloperoxidase (MPO) and neutrophil elastase (NE) to the nuclear envelope, where NE partially degrades histones, leading to chromatin decondensation and the release of neutrophil extracellular traps (NETs).^6^ In NOX-independent NETosis, ROS are produced by mitochondria and are triggered by stimuli such as calcium ionophores or ultraviolet light.^218^ Elevated calcium activates peptidyl arginine deiminase 4 (PAD4), causing histone citrullination, chromatin decondensation, and NET formation.^6^ Both pathways highlight ROS as key initiators of NETosis.

NETosis promotes chronic inflammation and cancer progression by inducing DNA damage, proliferation, and angiogenesis via MMP9 and VEGF.^219^ It facilitates metastasis via IL-17/granulocyte colony-stimulating factor (G-CSF) signaling, high mobility group box 1 (HMGB1) activation, and carcinoembryonic antigen-related cell adhesion molecule 1 (CEACAM1)-mediated adhesion.^220^ NETosis shifts neutrophils to a protumor N2 phenotype, promoting an immunosuppressive TME, EMT, and chemoresistance.^221^ NETs shield tumor cells from immune detection, driving immunotherapy resistance via PD-L1 expression, T-cell exhaustion, and IL-8-mediated immunosuppression, while NETosis in the hypoxic TME enhances chromatin decondensation, tumor cell trapping, and immune evasion, making NETs a critical therapeutic target^221^ (Fig. 7a).

#### Cuproptosis

ROS induce cuproptosis through Fenton-like reactions, where copper cycles between Cu^+^ and Cu^2+^, generating toxic OH• that damages biomolecule and disrupts mitochondrial function.^222^ This oxidative stress promotes copper accumulation, leading to the aggregation of lipoylated TCA cycle proteins and the loss of iron‒sulfur cluster proteins—key hallmarks of cuproptosis. In cancer, this mechanism exploits the heightened copper demand and metabolic vulnerabilities of tumor cells, particularly those reliant on OXPHOS.^222^ By selectively targeting cancer cells with copper ionophores like Elesclomol or ROS-inducing agents, including radiotherapy or combination therapy, cuproptosis can be triggered, offering a promising strategy to overcome cancer.^223,224^ However, balancing copper toxicity and therapeutic efficacy remains critical to minimize off-target effects while maximizing anticancer outcomes (Fig. 7a).

## ROS selectivity in signaling pathway modulation

ROS activate various signaling pathways through selective interactions dictated by the chemistry of specific ROS and the biochemical properties of target molecules. O_2_•⁻ reacts selectively, reducing transition metals and forming ONOO⁻ with nitric oxide, while also damaging Fe–S cluster enzymes critical to metabolism.^11,225^ H_2_O_2_ is less reactive but selectively oxidizes cysteine residues with low pKa values and reacts with transition metals to generate OH•.^12^ OH• is highly indiscriminate, reacts at near diffusion-controlled rates and drives oxidative damage.^226^ ONOO⁻ reacts with thiols, metals, and CO₂, generating oxidants such as NO_2_• and CO_3_•⁻.^225^ ¹O_2_ is a nonradical but highly reactive species, central to photobiological and oxidative processes.^11^

The redox potential of specific residues, particularly cysteine, plays a critical role in ROS selectivity. Cysteine residues with low pKa values (e.g., 4.7–5.4) are more nucleophilic and prone to oxidation. For instance, the catalytic cysteine in PTPs has a low pKa because of the Cys-X5-Arg motif, making them highly susceptible to oxidation by H_2_O_2_, leading to the formation of sulfenic acid (SOH), disulfide bonds (S‒S), or sulfenyl-amide (S‒N) bonds.^227^ Proteins containing redox-sensitive motifs, such as the CXXC motif in nucleoredoxin (NRX) or TRX, are preferential targets for ROS.^228^ These motifs are structurally designed to sense and respond to redox changes. For example, the oxidation of Cys205 and Cys208 in NRX disrupts its interaction with disheveled (Dvl), stabilizing β-catenin and activating the Wnt/β-catenin pathway. The sensitivity of cysteine residues to redox changes is influenced by their steric accessibility and the pKa value of their thiol groups. Typically, the pKa value of free cysteine is approximately 8.2. However, when cysteine residues are located near positively charged residues, their pKa decreases to less than 6.5, increasing their susceptibility to oxidation.^229^ This shift in pKa is associated with the transformation of redox-sensitive thiols into potent nucleophiles in the presence of basic residues. Cellular redox state might modulate the sensitivity of specific molecules to ROS and subsequent reactions. For example, reducing conditions strengthen some interactions (e.g., NRX-Dvl), and oxidizing conditions weaken them in modulating the Wnt/β-catenin pathway.^230^ Proteins such as TRX and glutaredoxin (GRX) might modulate ROS interactions by reducing oxidized cysteine residues, thereby restoring protein function. For example, TRX reduces oxidized ASK1, preventing its activation, whereas GRX regulates the redox state of proteins like Ras, influencing MAPK signaling.^58^

## Mechanism of the ROS-mediated modulation of signaling pathways

### PI3K/AKT/mTOR pathway

ROS enhance PI3K/AKT signaling by suppressing PTEN through H_2_O_2_-induced oxidation (Cys124/Cys71) or Ser380 phosphorylation, inhibiting membrane binding,^231^ forming disulfide bridges and promoting migratory shifts.^232^ Interestingly, PTEN degradation also occurs independently of H_2_O_2_, as observed in pancreatic cancer cells with elevated prostaglandin production via 5-LOX and COX-2 overexpression.^232,233^ H_2_O_2_ also oxidizes cysteines in PTPs, enabling receptor tyrosine kinase (RTK) phosphorylation,^234^ Insulin-induced ROS inhibit PTP1B, preventing insulin pathway dephosphorylation and promoting PI3K/AKT signaling.^235^ H_2_O_2_ also increases threonine phosphorylation in Caco-2 cells by downregulating PP2A via oxidation of its redox-sensitive Cys266 and Cys269, forming inhibitory disulfide bonds,^236^ particularly through oxidation of the B55α isoform.^237^ Notably, some studies suggest that ROS-mediated inhibition of the PI3K pathway regulates cell death and enhances chemotherapy sensitivity in cancers, although the exact mechanisms are unclear^238,239^ (Table 1, Fig. 7b).

**Table 1:** List of signaling pathways mediated by ROS with mechanistic highlights

Signaling pathway	Role in cancer signaling	Signaling intermediate	Site	Modification	Mechanistic aspect	Refs.
PI3K/AKT	Drug resistance, Cancer cell proliferation, Metastasis	PTEN	Cys124 Cys71	-S-S-	By inhibiting all these phosphatases, ROS activate PI3K/AKT signaling.	^231^
		PTP1B	Cys215	S-N-R		^235^
		PP2A	Cys266 Cys269	-S-S-		^236^
AhR	Cell survival and proliferation, EMT, Cell death	2-Oxindole, IDO1	–	–	ROS initiate 2-Oxindole, which in turn activates IDO1. Activated IDO1 then catalyzes the Trp transformation into Kyn. Upon interaction with AhR, Kyn promotes activation and nuclear translocation of AhR.	^258,259^
		Tryptophan	–	FICZ	ROS induce photo-transformation of Trp into FICZ, which in turn activates and transfers AhR to the nucleus.	^257^
EGFR	Tumor cell proliferation, Drug resistance	NA	Tyr845	Phosphorylation	ROS directly activate EGFR by phosphorylation on its Tyr845 residue.	^280^
		NA	Cys797	R–S–OH	ROS activate EGFR by oxidative modification of Cys797 residue on EGFR.	^281^
		PTEN	Cys124, Cys71	-S-S-	ROS inactivate phosphatase like PTEN by oxidizing Cys124 and Cys71, thereby activating EGFR.	^231^
TGF-β	Drug Resistance, Cell proliferation, Autophagy	LAP-β	Met253	Conformational Change	ROS induce Met-oxidation of LAP-β, triggering a structural change that results in the initiation of TGF-β.	^249^
		MMP2 & 9	Cys73	R–S–OH	ROS oxidize the thiol residues in MMPs into sulfenic acid, leading to their activation. Activated MMPs then oxidize LAP-β to release TGF-β. Cys73 was suggested to be associated with the MMP regulation.	^250^
Wnt/β-catenin	Tumor progression, Migration, Metastasis	NRX	Cys205 Cys208	-S-S-	ROS induce oxidation followed by inactivation of NRX that interacts with Dvl and decline their inhibitory activity on the destruction complex. NRX intervention leads to interaction between Dvl and Wnt and activates Wnt/β-catenin signaling.	^71^
HIF1α	Cell proliferation, Angiogenesis, Metastasis, Apoptosis	PHD	Fe^2+^	Fe^3+^	H_2_O_2_ inhibit the binding of ferrous iron to prolyl hydroxylases (PHDs) and upregulation of PHDs. Inhibition of PHDs results in initiation of HIF1α.	^251^
		Succinate	–	–	Under Hypoxia, SDH inverse catalysis induced succinate accumulation inhibits PHD activity by impeding the decarboxylation of α-ketoglutarate to succinate, a co-reaction required for HIF1α hydroxylation, thereby raising HIF1α expression. Though ROS association with succinate level is ill-defined, simultaneous re-oxidization of accumulated succinate by SDH produces mtROS.	^252^
		NA	Cys520/Cys533	S-nitrosylation	ROS directly induce HIF1α through oxidative alteration of its Cys520/(Cys533 in mouse).	^253^
		AMPK, ATM, PI3K/Akt, and MAPK	See AMPK, ATM, PI3K/Akt, and MAPK	See AMPK, ATM, PI3K/Akt, and MAPK signaling	ROS may activate HIF1α through AMPK, ATM, PI3KAkt, and MAPK-induced phosphorylation of different residues of the HIF1α transactivation domain.	^254,255^
Nrf2	Cancer cell survival, Drug resistance	Keap1	Cys151	-S-S-	ROS induce Cys-oxidative modification and inactivation of Keap1, an inhibitory protein of Nrf2. The accumulated cytoplasmic Nrf2 then undergoes phosphorylation on Ser40 by caspase-3 initiated PKCδ and gets activated.	^59^
		Mst1/Mst2	Thr183 Thr180	Phosphorylation	Under oxidative stress, Mst1/Mst2 gets autophosphorylated on Thr180 and Thr183 residues. Activated Mst1/2 then inhibits Keap1, thereby activating Nrf2.	^242^
JAK/STAT	Inflammation, Angiogenesis, Cancer, Metastasis	JAK2	Tyr1007 Tyr1008	Phosphorylation	ROS can directly activate JAK2 through tyrosine phosphorylation, which in turn activates the STAT factor.	^77^
		TYK2	Tyr1054 Tyr1055	Phosphorylation	ROS phosphorylate TYK2 on its tyrosine residues and initiates TYK2, which then potentiates STAT signaling.	^77^
		PTP1B	Cys215	S-N-R	By inhibiting STAT repressor protein, PTP1B through Cys-oxidation, ROS initiate JAK/STAT signaling.	^248^
		SRC	Cys185 Cys277	Cys-SOH	ROS activate SRC kinase through Cys-oxidative modification, thereby accelerating JAK/STAT signaling.	^247^
		PKCδ	Tyr512 Tyr523	Phosphorylation	ROS activate JAK through PKCδ phosphorylation that, in turn, phosphorylates PYK2, which then transphosphorylates JAK. Activated JAK then switches on the STAT factor.	^245^
ERK1/2	Drug resistance, Metastasis	EGFR	Tyr845	Phosphorylation	ROS activate EGFR by Tyr phosphorylation or Cys oxidation. Activated EGFR then modulates SH2- Grb2-SOS, where SOS initiates Ras by catalyzing the GTP binding to Ras. Ras then initiates Raf, which in turn potentiates MEK1/2 that activates ERK1/2.	^280^
			Cys797	R–S–OH		^281^
		SRC Kinase	Cys185 and Cys277	Cys-SOH	ROS activate SRC kinase through oxidative modification of cysteine residues. Activated SRC kinases then initiate Raf-1 directly through phosphorylation of their Tyr340/341 residue or EGFR mediated activation, leading to subsequent activation of ERK1/2.	^247^
		Ras	Cys118	R–S–OH	ROS activate ERK in Ras-dependent manner (by activating Ras through Cys118 oxidation), which is independent of EGFR initiation.	^281^
		DUSP-3	Cys124	R–S–OH	ROS inhibit the ERK inhibitory phosphatase, DUSP3, through Cys124 oxidation, resulting in ERK activation.	^287^
ASK1	Autophagy	TRX	Cys32, Cys35	-S-S-	ROS degrade TRX, an ASK1 inhibitory protein, through Cys oxidation, resulting in its dissociation from ASK1.	^289,291^
		ASK1/2	Thr838. Thr806	Oligomerization and Phosphorylation	ROS induce homo-oligomerization of ASK1, followed by autophosphorylation at Thr838, while hetero-oligomerization involves the autophosphorylation of ASK2 at Thr806. Subsequent phosphorylation of Thr838 residue of ASK1 by ASK2.	^291^
JNK	Autophagy	MAPKKKs (MEKK1/2/3/4, ASK-1 and MLK)	–	Phosphorylation	ROS activate MAPKKKs through phosphorylation, which in turn activate MAPKKs like MKK3/4/6/7 through phosphorylation of Ser/Thr residues. Activated MEKs switch on JNK through phosphorylation of its critical TPY motif.	^288^
		ASK1	–	–	ROS activate ASK1 directly through dimerization and subsequent phosphorylation or indirectly through TRX inhibition. Activated ASK1 then initiates JNK signaling.	^291^
		GSTPs	Arg70, Arg74, Asp90, Asp94, Thr67	Dimerization	GSTP that tether JNK to repress JNK are oligomerized by ROS, thereby activating JNK.	^290^
P38	Autophagy, Inhibition of drug resistance	MAPKKKs (ASK1, MEKK1/2/3/4 and MLK3)	–	Phosphorylation	ROS phosphorylate and activate MAPKKKs, which in turn activates MAPKKs like MEK3/6. Activated MEKs induce dual phosphorylation of p38 on its critical threonine and tyrosine residues.	^288^
		p38α	Cys119, Cys162	-S-S-	ROS phosphorylate both the p38α and MKK3 and mediates heterodimerization (forming a disulfide bond) between Cys104 of MKK3 and Cys119 and Cys162 of p38α, leading to p38α overexpression.	^292^
PARP1	Cell death signaling	PARP1	Leu698 and Leu701	Relieving autoinhibition	Upon ROS-induced DNA damage, PARP1 HD autoinhibition domain is unfolded by redistributing Leu698 and Leu701 out of the HD interior, thereby promoting the DNA binding of PARP1 and subsequent auto PARylation.	^293^
		HPF1	–	–	Under oxidative DNA damage, HPF1-PARP1 binding mediates ADP-ribosylation of serine residues within the histone and PARP1. Besides, HPF1 remodels the active site of PARP1 by forming Arg239-Glu284 that jointly act as an active site for ser-ADP-ribosylation.	^294,296^
ATM	DNA damage repair, Autophagy	PRDX2, TRX1	Cys2991 of ATM	-S-S-	In the presence of ROS, PRDX2, and TRXR1 oxidize the Cys2991 residue on ATM, which then undergoes autophosporylation at Ser1981 residue, leading to complete activation of ATM.	^297^
P53	Apoptosis, Cell cycle blockage, Genome maintenance	ATM	Cys2991	-S-S-	ROS-activated ATM initiates p53 on Ser15 and simultaneously activates CHK2, which then potentiates p53 by phosphorylating their Ser20 residue.	^297^
		JNK	See (JNK)	See JNK	Upon ROS-mediated activation, JNK phosphorylates p53 on Ser20 and Thr81, resulting in p53 activation.	^300^
		P38	See (p38)	See p38	ROS-induced p38 MAPK induces phosphorylation of p53 on Ser15, 33, 37 and 46, leading to p53 initiation.	^299^
		ASK1	See ASK1	See ASK1	ROS induce transactivation of p53 in ASK1- JNK/p38 dependent way.	^291^
AMPK	Tumor suppression	AMP/ADP	Thr172 of AMPK	Phosphorylation	Under oxidative stress, a decrease in ATP level increases AMP/ADP level and promotes AMP/AMPK-γ subunit binding, resulting in increased ROS mediated Thr172 of AMPK-α subunit phosphorylation followed by AMPK activation.	^302^
		Ca^2+^	Thr172 of AMPK	Phosphorylation	Hypoxia-induced ROS promote calcium levels, which involves the initiation of CaMKKβ that induces phosphorylation of AMPK Thr172 residue, thereby activating AMPK potentiation.	^302^
		NA	Cys299 Cys304	-S-S-	ROS directly induce Cys oxidation of AMPK and potentiates AMPK.	^303^
NF-κB	Pro-tumorigenic signaling, Drug resistance, Tumor progression, Cell death	IKKβ	Cys179	S-glutathionylation	Excessive ROS oxidize the Cys179 residue on IKKβ to inhibit the activation of IKK, thereby inhibiting NF-κB activation, which results in declined pro-cancer signaling.	^269^
		NA	Cys62	S-glutathionylation	A high level of ROS directly oxidize Cys62 of NF-κB, thereby reducing the DNA binding capacity of NF-κB.	^264^
		IKKβ	Ser177 Ser181	Phosphorylation	TNF-α induced ROS oxidize LC8 to promote degradation of IκB on its Ser32 and Ser36 residues by activated IKK. Degradation of IKβ upregulates the initiation as well as nuclear relocation of NF-κB.	^262^
		IKKα	Ser176 Ser180	Phosphorylation	IL-1β induced ROS activate NIK, which phosphorylates IKKα on its serine residue. Activated IKKα induces phosphorylation and ubiquitination on several serine residues of p100 to generate the NF-κB protein p52.	^270^
		IκBα	Tyr42	Phosphorylation	ROS induce phosphorylation of IκBα, which in turn phosphorylates p65 and promotes its nuclear translocation.	^263^
ATG4	Autophagy	NA	Cys81	R–S–OH	ROS directly oxidize the Cys77 residue of ATG4 into sulfenic acid or both the Cys77 and Cys81, forming a disulfide bond, thus inhibiting ATG4 activity and consequently promoting autophagy.	^272^
			Cys77 Cys81	-S-S-		^272^
			Cys292 Cys361	-S-S-	ROS induce oxidation of Cys292 and Cys361 of ATG4B, inhibiting its autophagy inducing effectivity.	^273^
Ca^2+^	Tumorigenesis, Autophagy	TRPA1	Cys421 Cys621 Cys641 Cys665	-S-S-	ROS activate TRP channels through Cys oxidative modification, except for TRPM2, which involves ADP-ribose tethering to the nudix-box sequence motif (NUDT9-H), and TRPML1, which undergoes Cys palmitoylation. Activated TRP channels then induce calcium ion signaling. While the majority of the TRP channel-mediated Ca^2+^ influx is involved in pro-tumorigenic signaling, the Calcium channel activated by TRPM2 and TRPML1 have autophagic function.	^277^
		TRPV1	Cys616 Cys621	S-nitrosylation		^275^
			Cys258 Cys742	-S-S-		
		TRPM2	Glu829 Arg845	NAD production		^278^
		TRPC5	Cys553 Cys558	S-nitrosylation		^276^
		MCOLN1/ TRPML1	Cys565 Cys566 Cys567	Palmitoylation		^279^

*ROS* Reactive oxygen species, *PI3K* Phosphoinositide 3-kinase, *AKT* Protein kinase B, *Wnt* Wingless and int, *Nrf2* Nuclear factor erythroid 2–related factor 2, *PTEN* Phosphatase and Tensin homolog, *PTP1B* Protein tyrosine phosphatase 1B, *PP2A* Protein phosphatase 2A, *Keap1* Kelch like-ECH-associated protein 1, *Mst1/Mst2* macrophage-stimulating 1/2, *PKCδ* Protein kinase C δ, *JAK* Janus kinase, *STAT* Signal transducers and activators of transcription, *TYK* Tyrosine Kinases, *Tyr* Tyrosine, *PYK2* Proline-rich tyrosine kinase 2, *IDO1* Indoleamine-2,3-dioxygenase-1, *Trp* Tryptophan, *Kyn* kynurenine, *FICZ* 6-formylindolo[3,2-b]carbazole, *AHR* Aryl hydrocarbon receptor, *TGF-β* Transforming Growth Factor-β, *LAP* Latency-associated peptide, *MMP* Matrix metalloproteinase, *Met* Methionine, *Cys* Cysteine, *PHDs* Prolyl hydroxylases, *SDH* Succinate dehydrogenase, *AMPK* AMP-activated protein kinase, *ATM* Ataxia telangiectasia mutated, *MAPK* Mitogen-activated protein kinase, *IκB* Inhibitory κB, *IKK* IκB kinases, *NF-κB* Nuclear Factor kappa B, *ATG4* Autophagy-related 4, *TNFα* Tumor Necrosis Factor α, *IL-1β* Interleukin-1β, *TRPV1* Transient Receptor Potential Vanilloid 1, *TRPC5* Transient Receptor Potential Channel 5, *TRPA1* Transient receptor potential ankyrin 1, *TRPM2* Transient Receptor Potential Melastatin 2, *TRPML1* Transient Receptor Potential Mucolipin 1, *EGFR* Epidermal growth factor Receptor, *ERK1/2* Extracellular-regulated kinase 1/2, *ASK1* Apoptosis Signal-regulating Kinase 1, *JNK* c-Jun N-terminal Kinase, *SRC* kinase Proto-oncogene tyrosine-protein kinase, *DUSP3* Dual specificity protein phosphatase 3, *MAPKKK* Mitogen Activated Protein (MAP) kinase kinase kinase, *MKK* Mitogen-activated protein kinase kinase, *MEK* Mitogen-activated protein kinase, *Tyr* Tyrosine, *Cys* Cysteine, *Ser* Serine, *Thr* Threonine, *ATM* Ataxia-telangiectasia mutated, *CHK2* Checkpoint kinase 2, *AMP* Adenosine monophosphate, *ADP* Adenosine diphosphate, *ATP* Adenosine triphosphate, *AMPK* AMP-activated protein kinase, *MAPK* Mitogen Activated Protein kinase, *CaMKKβ* Calmodulin-dependent protein kinase β, *PARP* Poly (ADP-ribose) polymerase, *DNA* Deoxyribonucleic acid, *Arg* arginine, *Glu* Glutamic acid, *HPF1* Histone PARylation factor 1, *HD* Helical Domain

### Wnt/β-catenin pathway

NOX1 generates H_2_O_2_, which oxidizes the Cys205 and Cys208 residues of NRX.^71^ This oxidation inactivates NRX, which normally binds to disheveled (Dvl) and represses its ability to inhibit the β-catenin destruction complex, thereby enabling it to stabilize β-catenin, facilitating ROS-driven Wnt/β-catenin signaling.^71^ Thus, a redox-dependent interaction between NRX and Dvl is essential for Wnt/β-catenin signaling modulation by ROS (Table 1, Fig. 7b). Interestingly, NRX can indirectly promote β-catenin signaling by modulating Dvl stability. KLHL12, an E3 ubiquitin ligase adapter, targets Dvl for proteasomal degradation, negatively affecting the canonical Wnt pathway. However, reduced levels of NRX disrupt the interaction between KLHL12 and Dvl, preventing Dvl degradation and preserving the pool of Dvl necessary for sustaining Wnt/β-catenin signaling.^240^

### Nrf2 pathway

Under stress, ROS oxidize Keap1’s cysteine-rich domain (Cys151),^59^ disrupting its interaction with Nrf2 and activating Nrf2. Consequently, Nrf2 levels increase in the cytoplasm, where Caspase-3-cleaved PKCδ further activates Nrf2 via Ser40 phosphorylation, enabling its nuclear translocation to activate ARE expression.^241^ Mitochondrial ROS activate macrophage-stimulating 1/2 (Mst1/2) kinases via autophosphorylation at Thr183/Thr180.^242^ Activated Mst1/2 relocates with Keap1 to mitochondria, phosphorylating its N-terminus, disabling Keap1 dimerization and subsequent Nrf2 activation^243^ (Table 1, Fig. 7b).

### JAK/STAT pathway

ROS, particularly H_2_O_2_, activate JAK2 and TYK2 signaling by phosphorylating JAK2 (Tyr1007/1008) and TYK2 (Tyr1054/1055),^77^ leading to STAT activation via Tyr705 phosphorylation.^244^ In vascular smooth muscle cells, ROS activate c-Abl, which phosphorylates PKCδ (Tyr512/523),^245^ leading to PYK2 phosphorylation (Tyr402/579) and subsequent JAK2 transphosphorylation.^246^ Additionally, ROS activate SRC kinase through Cys-oxidative modification, initiating JAK/STAT signaling.^247^ Besides, ROS oxidize PTP1B, inhibiting its phosphatase activity and thereby preventing dephosphorylation of signaling components such as the IL-4 receptor, which prolongs STAT signaling^248^ (Table 1, Fig. 7b).

### TGF-β pathway

**Fig. 8:**
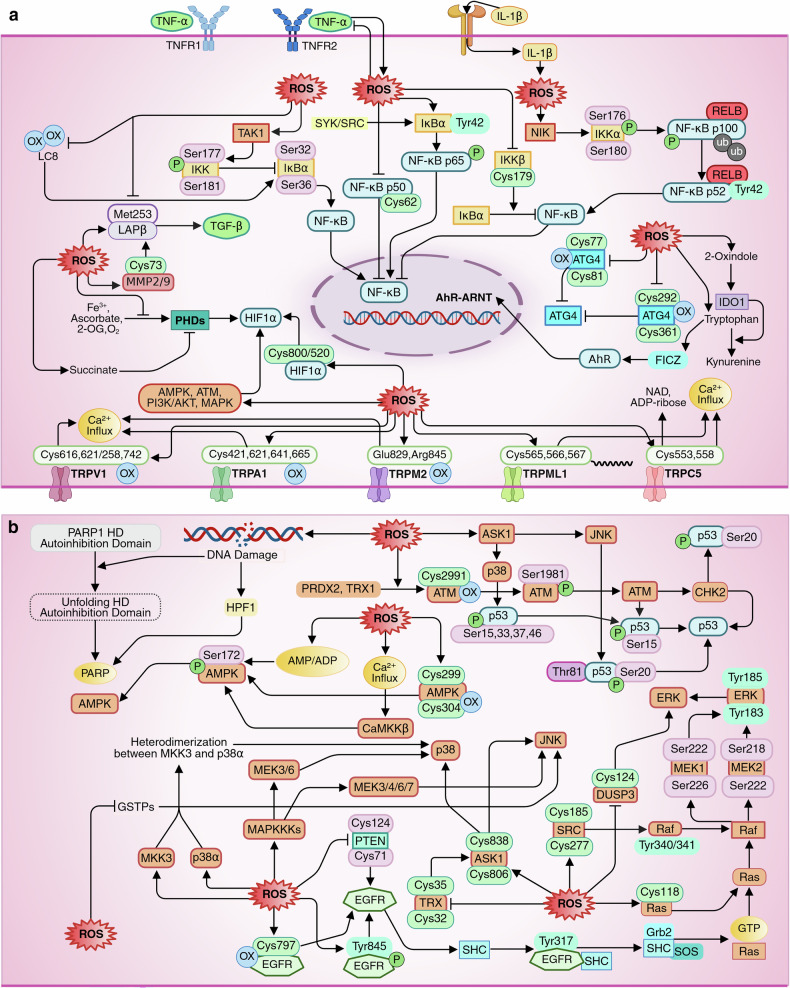
**a** Mechanisms of ROS mediated regulation of AhR, TGF-β, HIF1α, NF-κB, ATG4, and calcium signaling pathways. ROS-derived 2-oxindole activates IDO1, converting tryptophan to kynurenine (Kyn), which binds and activates AhR, while ROS-induced FICZ formation similarly triggers AhR nuclear translocation. In the TGF-β pathway, ROS oxidize methionine residues in LAP-β and activate MMPs, which further release TGF-β by oxidizing LAP-β, with Cys73 involved in MMP regulation. ROS stabilize HIF1α by inhibiting prolyl hydroxylases (PHDs), with succinate accumulation and oxidative modifications at Cys520 enhancing HIF1α levels, along with phosphorylation by AMPK, ATM, PI3K/Akt, and MAPK. NF-κB activation is promoted via IKKβ phosphorylation (Ser177/181) by TAK1, IκBα degradation (Ser32/36), and dynein light chain oxidation, enabling p65 nuclear translocation; however, excessive ROS oxidize NF-κB p50 (Cys62) and IKKβ (Cys179), inhibiting DNA binding and proteasome function, thereby dampening signaling. ROS also activate the noncanonical NF-κB pathway via NIK and IKKα phosphorylation (Ser176/180) and modulate NF-κB via the PI3K/PTEN/Akt axis. In autophagy, ROS oxidize Cys77/Cys81 and Cys292/Cys361 in ATG4/ATG4B, inhibiting their activity and enhancing autophagy. ROS also regulate calcium signaling by oxidizing cysteines in TRP channels (e.g., TRPV1, TRPC5, and TRPA1), promoting Ca^2+^ influx, with TRPA1 activation linked to tumorigenesis. ROS activate TRPM2 via Glu829 and Arg845, generating NAD and ADP-ribose to stimulate Ca^2+^ entry and cell death, and enhance antitumor calcium signaling through palmitoylation of Cys565–567 in MCOLN1 (TRPML1), amplifying ROS-mediated Ca^2+^ responses. **b** Mechanistic action of ROS in modulating EGFR, p53, AMPK, ATM, PARP1, ERK1/2, ASK1, p38, and JNK-dependent pathways. ROS directly activate EGFR via phosphorylation at Tyr845 and oxidation of Cys797, and indirectly by inhibiting PTEN through oxidation of Cys124 and Cys71. EGFR then initiates Ras–Raf–MEK–ERK signaling, with ROS enhancing Ras activation via Cys118 oxidation, activating SRC, and inhibiting DUSP3 via Cys124 oxidation, collectively sustaining ERK1/2 activation. In JNK signaling, ROS degrade TRX, releasing ASK1 through autophosphorylation at Thr838/Thr806, which then activates MAPKKs (MKK3/4/6/7), leading to JNK activation. ROS-induced GSTP oligomerization releases its inhibitory hold on JNK, while oxidative inhibition of MKPs further amplifies JNK signaling. For p38 activation, ROS promote MAPKKK phosphorylation and activate MEK3/6, inducing dual phosphorylation of p38, while also increasing p38α/MKK3 dimerization. ROS activate ATM by oxidizing Cys2991 via PRDX2/TRX1, triggering Ser1981 autophosphorylation; activated ATM phosphorylates p53 at Ser15 and activates CHK2, which phosphorylates p53 at Ser20. JNK and p38 further phosphorylate p53 at Ser20, Thr81, and Ser15/33/37/46, enhancing its tumor-suppressive functions. ROS activate AMPK by increasing AMP/ADP levels, promoting γ subunit binding and Thr172 phosphorylation of the α subunit, with additional activation via CaMKKβ under hypoxia and direct Cys oxidation. Upon ROS-induced DNA damage, PARP1 undergoes conformational changes allowing DNA binding and autoPARylation, while HPF1 reshapes the PARP1 active site to facilitate serine ADP-ribosylation of histones, driving chromatin remodeling and repair. This figure was created in BioRender. tonu, r. (2025) (https://BioRender.com/0q9bi92). IDO1 indoleamine 2,3-dioxygenase 1, MMPs matrix metalloproteinases, IKKβ inhibitor of nuclear factor kappa-B kinase subunit beta, TAK1 TGF-β-activated kinase 1, IκBα NF-kappa-B inhibitor alpha, NIK IKKα inhibitor of nuclear factor kappa-B kinase subunit alpha, TRP transient receptor potential, DUSP3 dual-specificity phosphatase 3, MAPKKs (MKK3/4/6/7) mitogen-activated protein kinase kinases, MEK3/6 MAPK/ERK kinase 3/6, CHK2 checkpoint kinase 2, CaMKKβ calcium/calmodulin-dependent protein kinase kinase beta, HPF1 histone PARylation factor 1

Ionizing radiation-induced ROS activate latent TGF-β1 by oxidizing Met253 in its LAP, triggering conformational changes, suggesting the isoform specificity of ROS in TGF-β activation.^249^ This isoform-specific activation is due to variations in LAP homology (34–38%), despite the high similarity (75%) among TGF-β cytokines. ROS can activate LTGF-β indirectly by potentiating MMP2 and MMP9, which modify thiol residues (e.g., Cys73) to sulfenic acid,^250^ activating MMPs, leading to LAP cleavage and the release of active TGF-β in the ECM (Table 1, Fig. 8a).

### HIF1α pathway

ROS regulate HIF1α by oxidizing Fe^2+^ to Fe^3+^, which inhibits prolyl hydroxylases (PHDs) that degrade HIF1α via hydroxylation.^251^ Notably, PHDs use iron, ascorbate, oxygen, and 2-oxoglutarate to hydroxylate and inhibit HIF1α, while ROS disrupt these interactions, causing HIF1α accumulation. Hypoxia-induced mito-ROS, particularly H_2_O_2,_ can also regulate HIF1α by promoting succinate accumulation (which inhibits PHD expression) via the reverse succinate dehydrogenase (SDH) activity.^252^ Additionally, nitric oxide (NO) induces HIF1α expression by S-nitrosylating Cys800 or Cys520, preventing its binding to the Von Hippel‒Lindau (VHL) protein and proteasomal degradation.^253^ ROS can also indirectly activate HIF1α through pathways such as the AMPK, PI3K/AKT, and MAPK pathways, which phosphorylate HIF1α at specific residues (e.g., Ser419, Ser641, and Ser643)^254,255^ (Table 1, Fig. 8a).

### AhR pathway

ROS, especially H_2_O_2_, activate the AhR pathway by accelerating the production of endogenous ligands like FICZ and kynurenine (Kyn).^256^ Kou et al. reported that H_2_O_2_ increases oxindole levels, a tryptophan catabolite, with its major form, 2-oxindole, effectively activating the AhR pathway.^257^ Besides, H_2_O_2_ and 2-oxindole were observed to promote PD-L1 and indoleamine-2,3-dioxygenase-1 (IDO1), two immune checkpoint proteins.^257^ In glioma cells, IDO1 and TDO were shown to infuse the transformation of Trp into Kyn, followed by AhR activation.^258,259^ Opitz et al. reported that Kyn is an endogenous oncometabolite that has the potential to activate AhR.^258^ Moreover, UV-induced ROS phototransform Trp into FICZ, a potent AhR ligand, highlighting the role of ROS in modulating the tumor milieu via AhR signaling.^257^ (Table 1, Fig. 8a).

### NF-κB pathway

The NF-κB pathway involves five proteins (p50/p105, p52/p100, p65/RelA, RelB, and c-Rel) and operates through two pathways: the canonical pathway, triggered by receptors like TLRs and TNFR, activates p50-RelA via IKKβ and NEMO, while the noncanonical pathway, initiated by BAFF-Rs, activates p52-RelB through NIK and IKKα.^260^ NF-κB is typically inhibited by IκB proteins, which are degraded upon phosphorylation by IKKs, enabling NF-κB activation.

ROS, like H_2_O_2_ and superoxide, act as activators or inhibitors of NF-κB, while NF-κB has pro- or antioxidant effects depending on oxidative stress levels. Superoxide and H_2_O_2_ activate TAK1, which phosphorylates IKKβ at Ser177 and Ser181, enabling IKKβ to degrade IκBα at Ser32 and Ser36, triggering NF-κB signaling.^261^ TNF-α-induced ROS oxidize the dynein light chain (LC8), enabling its binding to IκB and promoting IκBα degradation by IKK, leading to NF-κB activation.^262^ Besides, externally added H_2_O_2_ directly phosphorylates IκBα at Tyr42 via SYK/SRC kinases, which induces the phosphorylation and nuclear translocation of the p65 protein.^263^

Excessive ROS, on the other hand, oxidize the Cys62 residue of NF-κB p50, reducing its DNA binding ability; Notably, this residue is prone to oxidation in the cytoplasm but sensitive to reduction in the nucleus.^264^ Moreover, prolonged and excessive ROS production may inactivate the proteasome, which plays a key role in degrading IκB, an inhibitor of NF-κB.^263,265^ H_2_O_2_ reduces TNF-α-induced IKK activity by oxidizing cysteine residues, impairing IκB degradation and diminishing NF-κB activation.^266^ The same outcome was reported in the case of arsenite^267^ and nitric oxide,^268^ as the Cys179 residue of IKKβ was oxidized,^269^ causing the inhibition of IKK.

In case of noncanonical pathway, IL-1β-induced H_2_O_2_ activates NIK, leading to IKKα phosphorylation (Ser176/Ser180), which converts p100 to p52, initiating NF-κB.^270^ Moreover, H_2_O_2_ was also shown to mediate NF-kB activation in an IKK-dependent way by regulating the PI3K/PTEN/AKT signaling cascade.^271^ However, this interaction remains debated owing to the complexity of the crosstalk between ROS and NF-κB signaling, as well as the variable outcomes in different experimental models (Table 1, Fig. 8a).

### ATG4 pathway

H_2_O_2_ can induce the oxidation of the Cys81 (in the vicinity of the catalytic Cys77) residue of ATG4A and ATG4B into sulfenic acid or through reversible oxidation of Cys77 and Cys81 into disulfide bridges.^272^ However, Zheng et al. identified Cys292 and Cys361 as key sites of ATG4B oxidation function and subsequent autophagy mediation.^273^ Interestingly, Zheng et al. also reported that H_2_O_2_ treatment-induced oxidative modification of the Cys292 and Cys361 residues of ATG4 to restrict LC3 lipidation, leading to autophagy inhibition.^273^ The discrepancy between these studies is due to varied autophagy-inducing systems and levels of ROS^274^ (Table 1, Fig. 8a).

### Calcium pathway

Several studies have reported ROS-induced Cys oxidation and subsequent overexpression of different TRP channels, which in turn activate Ca^2+^. influx. For instance, ROS oxidize the Cys616, Cys621/Cys258 and Cys742 residues of TRPV1;^275^ the Cys553 and Cys558 residues of TRPC5;^276^ and the Cys621, Cys421, Cys641 and Cys665 residues of TRPA1.^277^ H₂O₂ activates TRPM2 by targeting Glu829 and Arg845, producing NAD metabolites such as ADP-ribose, which opens calcium channels and induces cell death.^278^ ROS-mediated Ca^2+^ activation in antitumorigenic signaling involves the potentiation of the Ca^2+^ emission channel mucolipin-1 (MCOLN1/TRPML1) through palmitoylation of cysteine residues Cys565, Cys566, and Cys567 within the L564CCC motif on its C-terminal tail.^279^ However, it remains unclear whether all three cysteine residues undergo palmitoylation or if modifications to Cys565 and Cys567 are sufficient to optimize Cys566 palmitoylation (Table 1, Fig. 8a).

### EGFR pathway

ROS promote ligand-independent initiation of EGFR in several ways, involving EGFR phosphorylation at the Tyr845 residue,^280^ and/or inhibition of EGFR inhibitory PTPs (at Cys124 and Cys71) and/or Cys797 oxidation of EGFR.^281^ Interestingly, EGFR^T790M^ activation was observed to promote NOX2-regulated ROS formation, which in turn promoted the oxidation of the Cys797 and Met790 residues of EGFR.^282,283^ While Met790 oxidation is reversible, Cys797 oxidation on EGFR^T790M^ further upregulates EGFR-NOX2, which increases ROS production, resulting in aberrant EGFR expression. This excessive phosphorylation of EGFR with a disrupted dimer structure leads to the development of TKI resistance in ROS-exposed EGFR-sensitive NSCLC cell lines.^282,283^ On the other hand, high ROS levels have been reported to induce apoptosis and surmount TKI resistance, as when exposed to excessive ROS, ASK1 potentiation results in the initiation of JNK and p38 MAPK and the induction of apoptosis.^284^ Leung et al. suggested that Sanguinarine aggregated excessive ROS by promoting the activation of NOX3 and consequential inactivation of MsrA and Cys peroxidation of EGFR^T790M^ in EGFR-modulated TKI-resistant cells, which ultimately led to EGFR degradation and cell death^284^ (Table 1, Fig. 8b).

### Ras/MAPK (ERK) pathway

The most prominent mechanism of ROS-mediated ERK activation starts with the potentiation of EGFR. Once activated, EGFR interacts with SHC, leading to Tyr317 phosphorylation, which facilitates Grb2 binding and the formation of the SHC-Grb2-SOS complex, which is modulated by hydrogen peroxide.^281^ After relocating to the plasma membrane, SOS activates Ras by promoting GTP binding.^281^ Activated Ras initiates Raf, which phosphorylates MEK1 (Ser218, Ser222) or MEK2 (Ser222, Ser226), and MEK1/2 then activates ERK by phosphorylating Tyr185 and Thr183.^285^ In addition, SRC kinases activated by ROS mediate ERK activation via Raf or EGFR signaling.^286^ Furthermore, ROS can mediate the Ras-dependent activation of ERK by oxidizing the Cys118 residue of Ras. In addition, commensal bacterium-induced ROS oxidize DUSP3 Cys124, relieving ERK inhibition and activating ERK^287^ (Table 1, Fig. 8b).

### JNK (MAPK) pathway

ROS-induced JNK pathway activation involves MAPKKK (MEKK1/2/3/4, MLK, and ASK1)-induced phosphorylation of a MAPKK (MEK3/4/6/7) at Ser or Thr residues, which then phosphorylates a highly critical Thr-Pro-Tyr (TPY) motif of JNK, leading to the induction and nuclear translocation of JNK.^288^ ROS reversibly oxidize TRX at the Cys32 and Cys35 residues, dissociating it from ASK1 (activation), which, in turn, activates JNK.^289^ Additionally, ROS dimerize GSTP, disrupting its ability to suppress JNK, thereby enhancing JNK activity, with potential dimerization sites at Arg70, Arg74, Asp90, Asp94, and Thr67.^290^ Besides, ROS directly activate ASK1 through homo-oligomerization and autophosphorylation at Thr838 or hetero-oligomerization with ASK2, where ASK2 phosphorylates Thr838 of ASK1 after its own autophosphorylation at Thr806^291^ (Table 1, Fig. 8b).

### p38 (MAPK) pathway

ROS-induced p38 activation mechanism involves several initial proteins involved in the JNK pathway, such as ASK1. ROS directly or indirectly affect ASK1, MEKK1/2/3/4, and MLK3, which subsequently activate MEK3/6 (highly conserved in the p38 pathway).^288^ MEK activation further induces double phosphorylation of a critical TPY motif of p38 on tyrosine and threonine residues.^288^ Besides, ROS have been found to mediate heterodimerization between Cys104 of MKK3 and Cys119 and Cys162, which forms a disulfide bond between p38α and MKK3, leading to p38α overexpression^292^ (Table 1, Fig. 8b).

### PARP1 pathway

Four PARP-1 domains—F1, F3, WGR, and CAT (catalytic)—are crucial for DNA damage-dependent activity, whereas the CAT domain, comprising the Helical Domain (HD) and ADP-ribosyltransferase (ART), is vital for activation.^293^

Under oxidative stress, ROS-induced DNA damage triggers HD unfolding via a “leucine switch” (Leu698 and Leu701 repositioning), relieving autoinhibition and enabling PARP1 DNA binding and autoPARylation.^293^ Regulatory factors like histone PARylation factor 1 (HPF1) modulate PARP1 activity during stress-induced DNA damage. HPF1 interacts with several critical residues (Trp1014, Ser1012, Leu985, His826, and Leu1013) in the catalytic domain of PARP1 through its Lys307, Asp283, Cys285, Phe268, and Phe280 residues in the C-terminal domain.^294–296^ This binding induces ADP-ribosylation of serine residues in the HD domain of PARP1 (Ser499, Ser507, and Ser519).^295,296^ HPF1 also recruits Arg239 to position Glu284 for serine ADP-ribosylation, relieving PARP1 HD autoinhibition. The PARP1-HPF1 complex enhances ADP-ribosylation on Asp/Glu/Ser residues in histones, PARP1, and chromatin factors^294,296^ (Table 1, Fig. 8b).

### ATM pathway

At moderate ROS levels, a few enzymes like PRDX2 and TRX1, can induce chemical alteration of the Cys2991 residue on ATM to generate a reversible disulfide bond.^297^ Upon dimerization, ATM undergoes autophosphorylation at the Ser1981 residue, leading to the initiation of ATM.^298^ Under excessive ROS levels, ATM triggers downstream sites such as p53 and checkpoint kinase 2 (CHK2) for activation even when the DNA damage response (DDR) is absent, indicating that the activation pathway has no impact on the role of ATM at a certain level, whereas ATM has many other functions independent of the DDR^297^ (Table 1, Fig. 8b).

### p53 pathway

Upon oxidative DNA damage, ROS-activated ATM autophosphorylates and activates p53 (via Ser15 phosphorylation) and CHK2, which further phosphorylates p53 at Ser20.^299^ Stress-activated JNK and p38MAPK have also been shown to affect p53 activation upon exposure to H_2_O_2_.^299^ p38 MAPK has been found to induce the phosphorylation of p53 at Ser15, 33, 37, and 46,^299^ whereas JNK has been implicated in the phosphorylation of p53 at the Thr81 and Ser20 residues.^300^ As mentioned earlier, H_2_O_2_ expression results in dimerization and subsequent initiation of ASK1, which initiates p38 MAPK and JNK, stabilizing p53.^288^ Notably, DNA damage-induced p53 activation is typically modulated by ATM, whereas redox signaling-dependent p53 activation is regulated by p38 MAPK signaling.^299^ Although diamide-induced ROS initiate both JNK and p38 MAPK, p53 activation by diamide is regulated entirely by p38 MAPK.^299^ Interestingly, while H_2_O_2_ activates all the ATM, JNK, and p38 MAPK signaling, H_2_O_2_-induced p53 activation requires only ATM and JNK^299^ (Table 1, Fig. 8b).

### AMPK pathway

H_2_O_2_ activates AMPK in HEK-293 cells via an AMP/ADP-dependent pathway involving mitochondrial respiratory chain inhibition.^301^ Another mechanism by which hypoxia-induced ROS activate AMPK is via an AMP-independent pathway involving Ca^2+^ release from the ER. Stromal interaction molecule 1 (STIM1) detects ER calcium depletion, translocates to ER-plasma membrane junctions, and activates Ca^2+^ release-activated channels (CRAC) by tethering Orai proteins, triggering CaMKKβ activation, which phosphorylates AMPK at Thr172.^302^ In addition, H_2_O_2_ directly activates AMPK in HEK-293 cells by oxidatively modifying Cys299 and Cys304 of the AMPKα subunit^303^ (Table 1, Fig. 8b).

However, Hinchy et al. reported decreased AMPK activity after 30 min of H_2_O_2_ addition, as ATP/ADP ratios were restored and PRDX-SO_2/3_ formation was increased.^304^ The underlying mechanism might be that oxidative stress oligomerizes PRDXs, which may compete with other cellular proteins for thioredoxin-catalyzed cysteine thiol reduction.

## Exploring ROS and ROS-driven mechanisms for therapeutic targeting

In pancreatic cancer, oxidative stress initially activates TIGAR and Nrf2 for survival, but prolonged stress reduces TIGAR, increases ROS, and promotes metastasis.^305^ Blocking antioxidants delays tumor initiation but accelerates metastasis, whereas antioxidant intervention (e.g., N-acetyl-L-cysteine) suppresses metastasis in TIGAR-deficient models. In melanoma and lung cancer, antioxidants have been shown to promote metastasis, underscoring the potential efficacy of a pro-oxidant approach in this context.^306,307^

Additionally, the dual roles of ROS enable the development of context-specific therapeutic strategies, ranging from ROS induction to scavenging, for combination cancer therapies. Cancer cells exhibit exceptional mitochondrial plasticity and metabolic reprogramming, allowing them to adapt to the nutrient-deficient tumor microenvironment and evade single-agent therapies by activating alternative survival pathways.^308^ Although excessive ROS can effectively induce cancer cell death and serve as a promising anticancer strategy, they may also cause immunosuppression. Thus, combining ROS-elevating therapies with immunotherapy could optimize outcomes by counteracting immune suppression while targeting cancer cell resilience.

Targeting downstream molecules critical for ROS-mediated pathways, such as the NRX (Wnt/β-catenin), IKK (NF-κB), and ASK1 (MAPK) pathways, might be a potential antitumor therapeutic strategy. By targeting these signaling nodes, this strategy precisely modulates ROS-driven pathways, counteracting ROS-induced signaling without necessarily altering ROS levels. Finally, the heterogeneity of redox responses across cancer types, patients, and stages emphasizes the need for personalized approaches. A comprehensive set of parameters—including redox status, antioxidant enzyme expression, cell signaling profiles, and cancer-specific signaling—constituting a “redox signaling signature” for individual patients, is currently awaiting development.^309^ Notably, regardless of the therapeutic strategy, ROS-targeted therapies fall into two main categories: modulating redox adaptation mechanisms (antioxidant defenses) and targeting ROS-generating systems (e.g., NOX, iNOS, and mitochondria).

### Therapeutic leverage of ROS depletion in chemoprevention and cancer treatment

Antioxidants are commonly used as over-the-counter supplements to prevent cancer or in combination with chemo-/radiotherapy to reduce side effects and improve patients’ quality of life during treatment. This section and Table 2 summarize antioxidant agents with antineoplastic potential that modulate ROS in cancer and their ROS-mediated mechanisms of action.

**Table 2:** Antioxidant agents with antineoplastic potential that modulate ROS in cancer and their ROS-mediated mechanisms of action

Antioxidants	Current status in Cancer therapy			Indications	Drug Target	Key findings/ROS suppressive anticancer mechanism	Refs.
	Phase	NCT/NDA ID	Status				
N-Acetyl-L-cysteine (NAC)	II	NCT04481035	Active, not recruiting	Neurofibromatosis type 1	Glutathione synthetase	NAC → ↓ROS → ↓p-AKT → ↓Telomerase activity → ↓Cell viability.	^310^
	PCS: SMMC-7721 HCC cell lines			Hepatocellular carcinoma			
Diphenylene iodonium	PCS: Colon cancer cell lines			Colitis-associated Colon cancer	NOX	DPI→ ↓ROS → ↓NF-κB/STAT3/ERK → ↓M1 macrophages → ↓Inflammation → ↓Tumor number/size/load, ↓ High-grade neoplasia.	^312^
GKT137831	PCS: NSCLC cells			NSCLC	NOX1/4	GKT137831 → ↓ NOX4 → ↓ROS → ↓YY1 → ↓IL-8 → ↓PD-L1 → ↓Angiogenesis/Tumor growth → ↑TKIs sensitivity.	^313^
Setanaxib (Optimized Successor of GKT137831)	PCS: CAF-rich murine tumor models			Lung, colon, Breast cancer	NOX4	Setanaxib → ↓NOX4 in CAFs → ↓ROS → CAF “Normalization” →↓Immune suppression → ↑CD8, T-cell infiltration → Overcoming αPD-1/PD-L1 resistance.	^314^
Cyclodiaryl-iodonium (CDAI)	PCS: PDX model			Pancreatic cancer	NOX	CDAI → ↓NOX → ↓Non-mtROS, ↑mtROS (via ΔΨm depolarization) → ↓Respiration/↓Glycolysis →↓p53/NF-κB/GnRH → Selective tumor cell death.	^315^
Fangchinoline (Fan)	PCs: NSCLC cells			NSCLC	NOX4	Fan → ↓NOX4 → ↓ROS → ↓AKT/mTOR signaling → EMT reversal → ↓Invasion & Migration → ↓Metastasis.	^316^
Vitamin E	I	NCT00985777	Completed	Pancreatic Exocrine Neoplasia	Peroxyl radicals	Vitamin E → ↑Caspase-3 activation → Selective apoptosis in neoplastic cells → Tumor suppression.	^317^
Lycopene	PCS: PANC-1 cell lines			Pancreatic cancer	Singlet oxygen (^1^O_2_)	Lycopene → ↓ROS/OCR → ↓MMP → ↓NF-κB activation → ↓cIAP1/cIAP2/survivin → ↑BAX/BCL-2 ratio → ↑Caspase-3 → Apoptosis.	^321^
Dimethyl fumarate	II	NCT02546440	Completed	Cutaneous T-cell lymphoma	Keap1	↓mSWAT score, ↓NF-κB, ↓Pruritus	^322^
Sulforaphane	II	NCT00843167	Completed	Breast cancer		↓Ki-67 in DCIS tissue	^324^
	NA	NCT01265953	Completed	Prostate cancer		↓AMACR, ↓ARLNC1 (Genes linked to prostate cancer pathogenesis)	^325^
Resveratrol	I	NCT00433576	Withdrawn	Colorectal cancer		39%↑Caspase-3	^326^
	I	NCT00920803	Completed	Colorectal cancer		5%↓Ki-67	^327^
Bardoxolone-methyl (CDDO-Me, RTA402)	I	NCT00508807	Completed	Advanced solid tumors		↑Nrf2, ↑NQO1/γ-GCS, ↓NF-κB/STAT3, ↓Cyclin D1/COX-2/iNOS, ↑TUNEL, 10×↑Macrophages.	^331^
	I	NCT00529438	Completed	Advanced malignancies		↑NQO1, ↓NF-κB, ↓Cyclin D1	^332^
L-NMMA	I/II	NCT02834403	Completed	TNBC	iNOS	45.8% OR, 27% CR, ↓M2 markers, ↓IL-6/IL-10, ↑CD15+ Neutrophils, ↓Tumor arginase.	^340^

*ROS* Reactive Oxygen Species, *↓* Inhibition/Depletion, *↑* Activation/Release, *HCC* Hepatocellular Carcinoma, *NOX* NADPH Oxidase, *DPI* Diphenyleneiodonium, *NF-κB* Nuclear Factor kappa-light-chain-enhancer of activated B cells, *STAT3* Signal Transducer and Activator of Transcription 3, *ERK* Extracellular Signal-Regulated Kinase, *AML* Acute Myeloid Leukemia, *MRC* Mitochondrial Respiratory Chain, *ΔΨm* Mitochondrial Membrane Potential, *OXPHOS* Oxidative Phosphorylation, *PDX* Patient-Derived Xenograft, *mtROS* Mitochondrial ROS, *GnRH* Gonadotropin-Releasing Hormone, *GSH* Glutathione, *SOD* Superoxide Dismutase, *CAT* Catalase, *MDA* Malondialdehyde, *AOPP* Advanced Oxidation Protein Products, *BCL-2* B-cell Lymphoma 2, *BAX* BCL-2-associated X protein, *HLECs* Human Lymphatic Endothelial Cells, *CCL21* Chemokine (C-C motif) Ligand 21, *NSCLC* Non-Small Cell Lung Cancer, *YY1* Yin Yang 1, *IL-8* Interleukin-8, *PD-L1* Programmed Death-Ligand 1, *TKIs* Tyrosine Kinase Inhibitors, *CAFs* Cancer-Associated Fibroblasts, *αPD-1/PD-L1* Anti-PD-1/PD-L1, *TNBC* Triple-Negative Breast Cancer, *pSTAT3* Phosphorylated STAT3, *OCR* Oxygen Consumption Rate, *MMP* Matrix Metalloproteinase, *cIAP1/2* Cellular Inhibitor of Apoptosis Protein 1/2, *mSWAT* Modified Severity-Weighted Assessment Tool, *AMACR* Alpha-Methylacyl-CoA Racemase, *ARLNC1* Androgen Receptor-regulated Long Non-Coding RNA 1, *DCIS* Ductal Carcinoma In Situ, *NQO1* NAD(P)H Quinone Dehydrogenase 1, *γ-GCS* Gamma-Glutamylcysteine Synthetase, *COX-2* Cyclooxygenase-2, *iNOS* Inducible Nitric Oxide Synthase, *TUNEL* Terminal deoxynucleotidyl transferase dUTP Nick End Labeling, *OR* Objective Response, *CR* Complete Response

#### Modulation of GSH metabolism

N-acetyl-L-cysteine (NAC) supports GSH metabolism by providing cysteine for GSH replenishment. A preclinical study suggested its selective anticancer potential by depleting ROS below the levels required for telomerase activation, disrupting redox homeostasis, and suppressing cancer cell proliferation.^310^ Interestingly, NAC paradoxically lowers the GSH/GSSG ratio, as cancer cells require some ROS to maintain antioxidant capacity. Besides, reduced glutathione and NOV-002 are being clinically investigated for their potential to improve the quality of life of patients receiving chemo-/radiotherapy.

#### Inhibition of NOX-mediated ROS generation

Agents capable of targeting NOXs to scavenge ROS could offer considerable potential for cancer therapy. Among several small-molecule NOX inhibitors, Ebselen is under clinical study for mitigating chemotherapy-induced toxicity.^311^ Diphenylene iodonium (DPI) suppresses early tumorigenesis in colitis-associated cancer by inhibiting ROS-driven NF-κB, STAT3, and ERK pathways, reducing inflammation.^312^ GKT137831 disrupts the NOX4-ROS-YY1-IL-8-PD-L1 axis, lowering IL-8 and PD-L1 levels to impair angiogenesis, immune evasion, and Gefitinib resistance.^313^ The optimized successor of GKT137831, Setanaxib, overcomes αPD-1/PD-L1 resistance by targeting NOX4 in cancer-associated fibroblasts, reversing their immunosuppressive phenotype, and enhancing CD8^+^ T-cell infiltration.^314^ Cyclodiaryl-iodonium (CDAI) kills pancreatic cancer cells by blocking NOX, reducing nonmitochondrial ROS while inducing lethal mitochondrial ROS, disrupting energy metabolism and triggering p53/NF-κB/GnRH pathway chaos with minimal toxicity.^315^ Fangchinoline inhibits NSCLC metastasis by reversing EMT and suppressing the NOX4-ROS-AKT-mTOR pathway.^316^

#### Direct ROS scavenging

ROS scavengers directly neutralize ROS by donating electrons or hydrogen atoms. ROS scavengers, such as Vitamin E, Vitamin C, Vitamin D, Melatonin, Carotenoids, and Lycopene, interact with ROS by donating electrons or hydrogen atoms to free radicals. To date, numerous studies have explored the antineoplastic potential of ROS scavengers. Among them, a phase I clinical trial (NCT00985777) provided evidence that Tocotrienol (Vitamin E) may induce apoptosis in PDAC cells.^317^ Regarding anti-inflammatory activity, Vitamin D3 has been reported to decrease TNF-α-induced inflammation in lung epithelial cells through a reduction in mitochondrial fission and mitophagy.^318^ A study reported that Melatonin inhibits angiogenesis by downregulating the HIF1α/ROS/VEGF pathway in HUVECs,^319^ while its pro-oxidant activity may also contribute to anticancer effects, highlighting a context-dependent mechanism that warrants further investigation.^320^ A phase II trial (NCT00450749) suggested that Lycopene may slow prostate cancer growth, although the results were inconclusive. In PANC-1 cells, lycopene inhibits ROS-mediated NF-κB signaling and induces apoptosis.^321^

#### Keap1 inhibition

Keap1 inhibition activates Nrf2 to upregulate antioxidant enzymes that neutralize ROS. For example, in cutaneous T-cell lymphoma (CTCL), DMF therapy has shown clinical efficacy by lowering mSWAT scores, relieving pruritus, and inhibiting NF-κB, as demonstrated in the NCT02546440 trial.^322^ Sulforaphane targets Cys151 to inhibit the Keap1–Nrf2 interaction, boosting NAD(P)H:NQO1 expression and reducing lung inflammation in vivo.^323^ A clinical trial (NCT00843167) revealed that Sulforaphane reduces the expression of Ki-67 and other proliferation markers in DCIS and high-risk lung cancer patients.^324^ Another trial (NCT01265953) revealed that Sulforaphane downregulates the prostate cancer-associated genes AMACR and ARLNC1.^325^ Besides DMF and Sulforaphane, other Keap1 inhibitors include Resveratrol, Quercetin, RTA-402, and RTA-408. Resveratrol induces caspase-3-mediated apoptosis (39% increase; NCT00920803) and reduces Ki-67-linked proliferation (5% decrease; NCT00433576) in hepatic metastases and colorectal cancer.^326,327^ Quercetin activates Nrf2/Keap1 to reduce ROS,^328^ while inducing ROS-dependent cell death in malignant cells,^329,330^ underscoring its potential as both a chemopreventive and chemotherapeutic agent. Bardoxolone methyl (RTA 402) upregulates Nrf2 targets, suppresses NF-κB and cyclin D1, and induces tumor regression in advanced cancers. In trials (NCT00529438, NCT00508807), it achieved responses in several hematologic and solid tumors, with stabilization lasting 6–12 months.^331,332^ Omaveloxolone (RTA 408) has also been reported to induce time- and dose-dependent activation of Nrf2 antioxidant genes (NCT02029729). In addition, Genistein and Procyanidin B2 reduce carcinogen-induced ROS and DNA damage via Nrf2/ARE signaling in bronchial epithelial cells.^333^

#### Utilization of SOD mimetics

Although SOD mimics have a lower rate constant than natural SOD enzymes, they effectively function in extracellular fluids lacking antioxidant enzymes. SOD mimics include Metalloporphyrins, Mn (II) polyamines, Mn(III) salens, Mn(III) corroles, and Mn(IV) biliverdins.^334^ The Mn(II)-based compound GC4419 showed safety but no remission in mCRPC (NCT01080352). Additionally, certain SOD mimics like Motexafin gadolinium, act as both prooxidative and antioxidative agents.

#### Xanthine oxidoreductase (XOR) inhibition

Inhibiting XOR (Xanthine oxidase and Xanthine dehydrogenase) offers a promising cancer therapeutic approach for reducing ROS generation during purine catabolism, thereby mitigating DNA damage, inflammation, and tumor progression.^335^ Preclinical studies have shown that XOR inhibition enhances the effectiveness of the TKIs currently used in clinics against BCR-ABL in CML.^336^ *Hericium erinaceus,* an edible mushroom, may treat breast cancer by inhibiting Xanthine oxidase, scavenging ROS, and suppressing cancer cell growth with minimal impact on normal cells.^337^

#### iNOS inhibition

iNOS (inducible NOS) is highly overexpressed in many cancers and is correlated with poor prognosis in TNBC, making it a promising biomarker and therapeutic target.^338^ Therefore, inhibiting iNOS can reduce ROS production, suppress tumor growth, and modulate immune responses. Andrographolide was shown to exert antitumor activity across multiple cancers by inhibiting iNOS.^339^ In the NCT02834403 trial, combining L-NMMA with Taxane showed efficacy in treating chemorefractory breast cancer, particularly in advanced cases, with manageable toxicity.^340^

### Therapeutic leverage of ROS elevation in cancer treatment

Since the 1950s, various strategies/drug research have been implemented based on this concept, such as administering ROS or ROS-generating enzymes to tumor cell lines or murine models with tumors.^335^ As documented in this section and Table 3, antineoplastic agents/methods with ROS-inducing potential and their ROS-mediated antitumor mechanisms—compiled from sources including the FDA database (www.fda.gov), ClinicalTrials.gov, and DrugBank (http://go.drugbank.com)—demonstrate diverse mechanisms of action ranging from metabolic interference to direct oxidative damage.

**Table 3:** Antineoplastic agents/methods with ROS-inducing potential and their ROS-mediated antitumor mechanisms

Name	Clinal trial (CT)/FDA approval/PCS					Indication	Target	Key findings/ROS-mediated anticancer mechanism	Refs.
	Phase	NDA/NCT ID			Status				
Buthionine sulfoximine (BSO)	I	NCT00002730			Completed	Neuroblastoma	GSH	BSO → ↓GSH→ Disrupted ETC →↓ΔΨm →↑ROS→Cell death.	^342^
Imexon	1	NCT00327288			Completed	NSCLC, breast, prostate cancer		Imexon → Binds thiols (↓GSH) →↑ROS → ↓Δψm →Apoptosis.	^343^
OSamp	PCS: SW620 Colon cancer xenograft mouse model					Colon cancer		OSamp → Esterase/acid hydrolysis → ↓GSH + ↑ROS → ↓ΔΨm → Cyt c release → ↑Caspase-3, ↓STAT3 → Apoptosis.	^344^
LBL21	PCS: multiple cancer models					NSCLC		LBL21 → ↓ GSH → ↑ ROS →↓ΔΨm →↓CSC-SP cells →↓Tumor growth.	^346^
PEITC	NA	NCT03034603			Completed	Head and neck cancer	GSH, ETC-III, UPR	↓GSH, reactivation of p53, ↑SD response, ↑PFS.	^347^
Benzoyloxy dibenzyl carbonate (B2C)	PCS: Multiple cancer xenograft mouse models					Various cancers	GSH	B2C→ ↓GSH → ↑ROS → ↓ΔΨm → Caspase-3/PARP1 activation, BCL-2 cleavage → Apoptosis.	^348^
ACZ2	PCS: GC Xenograft mouse model					Gastric cancer	GSH, TRXR1	ACZ2 → ↓GSH/TRXR1 → ↑ROS → ↑PERK/EIF2α/ATF4/CHOP → ER stress → Apoptosis, Autophagy.	^349^
PX-12	II	NCT00417287			Terminated	Advanced pancreatic cancer	TRX1	PX-12 → ↓TRX1 → ↑ROS → ↑Lipid peroxidation→ Ferroptosis.	^353^
	I	NCT00736372			Completed	Metastatic cancer			
Arsenic trioxide	NA	NDA: 021248 (For RA)			NA	Leukemia	TRXR1	ATO →↓ TRX1 → ASK1 activation → ↑SEK1 & JNK → ↑Apoptosis signaling.	^355^
Auranofin	II	NCT01419691			Completed	Lymphocytic leukemia Ovarian cancer	TRXR	Auranofin → ↓TRXR → ↑ROS →↓ΔΨm → Caspase-3/7 activation, DNA damage → Apoptosis.	^356^
	I	NCT01747798			Completed				
Motexafin gadolinium (MGD)	II	NCT00134186			Completed	Kidney neoplasms	TRX	MGD → ↓ TRX → ↑ ROS/ Oxidative stress →↓DNA repair → Apoptosis.	^357^
	II	NCT00129844			Completed	NSCLC			
WZ35 (curcumin-derived analogs)	PCS: Human gastric cancer cell lines					Gastric cancer	TRXR	WZ35 → binds TRXR (Sec-498) → ↓ TRXR → ↑ROS→ Apoptosis → Cell death.	^358^
Piperlongumine (PL)	PCS: Colorectal cancer cells					Colorectal cancer	TRXR, GSH	PL →↓TRXR/GSH →↑mtROS → DNA damage, G2/M cell cycle arrest → ↑RT efficacy.	^359^
Shikonin	PCS: NSCLC cells					NSCLC	TRXR1	Shikonin → Binds TRXR1 (Sec498) → ↓TRXR → Retains NOX activity → ↑ O_2_•^−^ → ↑ ROS → Necroptosis.	^360^
LW-216	PCS: NCI-H460 Lung cancer xenograft mouse model					NSCLC	TRXR1	LW-216 → Binds TRXR1 (Arg371/Gly442) → ↓TRXR1 → ↑ROS → DNA damage → Apoptosis.	^361^
6-Shogaol	PCS: HeLa cells					Cervical cancer	TRXR	6-Shogaol → ↓TRXR1 → ↑ROS → Apoptosis → Selective cytotoxicity (HeLa > Normal cells).	^362^
Nitrovin	PCS: Zebrafish xenograft model					Glioblastoma	TRXR	Nitrovin → Cytoplasmic vacuolation, ↑ROS → ↑MAPK, ↓Alix → Paraptosis (Caspase-3 independent) → Cell death.	^363^
ATN-224	II	NCT00405574			Unknown	Prostate cancer	Superoxide Dismutase1 (SOD1)	ATN-224 → ↓SOD1 → ↑ROS → ↓ΔΨm → Cell death.	^364,365^
TRIM22	PCS: Breast cancer cell lines					Breast cancer		TRIM22 → ↓CCS → ↓SOD1 → ↑ ROS → ↓STAT3→ ↓Tumor growth.	^366^
ARQ761	I	NCT01502800			Completed	Advanced solid tumors	NQO1	ARQ761 →Futile redox cycling →↑ O_2_•^−^/H_2_O_2_ → DSB → PARP1 hyperactivation → ↓NAD^+^/ATP → Apoptosis.	^367^
IP-DNQ	PCS: NQO1^+^ pancreatic cancer cells					Pancreatic cancer		IP-DNQ → ↑ROS → DSB → PARP1 hyperactivation → Mitochondrial catastrophe, G2/M arrest → Apoptosis/Necrosis.	^370^
Cryptotanshinone (CTS)	PCS: NQO1 high-expressing H460 lung cancer xenograft mouse model					Lung cancer		CTS → Binds NQO1 → ↑ROS → ↑JNK1/2 + PARP → ↑Iron/Ca^2+^ → ↑ATP/NAD^+^ → Necrosis.	^371^
Daphnetin (Daph)	PCS: Ovarian cancer mouse xenograft model					Ovarian cancer		Daph → ↓NQO1 → ↑ROS → ↓TFR/ ↑ SLC40A1/ ↓ SLC7A11/ ↓ GPX4 → Fe^2+^ accumulation, Lipid peroxidation → Ferroptosis.	^372^
CB-839	CT: Multiple cancers (Combination Therapy)					Different cancers	Glutaminase	CB-839 → ↓Glutamate → ↓GSH synthesis → ↑ROS → Oxidative stress → Cancer cell death.	^129^
BIX01294	PCS: PDAC cells					PDAC		BIX01294 → ↓KDM6B → ↑Repressive histone marks (H3K27me3) at GLS promoter → ↓GLS → ↓Glutamine metabolism → ↑NADP ^+^ /NADPH ratio + ↓GSH → ↑ROS → ↑Caspase → Apoptosis.	^374^
2-DG	I	NCT00247403			Withdrawn	Prostate cancer, solid tumors, intracranial neoplasms	Hexokinase (Glycolysis)	2-DG → ↓Hexokinase→ ↓Glycolysis→ ↑ROS → ↑Caspase → Apoptosis.	^375^
Chloroquine (CQ)	PCS: colorectal cancer HCT-116 cells					Colorectal cancer		CQ → ↓PDK1 → ↓Glycolysis →↓Δψm → ↑ROS → Apoptosis.	^376^
Lenvatinib	PCS: HCC xenograft mouse models					HCC	GPX2	Lenvatinib → ↓β-catenin nuclear translocation → ↓GPX2 → ↑ROS→ apoptosis → HCC cell death.	^377^
FIN56	PCS: Bladder cancer cells					Bladder cancer	GPX4 protein	FIN56 → ↓ GPX4 protein → ↑ROS→Autophagy→Ferroptosis.	^378^
Sulfasalazine	PCS: Several cancer types					Multiple cancers	xCT/GPX4	SAS →↓xCT/GPX4 → ↑ROS → Ferroptosis.	^379^
Erastin	PCS: Several cancer types					Multiple cancers	xCT/GSH/GPX4	Erastin →↓xCT/GPX4 → ↓ GSH → ↑ROS/MDA → Ferroptosis.	^381^
1,2-Dioxolane (FINO2)	PCS: HT-1080					Fibrosarcoma	GPX4	FINO2 → ↓ GPX4 (indirectly), ↓Co-Q10, ↑Iron oxidation→ ↑ROS → ↑ LPO→ Ferroptosis.	^382^
Bortezomib	FDA Approved, NDA ID: 215441					Multiple myeloma	Proteosome	Bortezomib → ↓26S proteasome → ↑UPA → ↑ROS → NF-κB modulation → ↑NOXA → BCL-2 phosphorylation → p53 stabilization → ↑Caspase → Apoptosis.	^384^
CEP-18770	PCS: DAOY and UW228-2 cell lines					Medulloblastoma		CEP-18770 →↓Proteasome → ↑UPA → ↑ ER stress/ROS → p73 stabilization → DNA damage (γH2AX) →↑Caspase → Apoptosis → MB cell death.	^386^
Tryptanthrin (TRYP)	PCS: Huh7 Liver cancer xenograft mouse model					Liver cancer	GSTP1	TRYP → ↓GSTP1 → ↑ROS → DNA damage → ↑NF-κB → SASP → Senescence→ ↑Susceptibility to apoptosis by ABT263 (BCL-2 inhibitor).	^388^
β-Sitosterol	PCS: OC Xenograft mouse model					Ovarian cancer	ASS1/Nrf2	β-Sitosterol → Targets ASS1 → ↑Nrf2-Keap1 interaction → ↑Nrf2 ubiquitination/degradation → ↓HO-1/NQO1 → ↑ROS → ↑PTEN → ↓p-AKT → OC cell death.	^390^
ZIO-101	I		NCT00592046	Completed		Hematologic cancer	NOX, mitochondrial disruption	ZIO-101 → ↑ NOX (p67PHOX/ p47PHOX) →↑ROS → ↓ΔΨm→Cell death.	^56,391^
GNF362	PCs: Glioblastoma cell lines					Glioblastoma	ITPKB/NOX	GNF362 → ↓ ITPKB-mediated NOX suppression→ ↑ NOX→ ↑ ROS → Restored TMZ sensitivity → Cancer cell death.	^392^
Elesclomol	I	NCT00827203			Suspended	Solid tumors	mitochondrial metabolism	Elesclomol → Cu (^2+^) shuttling → Mitochondrial Cu (II) accumulation → ↑ROS → FDX1 reduces Cu (^2+^) to Cu (^+^) → Lipoylated DLAT oligomerization → Cuproptosis.	^393^
Fenretinide	NA	NCT02075177			NA	Neuroblastoma	ETC-II	Fenretinide→ ↑mtROS (Complex II-dependent) → Cytoplasmic vacuolization (endosome accumulation) → Dynamin-dependent vesicle formation → Cell death (apoptosis/vacuole-associated).	^394^
Atovaquone	PCS: Fhit-deficient lung cancer cell line					Lung cancer	ETC-III	Atovaquone → ↓ETC -III → ↑mtROS → DNA damage → Apoptosis.	^395^
PDT	III	NCT00472108			Completed	Basal cell carcinoma	¹O₂	m-THPC-PDT or VP-PDT→ Generates ¹O_2_/free radicals → ↑ROS → ↑ JNK →Autophagy/Apoptosis →Cell death.	^401^
	III	NCT00473343			Completed				
	III	NCT00472043			Completed				

*CT* Clinical Trial, *PCS* Preclinical Study, *SAS* Sulfasalazine, *TRXR* Thioredoxin Reductase, *TRX* Thioredoxin, *GSH* Glutathione, *ROS* Reactive Oxygen Species, *↓* Inhibition/Depletion, *↑* Activation/Release, *ΔΨm* Mitochondrial Membrane Potential, *AIF* Apoptosis-Inducing Factor, *CG* Calycosin-7-glucoside, *SOD1* Superoxide Dismutase 1, *CcOX* Cytochrome c Oxidase, *Nrf2* Nuclear Factor Erythroid 2–Related Factor 2, *HO-1* Heme Oxygenase-1, *GCLC* Glutamate-Cysteine Ligase Catalytic Subunit, *CCS* Copper Chaperone of SOD1, *GCLM* Glutamate-Cysteine Ligase Modifier Subunit, *EMT* Epithelial-Mesenchymal Transition, *DSB* Double-Strand Break, *PARP1* Poly(ADP-ribose) Polymerase 1, *NAD+* Nicotinamide Adenine Dinucleotide, *ATP* Adenosine Triphosphate, *IP-DNQ* Isopentyl-Deoxynyboquinone, *CTS* Cryptotanshinone, *NQO1* NAD(P)H Quinone Dehydrogenase 1, *JNK1/2* c-Jun N-terminal Kinase 1/2, *Daph* Daphnetin, *TFR* Transferrin Receptor, *SLC40A1* Ferroportin, *SLC7A11* Solute Carrier Family 7 Member 11 xCT, *GPX4* Glutathione Peroxidase 4, *KDM6B* Lysine Demethylase 6B, *H3K27me3* Histone H3 Lysine 27 Trimethylation, *GLS* Glutaminase, *GLUT1/4* Glucose Transporter 1/4, *HK1/2* Hexokinase 1/2, *PANoptosis* Pyroptosis-Apoptosis-Necroptosis Hybrid Cell Death, *GPX2* Glutathione Peroxidase 2, *Co-Q10* Coenzyme Q10, *LPO* Lipid Peroxidation, *UPA* Ubiquitinated Protein Accumulation, *NF-κB* Nuclear Factor Kappa-Light-Chain-Enhancer of Activated B Cells, *NOXA* Phorbol-12-Myristate-13-Acetate-Induced Protein 1, *p27/p21* Cyclin-Dependent Kinase Inhibitors, *LC3-II* Microtubule-Associated Protein 1A/1B-Light Chain 3-II, *γH2AX* Phosphorylated H2A Histone Family Member X, *p73* Tumor Protein p73, *GSTP1* Glutathione S-Transferase Pi 1, *SASP* Senescence-Associated Secretory Phenotype, *Chac1* Glutathione-Specific Gamma-Glutamylcyclotransferase 1, *MDA* Malondialdehyde, *Keap1* Kelch-like ECH-Associated Protein 1, *HIF1α* Hypoxia-Inducible Factor 1-Alpha, *IRP1* Iron Regulatory Protein 1, *SIT* β-Sitosterol, *ASS1* Argininosuccinate Synthase 1, *OC* Ovarian Cancer, *PL* Piperlongumine, *mtROS* Mitochondrial Reactive Oxygen Species, *FDX1* Ferredoxin 1, *DLAT* Dihydrolipoamide S-Acetyltransferase, *PTX1* Paclitaxel-Resistant Cell Line, *PDT* Photodynamic Therapy, ¹*O*_2_ Singlet Oxygen

#### GSH depletion

GSH inhibition causes severe ROS accumulation and increased oxidative stress, leading to cancer cell death.^341^ BSO, a gamma-glutamylcysteine synthetase inhibitor, disrupts redox balance and sensitizes cancer cells to chemotherapy,^342^ while Imexon generates ROS, causing DNA damage and apoptosis in multiple myeloma.^343^ The hybrid anticancer drug OSamp depletes glutathione and generates ROS, selectively inducing oxidative stress and apoptosis in cancer.^344^ Similarly, PEITC, a cruciferous phytochemical, selectively kills cancer cells by targeting GSH (along with ETC-III and the UPR) to induce ROS-mediated cell death,^345^ with its synthetic analog LBL21 targeting stem-like cancer cells in NSCLC.^346^ A trial revealed that Nutri-PEITC jelly improved PFS and quality of life in advanced oral cancer patients, which was correlated with p53 levels.^347^ Benzoyloxy dibenzyl carbonate (B2C) generates Quinone Methide (QM) intermediates (GSH scavengers) upon hydrolysis, depleting GSH, enhancing ROS accumulation, and showing anticancer therapeutic potential.^348^ Additionally, a recent study identified ACZ2 as a dual GSH/TRXR inhibitor, demonstrating its potential as a ROS-targeted therapeutic against gastric cancer.^349^ Besides, DMF, another KEAP1-inhibiting antioxidant, paradoxically depletes GSH in MS patients, increasing ROS and mitochondrial stress to induce caspase-mediated memory T-cell apoptosis, thereby reducing neuroinflammation (NCT02461069).^350^ Many other studies have also reported ROS inducing potential of DMF,^351,352^ suggesting selective anti-inflammatory mechanism of DMF that warrants further investigation.

#### Thioredoxin inhibition

The dysregulated TRX system often drives tumor progression and therapy resistance, making its targeted inhibition a potent ROS-mediated anticancer strategy. PX-12, a TRX1 inhibitor that binds to Cys73 of TRX, has shown promise across various cancers in preclinical and early clinical trials.^353^ Another TRX1 inhibitor, Diallyl Trisulfide (Allitridin), a garlic-derived compound, inhibits TRX1 via Michael addition (Cys32 and Cys35), radiosensitizing glioblastoma.^354^ Arsenic trioxide (ATO), an FDA-approved APL treatment,^355^ and the repurposed antirheumatic drugs, Auranofin (AF) and Motexafin gadolinium (MGd) inhibit TRXR, inducing ROS to exert antitumor activity.^356,357^ Curcumin has demonstrated anticancer activity through TRXR inhibition in preclinical and early clinical studies.^358^ Piperlongumine (a dual TRXR/GSH inhibitor) demonstrates selective cytotoxicity in colon cancer.^359^ Shikonin’s dual inhibition of TRXR and PKM2 induces necroptosis, whereas Nrf2 hyperactivation mediates resistance, which can be reversed through metabolic targeting.^360^ Novel agents such as LW-216 (Sec498-targeted TRXR1 degradation),^361^ 6-shogaol (selenocysteine inhibition),^362^ and Nitrovin induce caspase-3-independent paraptosis via MAPK/Alix modulation.^363^

#### SOD inhibition

Inhibiting SOD, especially SOD1, prevents superoxide conversion, increasing ROS, thereby selectively targeting cancer cells. ATN-224 (Tetrathiomolybdate), a copper-chelating agent, depletes cellular copper, a cofactor essential for SOD1 activity, causing oxidative stress and cancer cell death, with promising preclinical and clinical results.^364,365^ A recent study suggested that the E3 ligase TRIM22 functions as a tumor suppressor in breast cancer by degrading CCS (copper chaperone of SOD1), thereby reducing the copper supply to SOD1, impairing SOD1 activity, increasing ROS levels, and suppressing STAT3-dependent oncogenic pathways.^366^

#### NQO1 inhibition

NQO1, overexpressed in cancers but minimally expressed in normal tissues, offers a tumor-specific therapeutic target through redox cycling, generating cytotoxic ROS. β- Lapachone (ARQ761) induces DNA damage, PARP1 hyperactivation, and apoptosis in an NQO1-dependent manner (NCT02514031).^367^ Another NQO1 substrate, ARQ501, was shown to cause stable disease in a phase 1 trial,^368^ and DNQ is 10-fold more potent than β-lapachone.^369^ However, these agents cause methemoglobinemia, a limitation overcome by DNQ derivative Isopentyl-DNQ (IP-DNQ) in pancreatic cancer.^370^ Cryptotanshinone (CTS) binds NQO1 noncatalytically, triggering ROS/JNK1/iron/PARP/calcium-mediated necrosis,^371^ while Daphnetin inhibits NQO1 to induce ferroptosis in ovarian cancer.^372^

#### Inhibition of glutamine (Gln) metabolism

Inhibiting glutamine metabolism disrupts GSH and NADPH synthesis, elevating ROS, which impairs tumor growth and induces cancer cell death.^129^ Glutaminase inhibitors, such as CB-839 (Telaglenastat), BPTES, and Compound 968, disrupt Gln metabolism, depleting GSH and increasing ROS to induce cancer cell death.^129^ Additionally, the ASCT2 inhibitor, V-9302 blocks Gln uptake, impairing tumor growth and enhancing immunity via ROS-mediated B7H3 degradation.^373^ A novel glutaminase inhibitor, BIX01294, suppresses pancreatic cancer by inhibiting KDM6B-mediated glutaminase expression, thereby disrupting redox balance.^374^

#### Glycolysis inhibition

Glycolysis inhibitors (2-DG, Metformin, Lonidamine, and 3-BP) target hexokinase to disrupt the Warburg effect in cancer cells, impairing energy production and inducing ROS-mediated selective cell death.^375^ In a recent study, Chloroquine (CQ) was found to inhibit hypoxia-induced growth of colorectal cancer cells by suppressing glycolysis and NAD⁺ production through PDK1 inhibition.^376^ This leads to mitochondrial dysfunction, excessive ROS generation, reduced membrane potential, and apoptosis via PARP cleavage and caspase activation. Despite their antitumor efficacy, glycolysis inhibitors lack selectivity because of the role of glycolysis in normal cells; targeting cancer-specific isoforms (e.g., PKM2) or using nanocarriers could enhance specificity and reduce toxicity.

#### Inhibition of GPXs

GPX inhibition disrupts redox balance, inducing oxidative stress and offering a ROS-targeted cancer therapy approach.^14^ For instance, Lenvatinib blocks GPX2 in liver cancer, causing ROS buildup and apoptosis.^377^ Similarly, targeting xCT/GPX4, a critical regulator of lipid peroxidation, with compounds such as FIN56, Erastin, Sulfasalazine and RSL3 induces ferroptosis.^378–381^ Interestingly, a recent study suggested that RSL3 may induce cell death primarily through the inhibition of TRXR1 rather than the suppression of GPX4, highlighting the need for further studies to elucidate the precise mechanisms by which RSL3 triggers ferroptosis.^380^ FINO2 indirectly inhibits GPX4 activity (unlike Erastin and RSL3) without depleting GPX4 protein (unlike FIN56), while also oxidizing iron to drive ferroptosis.^382^

#### Inhibition of the ubiquitin–proteasome pathway

Proteasome inhibitors represent a promising anticancer strategy by disrupting protein homeostasis and inducing ROS.^383^ Bortezomib treats multiple myeloma by blocking the 26S proteasome, increasing ROS, and upregulating proapoptotic proteins such as NOXA.^384^ Disulfiram (in combination with copper) has been shown to inhibit both the proteasome and SOD1, amplifying oxidative stress.^222^ Experimental inhibitors such as MG132, Epoxomicin, Marizomib, and Lactacystin also induce ROS-dependent apoptosis across various cancers.^385^ Another novel proteasome inhibitor, CEP-18770, demonstrates superior and more sustained dose-dependent suppression of tumor proteasome activity than Bortezomib in medulloblastoma.^386^

#### GSTP inhibition

Overexpression of GSTP is frequently observed in multiple cancers, positioning it as a compelling therapeutic target for ROS-driven anticancer strategies.^387^ While known inhibitors (e.g., Ethacrynic Acid derivatives, NBDHEX, TLK199, LAS17, and CNBSF) exist, their ROS-linked mechanisms remain incompletely characterized.^387^ A recent study identified GSTP1 as the target of Tryptanthrin (TRYP), which binds and inhibits GSTP1, triggering ROS accumulation, DDR, and senescence initiation.^388^

#### Nrf2 inhibition

Nrf2, frequently overexpressed in therapy-resistant cancers, represents a promising therapeutic target for ROS-mediated anticancer strategies, as demonstrated by Tamoxifen-resistant breast cancer cells that develop irradiation cross-resistance through Nrf2-driven antioxidant upregulation and a glycolytic shift, with Nrf2 inhibition restoring radiosensitivity in both cellular models and patient tumors (NCT00738777).^389^ This approach is further validated by the agent β-Sitosterol, which indirectly modulates Nrf2 through ASS1 and then induces ROS-mediated cell death in ovarian cancer.^390^

#### NOX activation

NOX activation drives ROS generation in cancer therapy, exemplified by ZIO-101 (Darinaparsin), which induces NOX-mediated ROS production and mitochondrial disruption, triggering apoptosis in hematologic and solid tumors.^391^ While its precise mechanism—including potential NOX subunit (e.g., p67phox, p47phox) involvement—requires further elucidation, ZIO-101’s dual action of ROS induction and mitochondrial impairment effectively promotes cancer cell death.^56,391^ A study on recurrent glioblastoma observed that elevated inositol 1,4,5-triphosphate (IP3) kinase B (ITPKB)—due to reduced Trim25-mediated degradation—suppresses NOX enzyme activity, lowering ROS production and enabling resistance to Temozolomide (TMZ). Depleting ITPKB or inhibiting it with GNF362 reactivated NOX, increased ROS levels, and restored TMZ sensitivity in resistant GBM cells.^392^

#### Targeting ETC

Elesclomol and Fenretinide exploit this mechanism: Elesclomol binds to mitochondrial copper, disrupting ETC function and increasing ROS,^393^ whereas Fenretinide interferes with mitochondrial complex II to elevate superoxide.^394^ Loss of Fhit protein is observed in most cancers. Atovaquone, a repurposed antimalarial drug, acts as a selective OXPHOS inhibitor by targeting the CoQ10-dependent mitochondrial complex III, causing electron leakage, ROS production, and cell death, as observed in Fhit-deficient lung cancer cell lines.^395^ Phenformin, a metformin analog, inhibits complex I to generate ROS and shows promise in preclinical and early-phase trials for treating HCC and glioma.^396^

#### Pharmacological ascorbate

Ascorbate (vitamin C) acts as a pro-oxidant under certain conditions, generating ROS in the presence of transition metals like iron.^397^ In a Phase II trial (NCT02420314) for advanced NSCLC, Ascorbate combined with Carboplatin and Paclitaxel inhibited tumor growth through H₂O₂ generation, iron modulation (higher transferrin linked to improved PFS), and immune activation (4.2-fold increase in CD8^+^ T cells), achieving a 34.2% response rate and a 12.8-month median OS.^398^ Another trial (NCT02344355) in glioblastoma patients reported improved survival (19.6 vs. 14.6 months), with the greatest benefit in IDH-mutant tumors (53.1 months) and those with high MRI-detected iron, where vitamin C-induced Fe^3+^ reduction to Fe^2+^ enhanced chemo-radiation sensitivity.^397^

#### Conventional anticancer agents

Chemotherapeutic drugs (e.g., Doxorubicin, Paclitaxel, Cyclophosphamide, and 5-Fluorouracil) often induce ROS via a secondary mechanism (including mitochondrial dysfunction, DNA damage, depletion of NAD^+^ and ATP, and destabilization of microtubules) to enhance their cytotoxic effects.^399^ That their anticancer efficacy can be regulated by exposure to antioxidants suggests that their ROS-inducing capacity can be exploited for developing cancer therapies. Ionizing radiation (IR) produces ROS via water radiolysis, causing oxidative damage.^400^ PDT employs photosensitizers that generate ROS, primarily ¹O_2_, upon light activation, causing oxidative damage and inducing cancer cell death.^401^ PDT has shown efficacy in Phase III trials for basal cell carcinoma, improving tumor reduction and quality of life, but is limited by light penetration depth and photosensitivity. A previous study suggested that MLu (lutetium texaphyrin), an analog of MGd, acts as a photodynamic sensitizer under light irradiation to generate excessive ROS, which activate caspase-3. Caspase-3 then cleaves GSDME, releasing its pore-forming N-terminal fragment, thereby leading to pyroptosis.^402^

In addition to conventional approaches, several strategies targeting metabolic vulnerabilities and stress response pathways have emerged as promising ROS-mediated antitumor therapies. ER stress induction—via agents like Bortezomib or natural compounds such as Triptolide—triggers UPR activation and ROS-mediated cell death in malignancies with a high proteostatic burden.^403^ IDH1/2 inhibition disrupts NADPH homeostasis, weakens redox defenses and induces oxidative damage. Vorasidenib, a brain-penetrant IDH1/2 inhibitor, showed efficacy in a Phase III trial for IDH-mutant glioma (NCT04164901) by reducing GSH, increasing ROS, and enhancing therapeutic effects.^404^ DDR pathway inhibitors (e.g., ATM, CHK1, and PARP inhibitors) exacerbate oxidative stress by impairing repair processes and antioxidant functions.^405^

### ROS-responsive pathway/cellular process-targeted therapy: potential targets

This approach is particularly relevant in diseases like cancer, where ROS dysregulation promotes pathological modulation of cellular processes/pathways. This section and Table 4 include different potential targets specific to different ROS-mediated cellular processes or pathways, as well as potential therapeutic strategies for mitigating cancer.

**Table 4:** Potential targets and therapeutic strategies for different ROS mediated cellular processes and pathways

Pathway/cellular process	Potential target	Potential therapeutic strategy	Refs.
PI3K/AKT, EGFR, JAK/STAT, and ERK1/2	PTPs	PTP activation	^231^
Wnt/β-catenin	NRX	NRX activation	^71^
ERK	MEK (1/2)	MEK (1/2) inhibition	^285^
JNK	ASK1	ASK1 activation	^288^
p38			
JAK/STAT	JAK2	JAK2 inhibition	^77^
Apoptosis	BCL-2 family, caspase family	Cellular context-based modulation	^157,158^
Ferroptosis	xCT, GPX4, FSP1, DHODH, GCH1	Cellular context-based modulation	^188^
Pyroptosis	NLRP3 inflammasome, GSDMD, caspase family, and NF-κB	Cellular context-based modulation	^202,203^
Necroptosis	RIPK1/3, MLKL	Cellular context-based modulation	^184^
NETosis	MPO, NE, PAD4	Cellular context-based modulation	^6,217^
Oxieptosis	KEAP1-PGAM5-AIFM1	Cellular context-based modulation	^216^

*PI3K/AKT* Phosphoinositide 3-Kinase/Protein Kinase B, *EGFR* Epidermal Growth Factor Receptor, *JAK/STAT* Janus Kinase/Signal Transducer and Activator of Transcription, *ERK1/2* Extracellular Signal-Regulated Kinase 1/2, *NF-kB* Nuclear Factor Kappa-Light-Chain-Enhancer of Activated B Cells, *Wnt/β-catenin* Wingless-related integration site/β-catenin, *ERK* Extracellular Signal-Regulated Kinase, *JNK* c-Jun N-terminal Kinase, *NRX* Nucleoredoxin, *MEK (1/2)* Mitogen-Activated Protein Kinase Kinase 1/2, *ASK1* Apoptosis Signal-Regulating Kinase 1, *JAK2* Janus Kinase 2, *xCT* Cystine/Glutamate Transporter (SLC7A11 subunit), *GPX4* Glutathione Peroxidase 4, *FSP1* Ferroptosis Suppressor Protein 1, *DHODH* Dihydroorotate Dehydrogenase, *GCH1* GTP Cyclohydrolase 1, *NLRP3 inflammasome* NOD-LRR- Pyrin Domain-Containing Protein 3 inflammasome, *GSDMD* Gasdermin D, *RIPK1/3* Receptor-Interacting Protein Kinase 1/3, *MLKL* Mixed Lineage Kinase Domain-Like Pseudokinase, *MPO* Myeloperoxidase, *NE* Neutrophil Elastase, *PAD4* Peptidyl Arginine Deiminase 4, *Keap1* Kelch-like ECH-Associated Protein 1, *PGAM5* Phosphoglycerate Mutase 5, *AIFM1* Apoptosis-Inducing Factor Mitochondrial 1

#### Potential targets in the ROS-responsive pathway

As PTP inhibition might modulate several oncogenic pathways, developing small molecules to activate or protect PTPs from oxidative modification offers therapeutic potential. A study observed TRXR1 to exhibit potential in protecting PTP1B from oxidative inhibition by H_2_O_2_.^406^ NRX, a redox-sensitive regulator of Dvl proteins, modulates Wnt/β-catenin signaling; its ROS-mediated inactivation promotes excessive self-renewal of hematopoietic stem/progenitor cells (HSPCs) in myelodysplastic syndrome (MDS).^407^ Although no direct activators exist, antioxidants (e.g., NAC, Ascorbic acid) and redox modulators (e.g., Ebselen, Curcumin) may influence NRX activity. Notably, as a TRX subfamily member, NRX itself supports antioxidant defense by protecting enzymes like catalase from ROS-induced oxidation, enhancing H_2_O_2_ detoxification.^408^ Furthermore, its downregulation might reduce β-catenin levels and cause a carcinogenic shift,^240^ warranting further exploration of its therapeutic potential in redox control and Wnt signaling.

Targeting JAK2 in JAK/STAT pathway might offer a promising strategy to disrupt the ROS-STAT pathway. JAK2 inhibitors such as AG490 and Ruxolitinib reverse ROS-induced STAT activation and mitigate pSTAT3-driven ICAM-1 and PD-L1 induction.^409^ Similarly, inhibiting Ras and MEK1/2 offers another anticancer therapeutic approach by blocking the ROS-ERK pathway. For example, Alendronate sodium blocks Ras prenylation to induce G1 arrest and apoptosis by inhibiting ROS-mediated ERK1/2 signaling.^410^ The MEK inhibitor PD98059 suppresses ROS-induced ERK1/2 activation, reducing glial fibrillary acidic protein (GFAP) expression (a marker of gliosis).^411^

Targeting ASK1 activation in the JNK and p38 pathways represents another potential therapeutic strategy, as various compounds can induce apoptosis through the ROS-ASK1-JNK/p38 axis. Flexicaulin triggers ROS/ASK1/JNK activation in esophageal cancer,^412^ while Quercetin induces AMPK-α, ASK1, and p38/caspase-dependent apoptosis.^413^ Daidzein and Gefitinib synergistically promote c-Jun translocation via this axis.^414^ Additionally, Catechol-containing polyphenols induce ROS via tyrosinase-mediated oxidation, triggering ASK1/JNK/p38 signaling and apoptosis in A375 melanoma cells.^415^

#### Potential targets in ROS-responsive cellular processes

The BCL-2 family and caspase family are potential therapeutic targets in ROS-mediated apoptosis.^157,158^ Targeting these molecules involves many pathways and molecules, including ER stress pathways (UPR sensors, CHOP, and calcium signaling), p53 tumor suppressors (which regulate the BAX/BCL-2 balance), mitochondrial apoptosis (cytochrome c release and caspase activation), and antioxidant systems (TRXR inhibition and GSH depletion).^44,157,158^ Therefore, therapeutic strategies involve ROS-inducing agents (prooxidant drugs, TRXR/GSH inhibitors), ER stress inducers, p53 reactivators, and combination therapies (e.g., with PARP inhibitors or immunotherapy).

The ferroptosis defense network includes multiple parallel antioxidant systems—xCT/GPX4, FSP1/CoQ10, DHODH, and GCH1/BH4—which prevent lipid peroxidation, presenting key therapeutic targets. Besides xCT/GPX4 inhibition (by Erastin/Sulfasalazine/RSL3),^379–381^ FSP1 inhibitors (iFSP1, Liproxstatin-1) and DHODH blockers (TPP-brequinar/B2, Leflunomide) disrupt organelle membranes and mitochondrial antioxidants, respectively.^416–419^ GCH1 suppression (by Gambogenic acid) amplifies ROS by depleting BH4, as evidenced in NSCLC.^420^ Notably, multitarget agents like Brequinar (DHODH/FSP1) exploit the system’s redundancy, selectively targeting cancer cells, especially in OXPHOS-dependent or immunotherapy-resistant malignancies.^421^ The NLRP3 inflammasome-GSDMD-caspase-1/NF-κB axis represents a pivotal but context-dependent target in ROS-mediated pyroptosis, but NLRP3 has dual roles in disease pathogenesis.^422^ Therefore, precision modulation is critical: inhibitors (MCC950) for inflammatory diseases versus activators (Saikosaponin-D, Baicalin) for immunogenic tumor killing.^422–424^ Additionally, emerging agents like Gambogic acid targets ROS/caspase-3/GSDME, while Shikonin, GEFT, and Paris saponin-7 modulate ROS-GSDM signaling to induce pyroptosis in several cancers.^425–428^

Targeting the Keap1/PGAM5/AIFM1 axis in ROS-mediated oxieptosis could selectively amplify oxidative stress to induce programmed cell death in cancer cells, offering a novel therapeutic strategy. In cancer cells, including CRC cells, ROS inducers like Sanguinarine (SNG) trigger Keap1-PGAM5-dependent cytotoxicity by inducing oxieptosis.^216^

RIPK1/3 and MLKL, as central mediators of necroptosis, are promising therapeutic targets in ROS-driven necroptosis.^184^ However, RIPK1 modulation is governed by phosphorylation, with activation driven by autophosphorylation at Ser161 and Ser166 and ROS-induced modifications.^429^ Conversely, inhibitory phosphorylation by TAK1, MAP kinase-activated protein kinase 2 (MAPKAPK2), IKKα/β (at Ser320/Ser321, Ser336, and Ser25), and ULK1 (at Ser357) restrains RIPK1-mediated cell death,^430^ suggesting residue-specific modulation of these targets. Additionally, agents like Acetylshikonin, RETRA, and *Erigeron breviscapus* induce RIPK1/RIPK3/MLKL-dependent necroptosis, indicating their antitumor potential.^431–433^

MPO, NE, and PAD4 are key targets in ROS-mediated NETosis. While in early-stage cancers, antitumor N1 neutrophils dominate, suggesting that NETosis induction could enhance tumor cell killing,^434^ its inhibition in advanced stages can block protumor N2 neutrophil effects. ROS scavengers like Kaempferol suppress NETosis and reduce lung metastasis in a mouse breast tumor model by targeting the ROS-PAD4 pathway.^435^

### Targeting cancer vulnerabilities via a combination approach

ROS-modulating combination therapies have become integral components of many adjuvant and neoadjuvant cancer treatment regimens, addressing the complexity and adaptability of ROS-mediated cancer cell biology. The anticancer combination therapeutic strategies leveraging ROS for anticancer effects are discussed in this section and in Table 5. The data concerning the antitumorigenic strategies have been collected from www.fda.gov, http://clinicaltrials.gov, http://go.drugbank.com, and different works of literature.

**Table 5:** List of anticancer combination therapeutic strategies leveraging ROS for anticancer effects

Combination	Compound(s)/techniques	Key findings/ROS-mediated antitumor mechanism	Current status in cancer therapy	Refs.
Coinhibition of TRXR and GLS	Auranofin (Au) + CB-839	Auranofin + CB-839 → ↑TRXR1 & glutamine→ ↑ROS → ↓Viability in Glutamine-free media & MYC-high HGSOC cells	PCS: HGSOC PDX models	^436^
Coinhibition of TRXR and GSH	Auranofin + Loss of GSR (GSH Pathway)	GSR (GSH pathway) loss + Auranofin → ↑ROS (synergistic oxidative stress) → Apoptosis → ↑Sensitivity of NSCLC cells to TRXR inhibition	PCS: NSCLC cells	^437^
Coinhibition of thioredoxin system and SOD	Auranofin + ATN-224	Auranofin + ATN-224 → ↑ROS → Resensitization to β-lapachone	PCS: A549 NSCLC cells (KEAP1^MUT^)	^438^
Coinhibition of TRXR and GSTP	Auranofin + Piperlongumine (PPL)	Au +PPL→ ↓GSTP1 → ↑ROS & nM-range ↓IC_50_ vs. Au alone	PCS: Glioblastoma stem cells (GSCs)	^439^
Coinhibition of TRX and proteasome	Auranofin + Bortezomib	Auranofin + Bortezomib → GSH depletion via ATF4/CHAC1 axis/↑ROS → Proteotoxic stress→ ER & mitochondria dilation→ Selective paraptosis in breast cancer cells with reduced toxicity risk	PCS: Breast cancer cells	^440^
TRXR inhibition and chemotherapeutic drug	Withaferin A + Sorafenib	Withaferin A + Sorafenib→ ↓TRXR1 → ↑ROS → DNA/ER stress→ Apoptosis (ATF4-dependent) → HCC tumor suppression	PCS: HCC xenograft models	^441^
TRXR inhibition and DDR targeting drugs	Auranofin + Cyst(e)inase	Auranofin + Cyst(e)inase→ 6–12× ↑ROS, 80-89% ↑ DSB, Significant ↑Cell death vs. Monotherapy	PCS: Prostate cancer cell lines	^442^
TRXR inhibition and NP-based delivery system	PL-Au@P-ZnO NRs + SDT	PL-Au@P-ZnO NRs + US → 4.5× ↑ ROS vs. Untreated, ↓Cell Viability with dose	PCS: MCF-7 cell lines	^443^
	MbO_2_@Gd-NTs +RT	MbO_2_@Gd-NTs +RT → 15.4× ↑ ROS, 11.8× ↑ DSBs, 1.2× ↑Apoptosis vs PBS + RT	PCS: Lewis lung cancer xenograft model	^444^
	CM-Coated MSNs/Ce6/Cur + PDT	CAM-MSN/Ce6/Cur → Homologous targeting → ↓TRXR1/2, ↑PDT → ↑ROS → Tumor inhibition	PCS: CAL-27 cell line	^445^
Mitochondria targeting drug and GPX inhibition	Artemisinin + RSL3	Artemisinin + RSL3 → ↓GPX4 (Weak ↓GPX4 by Artemisinin) → ↑ROS →↑Ferroptosis, Apoptosis	PCS: Breast cancer cells	^446^
Mitochondria targeting drug and NQO1 inhibition	FRV–1 + Dicoumarol	Dicoumarol + FRV-1 → D-state Complex I inhibition, ↓OXPHOS, ↓NQO → ↑mtROS → ↓ATP/Δψm → ↓Viability (vs FRV-1 alone) in NQO1^+^ cells	PCS: NQO1-positive BC cells	^447^
Mitochondria and DDR targeting drugs	Elesclomol + Rucaparib (Ru)/Talazoparib (Tala)	Ruca/Tala + Elesclomol (Synthetic lethality) → 100× ↑Elesclomol sensitivity→ ↑ROS → ↓Viability in BRCA1-mutant (↓BER) vs. Wild-type	PCS: BRCA1-mutated cancer cells	^448^
Mitochondria and ER stress targeting drugs	EMT-NPs	EMT-NPs → ER (↑IRE1α/ ↑ CHOP → ↑Ca^2+^), Mitochondria (↓MMP → ↑Cyt c) →↑ROS →↑Caspase-12/3 → Apoptosis	PCS: A549 NSCLC CDX and ovarian cancer PDX model	^449^
Mitochondria targeting SDT-proteasome inhibition	IR-820@NBs + MG-132 + SDT	IR-820@NBs + US + MG-132 (Synergistic tumor suppression) → ↓ΔΨm → ↑ROS →↑Apoptosis, ↑Autophagy → ↓Viability, ↓Migration/Invasion	PCS: HEPG2 HCC cell lines	^450^
Mitochondria targeting drug with chemotherapeutic drug	Elesclomol Sodium + Paclitaxel	Elesclomol Sodium + Paclitaxel→ ↑ROS, 2× ↑PFS, 41.7% ↓ DPR, ↑OS vs. Paclitaxel alone	CT, Phase II, Metastatic melanoma	^451^
		76% ↑ PFS (in Normal LDH), NSD vs. Paclitaxel alone (in Elevated LDH)	CT, Phase III, Metastatic melanoma	^452^
Mitochondria targeting drug and glycolysis inhibition	Elesclomol + (2-DG/3-BP)	Elesclomol + 2DG/3BP →↓Rapid ΔΨm, ↓Lactate →↑ROS → Additive ↑ cell death	PCS: MCF7 and MDA-MB-231 breast cancer cells	^453^
	EP13 + (Oxamate/2-DG)	EP13 + Oxamate/2-DG→ ↓ETC I, ↓OXPHOS, ↓Glycolysis→ ↑ROS → ↓Cell viability	PCS: MCF7 and MDA-MB-231 breast cancer cells	^454^
Mitochondria targeting drug and nanoparticle-based delivery system	CAT/CPT-TPP/PEG-Ce6 (CTC)	CTC Micelles → ↑Mitochondrial ROS → ↑ICD → ↓Tumor growth (Primary/Distant), Immune activation	PCS: Hypoxic 4T1 TNBC mouse model	^455^
	AuCu-Ce6-TPP (ACCT)	ACCT + X-ray→ ¹O_2_/OH•, ↓(HIF1α/H_2_O_2_) → ↓Hypoxia→ Cell death in 4T1 cells	PCS: Hypoxic 4T1 TNBC mouse model	^456^
	CuFe_2_O_4_- Metformin (MET)- BAY-876 (CFMB)	CFMB → ↓GLUT1/ ↓ HK, ↓OXPHOS → ATP Crisis → ↑Cu^+^/↑Fe^2+^ release → ↑OH• →Tumor death	PCS: Hypoxic 4T1 TNBC mouse model	^457^
	CeO_2−x_-MET-GOx@PDA/BSA (CPGMB)	CPGMB → ↓HK2/↓Glucose → ATP Crisis → H_2_O_2_ → O_2_ (hypoxia relief)/OH• → Tumor Ablation	PCS: 4T1 breast cancer mouse model	^458^
	TPP-GOX-CAT-PpIX (tGCP)	tGCP →↓Glucose, ↑H_2_O_2_, ↑O_2_, ↑¹O_2_→ ↑ROS →Apoptosis →Tumor Regression	PCS: 4T1 breast cancer mouse model	^459^
	NP@ESCu + anti-PD-L1	NP@ESCu + anti-PD-L1 → ROS-triggered ES/Cu release→ Cuproptosis, Immune reprogramming →Tumor regression	PCS: Bladder cancer mouse model	^460^
	TPP-T-Ce6- FX11	TPP-T-Ce6- FX11 → GSH-triggered release → ↓Glycolysis → ↑Mitochondrial ROS → ATP crisis → Tumor regression	PCS: MCF-7 breast adenocarcinoma xenograft mice model	^461^
Glutaminase inhibition and chemotherapeutic drug	CB-839 + Cabozantinib	42% ORR, 100% DCR, with 50% ORR	CT, Phase I for mRCC	^462^
	CB-839 + Azacitidine	70% CR	CT, Phase Ib/II study for high-risk MDS	^464^
Dual inhibition of glutamine metabolism	LL202 (GLS1i) + V9302 (ASCT2i	LL202 + V9302(Synergy) → ↓Glutamine uptake/metabolism → Energy stress ( ↑ AMPK), ↓Nrf2/HO-1) → ↑ROS→ ↑DNA damage → ↓Proliferation	PCS: Osimertinib-resistant H1975OR xenografts lung cancer model	^465^
Targeting glutamine metabolism and NQO1	BPTES/CB-839 + ß-Lap	BPTES/CB-839 + ß-Lap → ↓NADPH, ↑ROS → DNA damage → PARP hyperactivation → NAD⁺ depletion → Apoptosis →↓PDA cell viability (KRAS^MUT^, NQO1-high)	PCS: Pancreatic ductal adenocarcinomas (PDA) cell lines	^466^
Glutaminase inhibition and CAP/PDT	C968-Ce6 (C9SN)	C9SN → ↓GSH→ ↓ROS scavenging → ↑Oxidative stress → ↑Cell death/ICD → Neoantigen release → DC maturation → M2-to-M1 TAM polarization → CTL recruitment/activation	PCS: 4T1 breast cancer mouse model	^467^
Coinhibition of glutamine and one-carbon metabolism	BPTES + CBR-5884 (PHGDH inhibitor)	BPTES + CBR-5884 +Lenvatinib/Sorafenib (Chemo Synergy) → ↓1 C metabolism, ↓Glutaminolysis → Cell cycle arrest, ↑ROS → ↓Proliferation → Cell death	PCS: ATC cells	^468^
Glycolysis inhibition and chemotherapeutic drug	2-DG + Etoposide	2-DG + Etoposide →↓Glycolysis → ↓NADPH & ATP → ↑ ROS → ER stress → ERp57/Calreticulin exposure → Immunogenic cell death (ICD) → Tumor-specific T-cell activation → ↑Survival in immunocompetent mice	PCS: Lymphoma	^469^
Coinhibition of TRX and glycolysis	2-DG + PX-12	2-DG + PX-12 (Synergistic) → ↓TRX1, ↓Glycolysis → ↑ROS → ↑2-DG cytotoxicity (in vitro/vivo)	PCS: CRC cells	^470^
Coinhibition of glycolysis, TRX and GSH system	2-DG + Auranofin + BSO	2-DG + Auranofin + BSO → ↓TRXR, ↓GSH, ↓Glycolysis → ↑ROS → Apoptosis → ↓E-BCSCs, ↓M-BCSCs	PSC: Breast cancer stem cells	^471^
GSH depletion and chemotherapy	FeP@HCPT-HA	FeP@HCPT-HA → Release of HCPT, Fe^2+^, Fe^3+^ → ↑H_2_O_2_, ↓GSH→ ↑ROS → ↑Lipid peroxidation → Ferroptosis, Apoptosis	PCS: CD44-overexpressing tumor cells	^472^
GSH depletion and PDT	PEG(-b-PCL-Ce6)-b-PBEMA + Docetaxel (DTX) + PDT	Polymeric Micelles +DTX + PDT (Light induced) → ↓GSH → ↑ROS → 1.24-2.39× ↓ IC_50_ vs. free DTX, ↓Tumor volume vs. controls, 75% tumor necrosis, 70% apoptosis, ↓Ki-67/CD34 (In vivo)	PCS: Syngeneic 4T1 breast cancer mouse model	^473^
GSH depletion and GLUT inhibition	FCSP@876 MOFs	FCSP@876 MOFs → ↑SLC7A11, ↑Cystine, ↓GLUT1→ ↓NADPH → ↓Cysteine, ↓GSH → ↓GPX4 → ↑ROS → Ferroptosis, Disulfidptosis → ↑Cell death (↓Ki67, ↓GPX4, ↑PTGS2)	PCS: 4T1 breast cancer mouse model	^474^
GPX inhibition and chemotherapeutic drugs	GPX4 inhibition + BRAFi ± EGFRi	Targeting GPX4 + BRAFi ± EGFRi → ↓ GPX4 expression→ ↑ROS→ ↑Lipid peroxidation → Ferroptosis → Restored sensitivity to BRAFi ± EGFRi	PCS: BRAFV600E colorectal cancer	^475^
Coinhibition of TRXR and GPX	Jolkinolide B (JB) + GPX4i (RSL3/ML162)	Jolkinolide B + RSL3/ML162 → ↓GPX4 → ↓TRXR1 overexpression (GPX4i induced) → ↑Lipid ROS → Ferroptosis/Apoptosis→ ↓GPX4i resistance (↑IC50) in Cisplatin-resistant bladder cancer cells	PCS: Cisplatin-resistant bladder cancer cells	^476^
GPX inhibition and PDT/PTT	Composite hydrogel (ZCND-Erastin/PAA: F127)	ZCND + Erastin→ ↓HSP70 → ↓GPX4 → ↑ROS → Synergistic ferroptotic cell death	PCS: 4T1 breast cancer mouse model	^477^
Coinhibition of GPX and Nrf2	Nrf2i (TRI/CP/ML385) + GPX4i (RSL3/ML210)	ML385 + RSL3/ML210 → ↓Nrf2/GPX4 → ↑Lipid ROS → Ferroptosis/Apoptosis→ ↓Tumor growth, ↓Metastasis, ↓Ascites (no toxicity) vs. Vehicle/RSL3 alone (in vivo)	PCS: ID8-Luc ovarian cancer model	^478^
GSTP inhibition and GSH depletion via NP delivery	EA-SS-CHL (ECPP)	ECPP → ↓GSH/GSTP-pi→ ↑ROS→ Oxidative stress → Apoptosis	PCS: 4T1 breast cancer mouse model	^479^
Proteasome inhibition and chemotherapeutic drug	Bortezomib (BTZ) + Actinomycin D (ActD)	BTZ + ActD (Synergistic) → ↓Proteasome activity upregulation by ActD→ Proteotoxic stress → Impaired cell-cycle progression → Apoptosis → ↑ActD sensitivity	PCS: Wilms tumor cells	^480^
Proteosome inhibition and CDT	MIL-88-MG132@M	MIL88-MG132@M → UPS blockade → ↑Ubiquitinated proteins→ ↓NF-κB/↑(p53-Ser536) → ↑ROS → ↑Apoptosis → ↓Tumor volume, ↓Cyclin D1/NF-κB, ↑Ubiquitin/p53, ↑DC maturation, ↑CTLs vs. Monotherapy (in vivo)	PCS: CT-26 CRC-bearing mice model	^481^
Dual inhibition of ubiquitin–proteasome pathway	Carfilzomib (CFZ) + Bortezomib (BZ)	BZ + CFZ (Synergistic) → ↓Proteasome → ↑UPA → ↑ER stress/ROS → ↓MMP → ↑Caspase-3/8/9/12 → Apoptosis → 56.4% ↓Tumor vs. Monotherapy (in vivo)	PCS: C57BL/6 melanoma xenograft model (mouse)	^482^
Coinhibition of glutaminase and proteasome	V9302 + Carfilzomib	V9302 + Carfilzomib→ ↓Glutamine metabolism (↓MYC/NRAS/GLS), ↓Proteasome → ↑ER stress, ↑ROS → Apoptosis (↑Caspase-3/PARP), Autophagy (↑LC3-II)	PCS: PCM cell lines	^483^
Proteosome inhibition and Immune therapy	IL-33+ Bortezomib (BTZ)	IL-33 + BTZ(Synergistic) → ↓GSH → ↑ ROS→ ↓NF-κB-p65 nuclear translocation→ ↓Stemness (SOX2/MYC/OCT4) → ↑Apoptosis → ↓Tumor growth vs. BTZ alone	PCS: U226B1 MM cell xenograft mouse model	^485^
Dual inhibition of GSTP and NQO1	MNPC (Inhibits NQO1 & GSTP1)	MNPC → ↓GSH/GSSG → ↑ROS → Oxidative stress → Caspase-3 activation → Apoptosis, LDH release → ↓Tumor volume, ↑Survival	PCS: Glioblastoma U87MG-EGFRvIII xenografts	^486^
Simultaneous targeting of NQO1 and SOD	IB-DNQ + ATN-224	IB-DNQ + ATN224/KD → NQO1 futile cycling, ↓SOD1 → ↓O₂•− detox → 4×↑mtROS → ↓ΔΨm→ ↓ATP→ Cyt c release → ↑Caspase-3 → Apoptosis → ↓Tumor, ↓Metastasis vs. IB-DNQ (in vivo)	PCS: TNBC xenograft models	^487^
NQO1 inhibition and chemotherapy	Dicoumarol + Chemotherapeutic Drug	Dicoumarol + Chemotherapeutic drug→ ↓ (DPP9 overexpression induced NQO1) → ↑ROS → Restored chemosensitivity	PCS: Liver cancer cells	^107^
NQO1 exploitation and CDT	NV-IONP-Lapa	NV-IONP-Lapa → NQO1 catalysis, ↓GSH → Lapa → H₂O₂ → OH• (Fenton)→ ↑Oxidative stress→ Tumor apoptosis	PCS: Ovarian cancer cells	^488^
NQO1 exploitation and DDR targeting drugs	β-Lapachone + T2AA	β-Lapachone + T2AA → ↓NQO1, ↓PCNA, ↓PARP1 hyperactivation (induced by β-Lapachone alone) →↑ DSBs, 5x↑H₂O₂ → ↑γ-H2AX foci→ ↓NAD^+^/ATP→ Necrosis → ↑Cell death vs. β-Lapachone	PCS: LLC xenograft	^489^
	β-Lapachone + Rucaparib	β-Lapachone + Rucaparib→ ↓NQO1, ↓PARP1 hyperactivation (induced by β-Lapachone alone) →↑DSBs,↑H₂O₂ → ↑γ-H2AX foci → ↓NAD⁺/ATP loss → (Necrosis → Apoptosis)	PCS: Pancreatic and NSCLC PDX models	^490^
Nrf2 inhibition and chemotherapeutic drug	Tangeretin + Paclitaxel/AZD9291 (TKI)	Tangeretin + Paclitaxel/AZD9291→ ↓Nrf2 (upregulated in resistant cells) →↑ROS → ↓P-gp → Chemo/TKI sensitization→ ↓Tumor growth	PCS: A549/T lung cancer cells-derived xenografts	^491^
Nrf2 inhibition + PDT/PTT	Brusatol/silica@MnO_2_/Ce6@PDA-PEG-FA	Brusatol/silica@MnO_2_/Ce6@PDA-PEG-FA → ↓HSPs, ↓Nrf2, ↑ROS→ ↓GPX4/FTH → Ferroptosis→ Hypoxia- and hyperthermia-associated resistance in phototherapy	PCS: Pancreatic cancer cells	^492^
DDR targeting drugs and ferroptosis induction	PARPi (Olaparib) +Ferroptosis Inducers (Sulfasalazine)	Sulfasalazine + Olaparib→ ↓PARP1, ↑p53, ↓SLC7A11 → ↓Cystine/GSH→ Lipid Peroxidation → Ferroptosis → ↓BRCA wild-type tumors	PCS: BRCA-wild-type ovarian cancer cells	^493^
DDR targeting drugs and PDT	ZTN@COF@Poloxamer–PDT/PARPi (Niraparib)	ZTN@COF@Poloxamer–PDT/(Niraparib) (Synthetic lethality) → ↑ROS → DNA damage (&DNA repair inhibition) → Tumor apoptosis, Immune activation → ↓Metastasis	PCS: Soft tissue sarcoma cells	^494^
NOX activation and chemotherapeutic drug	Berbamine Hydrochloride (Ber) + Cisplatin	Ber+ Cisplatin→ ↑NOX2 → Lysosomal alkalinization → Autophagy blockade (↑LC3-II/p62) → ↑ROS → MAPK activation → Apoptosis → ↑Cancer cell death	PCS: Lung adenocarcinoma xenograft mouse model	^496^
Heat shock protein inhibition and chemotherapeutic drug	Tubeimoside-I (TBM) + Oxaliplatin	TBM + Oxaliplatin (Synergy)→ ↓HSPD1 → ↑ROS → ER stress, JNK/p38 activation → Apoptosis → ↓Tumor growth vs. Monotherapy	PCS: CRC xenograft mouse model	^498^
Ferroptosis induction and PDT	TBzT-CNQi + FSP1 inhibitor (iFSP1)	TBzT-CNQi (membrane-targeting) + iFSP1→ ↑ROS, ↓FSP1 → ↓CoQ10 → ↑LPO → Immunogenic ferroptosis → ↑ICD → DC maturation, CD8^+^ T-cell recruitment → Tumor clearance	PCS: 4T1 breast cancer mouse model	^499^
IDO1 inhibition and chemotherapeutic drug	PTX@PoxMTP NPs	PTX@PoxMTP NPs →1.5× ↑ROS, ↓IC50, 1.26×↑Apoptosis, 1.2× G2/M arrest, 1.1–1.5× ↑ HMGB1/ATP, 1.25–1.5×↑DCs maturation, ↓IDO1/kyn → Immune activation, ↓Tumor growth, ↓lung metastases, 3.7× ↑ CD8^+^ T cells, ↓Tregs/M2-TAMs, and ↑CD8+/Treg ratio vs. Free PTX	PCS: 4T1 breast cancer bearing mice model with lung metastasis	^500^
Chemotherapeutic combination	Sodium arsenite (NaAsO₂) + Chloroquine (CQ) + Dichloroacetate (DCA)	NaAsO₂ + DCA + CQ → ↓Glycolysis (↓LDHA) → ↓Autophagy (↑LC3B by NaAsO₂, blocked by CQ) → ↑CAT, ↑SOD → ↓Lipid peroxidation (↓MDA) → ↓Tumor growth→ ↑Survival: 90% (combo) vs. 35% (control)	PCS: 4NQO-induced OSCC mice model	^501^
Lipid metabolic reprogramming and immune checkpoint blockade	Lipofermata + Anti-PD-L1	Lipofermata + Anti-PD-L1→ ↓FATP2 → ↓Lipid accumulation & ROS in MDSCs→ ↓Immunosuppression → ↑T-cell activation (↑CD107a, ↑IFN-γ) → ↓PD-L1 expression → ↑Tumor clearance	PCS: LLC xenograft Mouse model	^502^
Antioxidant and chemotherapeutic drug	Curcumin + Paclitaxel	Curcumin + Paclitaxel → 60% ↑ ORR vs. Placebo	CT, Phase II	^503^

*TRX* Thioredoxin, *TRXR* Thioredoxin Reductase, *GLS* Glutaminase, *SOD* Superoxide Dismutase, *GSH* Glutathione, *GCLC* Glutamate-Cysteine Ligase Catalytic Subunit, *GSTP* Glutathione-S-Transferase, *PCS* Pre-clinical Study, *CT* Clinical Trial, *GPX* Glutathione Peroxidase, *HGSOC* High-Grade Serous Ovarian Cancer, *DMSO* Dimethyl Sulfoxide, *PI* proteosome Inhibitors, *BTZ* Bortezomib, *IXZ* Ixazomib, *DLZ* Delanzomib, *CRC* Colorectal Cancer, *HCC* Hepatocellular Carcinoma, *NSCLC* Non-Small Cell Lung Cancer, *GSC* Glioma Stem Cell, *ATF4* Activating Transcription Factor 4, *DSB* Double-Strand Break, *RT* Radiotherapy, *Gy* Gray (unit of radiation dose), *PBS* Phosphate-Buffered Saline, *MbO*_2_*@Gd-NTs* Oxygenated Myoglobin-Loaded Gadolinium Nanotubes, *CM-Coated MSNs/Ce6/Cur CAL-27* Cancer Cell Membrane-Coated Mesoporous Silica Nanoparticles Co-Loaded with Chlorin e6 and Curcumin, *PL-Au@P-ZnO NRs* Piperlongumine-loaded, Au-decorated, poly(ethylene glycol)-coated zinc oxide nanorods, *SDT* Sonodynamic Therapy, *Us* Ultrasound, *PFS* Progression-Free Survival, *OS* Overall Survival, *DPR* Durable Prostate-specific antigen (PSA) Response, *OXPHOS* Oxidative Phosphorylation, *ETC* Electron Transport Chain, *CPT* Chemo-Photo Therapy, *HIF1α* Hypoxia-Inducible Factor 1-Alpha, *GLUT1* Glucose Transporter 1, *TPP-GOX-CAT-PpIX* Triphenylphosphine-Conjugated Glucose Oxidase/Catalase/Protoporphyrin IX Hybrid System, *NP@ESCu* Nanoparticles with Elesclomol and Copper, *TPP-T-Ce6- FX11* Triphenylphosphonium-Conjugated Chlorin e6 (T-Ce6) Co-Delivered with FX11, *EMT-NPs* Endoplasmic Reticulum (ER) and Mitochondria Dual-Targeting Nanoparticles, *MMP* Mitochondrial Membrane Potential, *BER* Base Excision Repair, *PHGDH* Phosphoglycerate Dehydrogenase, *ASCT2* Alanine-Serine-Cysteine Transporter 2, *PKM2* Pyruvate Kinase M2, *AIF* Apoptosis-Inducing Factor, *AMPK* AMP-Activated Protein Kinase, *MYC* MYC Proto-Oncogene, *NRAS* Neuroblastoma RAS Viral Oncogene Homolog, *DCR* Disease Control Rate, *CR* Complete Response, *CAP* Cold Atmospheric Plasma, *CTL* Cytotoxic T Lymphocyte, *EA-SS-CHL* Ethacrynic Acid Disulfide-Conjugated Chlorambucil (Prodrug), *NV-IONP-Lapa* Nanovesicle-Encapsulated Iron Oxide Nanoparticles Loaded with β-Lapachone, *FCSP@876 MOFs* Fe-Cu-SS metal–organic frameworks (MOFs) loaded with BAY876, *GLUT* Glucose Transporter, *PSTG2* Prostaglandin-Endoperoxide Synthase 2, *PCNA* Proliferating cell nuclear antigen, *T2AA* T2 amino alcohol, *SOX2* SRY-Box Transcription Factor 2, *OCT4* Octamer-Binding Transcription Factor 4, *MIL-88-MG132@M* Macrophage Membrane-Coated-MG132-Loaded-NH₂-MIL-88(Fe)-Metal-Organic Framework, *DHA* Docosahexaenoic Acid, *NIR* Near-Infrared, *TKI* Tyrosine Kinase Inhibitor, *MDR* Multidrug Resistance, *Brusatol/silica@MnO*_2_/*Ce6@PDA-PEG-FA* Brusatol-loaded silica nanonetwork coated with manganese dioxide and chlorin e6, further functionalized with polydopamine-polyethylene glycol-folate, *MAPK* Mitogen-Activated Protein Kinase, *ICG* Indocyanine Green, *HSPD1* Heat Shock Protein Family D (Hsp60) Member 1, *CDT* Chemodynamic Therapy, *RDT* Radiodynamic Therapy, *PDT* Photodynamic Therapy, *MET* Metformin, *GNP* Gold nanoparticles, *PTT* Photothermal Therapy, *ΔΨm* Mitochondrial depolarization, *↓* inhibition/depletion, *↑* activation/release, *NV* extracellular vesicle (EV) mimetic nanovesicles, *IONPs* Iron oxide nanoparticles, *Lapa* β-Lapachone, *GM-CSF* Granulocyte-Macrophage Colony-Stimulating Factor, *STAT3* Signal Transducer and Activator of Transcription 3, *FATP2* Fatty Acid Transport Protein 2, *MDSC* Myeloid-Derived Suppressor Cell, *PTX@PoxMTP NPs* Paclitaxel-loaded-ROS-responsive-1-MT-conjugated-PEGylated polyoxalate nanoparticles, *PEG* Polyethylene glycol, HMGB1 High Mobility Group Box 1, *ATP* Adenosine Triphosphate, *IFN* Interferon, *TAM* Tumor-Associated Macrophages, *IDO1* Indoleamine 2,3-Dioxygenase 1, *CRT* Calreticulin, MDA Malondialdehyde, *MTT* 3-(4,5-Dimethylthiazol-2-yl)-2,5-Diphenyltetrazolium Bromide, *CAT* Catalase, *LDH* Lactate Dehydrogenase, *LC3B* Microtubule-Associated Protein 1A/1B-Light Chain 3B, *LPO* Lipid Peroxidation, *ICD* Immunogenic Cell Death, *FSP1* Ferroptosis Suppressor Protein 1, *DC* Dendritic Cell, *PARP1* Poly(ADP-Ribose) Polymerase 1, *ZTN@COF@poloxamer* Zirconium-TCP NanoMOF@Covalent Organic Framework@Poloxamer Nanocapsules (where “TCP” Tetrakis(4-carboxyphenyl)porphyrin), *SLC7A11* Solute Carrier Family 7 Member 11, *BRCA* Breast CAncer gene (BRCA1/BRCA2)

#### Combination therapeutic strategies targeting ROS elevation

##### TRX system in combination therapy

TRX inhibition often drives cancer cells to alternative metabolic pathways.^436^ For example, in MYC-high HGSOC, TRX inhibition shifts cells to glutamine metabolism, and combining Auranofin with CB-839 exacerbates ROS accumulation and induces metabolic stress and apoptosis.^436^ In NSCLC, the complementary roles of the TRX and GSH antioxidant systems are also evident, as simultaneous inhibition—via Auranofin treatment combined with GSR (GSH pathway) loss—synergistically elevates ROS levels, overwhelms cellular redox defenses, and triggers apoptosis, thereby sensitizing cancer cells to TRXR inhibition.^437^ Additionally, in Nrf2-activated NSCLC with Keap1 mutations, resistance to β-lapachone arises from enhanced antioxidant defenses. Targeting the TRX system and SOD1 has been found to disrupt redox balance synergistically, amplifying β-lapachone-induced ROS, DNA damage, and cell death.^438^

In glioblastoma, TRX inhibition by Auranofin upregulates GSTP1 as compensation, while Piperlongumine (PL) directly inhibits GSTP1, disabling the GSH system. Dual inhibition disrupts redox homeostasis, amplifies ROS, and lowers the IC50 to nanomolar levels, highlighting their potential for repurposing in glioblastoma therapy.^439^ A recent study suggested that simultaneous inhibition of the TRX system (Auranofin) and the proteasome (Bortezomib) synergistically induces paraptosis in breast cancer cells by promoting GSH depletion through the ATF4/CHAC1 axis, leading to proteotoxic stress and characteristic dilation of the ER and mitochondria.^440^ This combination selectively targets cancer cells at lower doses, offering an effective, less toxic strategy against apoptosis-resistant cancers. Inhibition of the TRX system has also been shown to effectively enhance chemosensitivity through ROS-dependent mechanisms. In HCC, Withaferin A synergizes Sorafenib to promote ROS-mediated ER stress, DNA damage, and apoptosis.^441^ Targeting both the TRX system and the DDR has demonstrated promising outcomes across several cancers. A study demonstrated that Cyst(e)inase, an enzyme depleting L-cysteine/cystine, when combined with Auranofin, amplified ROS levels and DSBs, inhibiting prostate cancer growth.^442^

##### Nanoparticle-driven combination therapies targeting the TRX system

TRXR inhibitors, including GNPs, Auranofin, CONPs, Curcumin derivatives, and others, are utilized as sensitizers in RT, PDT, CDT, and ultrasound (US) irradiation via nanoparticle-based delivery. This approach enhances treatment efficacy, overcomes resistance, and reduces required doses, minimizing side effects.

Gold nanoparticles (GNPs) act as nanoradiosensitizers, enhancing localized energy deposition and ROS generation, leading to DNA damage. A novel chemopiezocatalytic therapy using Piperlongumine (PL)-loaded, Auranofin (Au)-decorated, PEG-coated zinc oxide nanorods (PL-Au@P-ZnO NRs) leverages the piezoelectric properties of ZnO under US to generate ROS.^443^ Auranofin enhances ROS via Fenton-like activity, and PL increases specificity, inducing selective tumor cell death. In vivo, PL-Au@P-ZnO NRs with US suppress tumor growth without causing systemic toxicity.^443^

Motexafin Gadolinium (Xcytrin®), a Gadolinium (Gd^3+^)-coordinated Texaphyrin (Gd-Tex) radiosensitizer, has been investigated in clinical trials for glioblastoma multiforme and pancreatic cancer, but the outcomes remain unpublished. Limited efficacy has led to the development of Gd-Tex-lipid-based nanovesicles (Gd-NTs) for improved tumor accumulation and radiosensitization.^444^ Incorporating O_2_-dependent myoglobin (Mb) into Gd-NTs (MbO₂@Gd-NTs) alleviates tumor hypoxia, amplifies Gd-Tex-induced ROS production, enhances radiosensitization, and induces long-term antitumor immune memory.^444^

Curcumin is being evaluated as a radiosensitizer in prostate (NCT01917890) and cervical cancer (NCT05947513) but faces challenges due to its low bioavailability, short half-life, and HIFs, necessitating nanoparticle-based delivery systems. Preclinically, a biomimetic nanoplatform using mesoporous silica nanoparticles (MSNs) loaded with Chlorin e6 (Ce6) and Curcumin (Cur) and coated with cancer cell membranes (CMs) demonstrated enhanced tumor targeting and uptake in oral carcinoma.^445^ Cur inhibited TRXR1, disrupting ROS defenses and amplifying Ce6-mediated ROS under laser irradiation, significantly inhibiting tumor growth.

##### Targeting mitochondria in combination therapy

Combining mitochondria-targeting drugs with ROS-modulating therapies effectively overcomes cancer cell metabolic plasticity and enhances efficacy through synergistic pathways compared to monotherapy. For instance, Artemisinin generates ROS and induces apoptosis while weakly inhibiting GPX4 to trigger mild ferroptosis. Combining Artemisinin with GPX4 inhibitor, RSL3 synergistically enhances ferroptosis, highlighting the potential of this combination for effective cancer therapy.^446^ Targeting mitochondria and NQO1 may overcome drug resistance in NQO1-overexpressing BC. FRV-1 targets ETC-I, inducing ROS and mitochondrial dysfunction in NQO1-negative cells but is inactivated in NQO1-positive MCF7 cells. Combining FRV-1 with the NQO1 inhibitor Dicoumarol has been reported to restore sensitivity.^447^

Mitochondria-DDR targeting combinations show synergistic lethality in BRCA1-mutated cancers. Defective base-excision repair (BER) sensitizes cells to mito-ROS damage, making Elesclomol effective; moreover, combining Elesclomolwith PARP inhibitors, Rucaparib or Talazoparib amplifies ROS-induced DNA damage and enhances synthetic lethality.^448^ Targeting the ER and mitochondria have demonstrated anticancer efficacy in ovarian cancer. Dual-targeting nanoparticles (EMT-NPs), which synergistically induce ER stress and mitochondrial dysfunction by promoting Ca^2+^ efflux, ROS production, and apoptosis, have recently been developed.^449^ They also enhance imaging in xenograft models, offering a drug-free, precision cancer-related prognostic strategy. Finally, to overcome the limitations of IR-820, IR-820@NBs were developed, which target mitochondria under ultrasound to generate lethal ROS,^450^ while in combination with proteasome inhibitors, MG-132 further enhances SDT efficacy by inducing apoptosis and autophagy in HCC cells.

Targeting mitochondria has shown promise in enhancing chemosensitivity. The Elesclomol–Paclitaxel combination exploits Elesclomol’s ROS generation and mitochondrial apoptosis induction capacity to sensitize cancer cells to Paclitaxel. This combination has been evaluated in several clinical trials. A Phase II trial in patients with metastatic melanoma revealed that compared with Paclitaxel alone, Elesclomol-Paclitaxel doubled the median PFS, reduced DPR by 41.7%, and improved OS.^451^ However, a subsequent Phase III trial (NCT00522834) in chemotherapy-naive advanced melanoma patients failed to show efficacy overall, although patients with normal lactate dehydrogenase (LDH) levels responded better.^452^ These findings suggest the reliance of Elesclomol on OXPHOS, with the use of glycolysis inhibitors potentially enhancing its efficacy in high-LDH tumors. In support of this, a study in breast adenocarcinoma cells (MCF7 and MDA-MB-231) demonstrated greater cytotoxicity with Elesclomol and glycolysis inhibitors (2-DG or 3-BP) compared to single-agent therapies.^453^ Similarly, EP13, an OXPHOS inhibitor targeting complex I, increased ROS levels and synergized with glycolysis inhibitors (Oxamate and 2-DG) in aggressive breast cancers.^454^

##### Nanoparticle-driven combination therapies targeting mitochondria

Recent nanotechnology advances integrate mitochondria-targeted therapeutics with multimodal strategies to address hypoxia and enhance efficacy. A polymer micelle (CAT/CPT-TPP/PEG-Ce6, CTC) achieves triple-synergistic amplification of ROS via PDT and chemotherapy, inducing immunogenic cell death.^455^ Another nanoparticle system, AuCu-Ce6-TPP (ACCT), combines RT, RDT, and CDT to combat hypoxia by integrating Ce6 for sensitization, Curcumin (Cu) for OH• generation and hypoxia alleviation, Auranofin (Au) for glutathione depletion, and triphenylphosphine (TPP) for mitochondrial targeting, demonstrating potent anticancer effects in hypoxic 4T1 cells.^456^

The CFMB nanoplatform, loaded with Metformin (MET) and BAY-876 (BAY) on CuFe₂O₄ (CF), targets glycolysis and OXPHOS, depleting tumor energy via MET-mediated HK2 inhibition and mitochondrial disruption, and BAY-mediated GLUT1 inhibition. In high-GSH environments, CFMB releases Cu^+^/Fe^2+^, catalyzing H_2_O_2_ into OH• to enhance CDT, with NIR light boosting ROS production and enabling PTT for tumor suppression without toxicity.^457^ Similarly, the CPGMB nanoplatform, composed of polydopamine-coated CeO_2_-x nanorods co-loaded with MET and GOx, inhibits glycolysis via HK2 inhibition and glucose depletion. GOx-generated H_2_O_2_ is converted to O_2_ by Ce^4+^, alleviating hypoxia, while Ce^3+^ transforms H_2_O_2_ into OH• for CDT.^458^ Another study developed a mitochondriona-targeted bioreactor using TPP, GOx, CAT, and protoporphyrin IX (PpIX) that integrates glucose (starvation therapy), oxygen generation, and PDT to amplify ROS production, inducing mitochondrial destruction and apoptosis with strong in vivo antitumor efficacy.^459^

To address copper (Cu) ionophore limitations, a ROS-sensitive polymer (PHPM) was developed to coencapsulate Elesclomol (ES) and Cu, forming NP@ESCu nanoparticles.^460^ Upon ROS-triggered release in cancer cells, ES and Cu synergistically amplify ROS, inducing cuproptosis and stimulating immune responses. In a bladder cancer model, NP@ESCu reprogrammed the tumor microenvironment and, when combined with anti-PD-L1, enhanced therapeutic efficacy.^460^ Another bioreducible exosome with redox-cleavable diselenide linkers delivers mitochondria-targeting sonosensitizers (T-Ce6) and glycolysis inhibitors (FX11) to tumors.^461^ Under US irradiation, T-Ce6 induces ROS-mediated mitochondrial damage, whereas FX11 inhibits glycolysis, amplifying antitumor effects of SDT.

##### Glutaminase inhibition in combination therapy

Glutaminase inhibition in combination with other ROS modulation pathways has been shown to be effective in overcoming therapeutic resistance in various cancers. In metastatic RCC, CB-839 plus Cabozantinib achieved a 42% ORR and 100% DCR (NCT02071862),^462^ with Phase II trials ongoing (NCT0342821). For PIK3CA-mutant CRC, CB-839 combined with Capecitabine improved PFS, even in fluoropyrimidine-resistant patients (NCT02861300).^463^ In high-risk MDS, CB-839 and Azacitidine achieved a 70% marrow complete response in a Phase Ib/II study (NCT03047993).^464^ Overcoming Osimertinib resistance in NSCLC involves targeting glutamine metabolism, as resistant cells are glutamine dependent. Dual inhibition of ASCT2 and GLS1 has been shown to outperform GLS1 inhibition alone, effectively combating resistance in preclinical models.^465^ Additionally, high NQO1 expression in resistant cells supports redox homeostasis, contributing to resistance. Combining NQO1 targeting with glutamine metabolism inhibition offers a promising strategy to overcome resistance.^466^ Moreover, a nanobooster, C9SN, comprising the glutaminase inhibitor C968 and the photosensitizer Chlorin e6, enhanced PDT efficacy by depleting GSH and inducing oxidative stress.^467^ It triggered immunogenic cell death, CTL activation, and macrophage polarization, suppressing tumors and remodeling the tumor microenvironment. In anaplastic thyroid cancer (ATC), glutaminolysis inhibition alone induces growth arrest but not cell death due to compensatory ATF4-mediated one-carbon metabolism, which is amplified during progression from papillary to anaplastic thyroid cancer. Dual targeting of PHGDH, a key enzyme in one-carbon metabolism, and glutamine metabolism synergistically increases ROS, inducing growth arrest and sensitizing ATC cells to anticancer drugs.^468^

##### Glycolysis inhibition in combination therapy

Combining glycolysis inhibition with oxidative stress modulation enhances therapy by sensitizing cancer cells to ROS-mediated damage and addressing metabolic reprogramming, resistance, and nonselectivity. For instance, 2-DG synergizes with Etoposide to increase cytotoxicity, promote tumor-specific T-cell activation, and induce immunogenic cell death.^469^ Clinically, a Phase I study (NCT00247403) explored 2-DG as a radiosensitizer for intracranial neoplasms but was withdrawn due to discontinuation of drug manufacturing. This combination disrupts mitochondrial integrity, induces ROS-mediated apoptosis, and suppresses the expression of EMT markers, offering a promising metabolic reprogramming strategy for BC treatment. Glycolysis inhibition in CRC is limited by systemic toxicity and resistance via TRX1 upregulation, which enhances SLC1A5 expression, glutamine transport, and GSH synthesis to counteract cytotoxicity.^470^ TRX1 inhibitor PX-12 or SLC1A5 knockdown synergistically enhances the cytotoxic effects of 2-DG by disrupting redox balance in vitro and in vivo.^470^ Moreover, while single treatments showed minimal efficacy, coinhibition of glycolysis (2-DG), TRX (Auranofin), and GSH (BSO) synergistically suppressed tumor growth, metastasis, and tumor-initiating capacity in TNBC patient-derived xenografts by inducing ROS-mediated apoptosis in both epithelial and mesenchymal breast cancer stem cells.^471^

##### GSH depletion in combination therapy

GSH depletion enhances cancer therapy sensitivity. For example, the limited endogenous H_2_O_2_ and elevated GSH levels in tumor cells reduce the effectiveness of the iron-based nanocarrier 10-hydroxycamptothecin (HCPT) in inducing ferroptosis.^472^ To overcome this, a ferric phosphate nanotherapeutic system, FeP@HCPT-HA, was developed. FeP@HCPT-HA targets CD44-overexpressing tumor cells and degrades in the acidic tumor microenvironment to release HCPT, Fe^2+^, and Fe^3+^. The released Fe^3+^ depletes GSH, downregulates GPX4, and enhances lipid peroxidation, whereas HCPT induces apoptosis and supplies H_2_O_2_ for the Fe^2+^-mediated Fenton reaction. These synergistic mechanisms effectively induce ferroptosis and apoptosis, significantly inhibiting tumor growth. Additionally, to enhance PDT, a study designed a star-shaped polymer, PEG(-b-PCL-Ce6)-b-PBEMA, that boosts ROS generation, depletes GSH, and delivers chemotherapy. It combines PDT with Ce6 for ROS production, an H_2_O_2_-labile group for GSH depletion, and Doxorubicin delivery. This triple strategy significantly increased antitumor efficacy both in vitro and in vivo, suggesting a promising approach to amplify oxidative stress in cancer treatment.^473^ A Phase I trial (NCT00327288) evaluated Imexon-Docetaxel across various cancers to determine maximum tolerated dose, but the clinical outcomes are pending. Targeting GSH and glucose transporters also exploits the metabolic dependencies of cancer cells, suggesting a promising therapeutic approach. Cancers resist ferroptosis by upregulating SLC7A11 and increasing cysteine uptake and GSH synthesis. Glucose restriction causes cysteine accumulation and NADPH deficiency, triggering disulfidptosis. To exploit this vulnerability, FCSP@876 MOFs were developed, combining a ferroptosis-inducing MOF with the GLUT1 inhibitor BAY876. This nanoplatform induces two-pronged ROS attack: first, ferroptosis via lipid peroxidation, and second, disulfidptosis via glucose starvation-induced NADPH deficiency.^474^

##### GPX inhibition in combination therapy

GPX4 inhibition overcomes therapeutic resistance, as evidenced in BRAFV600E colorectal cancer. GPX4 upregulation in response to BRAFi ± EGFRi therapy blocks therapy-induced ferroptosis and drives resistance. Targeting GPX4 restores ferroptosis, enhances the efficacy of BRAFi ± EGFRi, and overcomes resistance in in vitro, in vivo, organoid, and patient-derived xenograft models.^475^ GPX-TRXR inhibition also overcomes cisplatin resistance in bladder cancer.^476^ Combining the TRXR1 inhibitors jolkinolide B or Auranofin, with GPX4 inhibitors offers a promising strategy for overcoming cisplatin resistance.^476^ Additionally, GPX inhibition may enhance PDT and PTT. To effectively eliminate residual tumor cells, a composite ZCND-Erastin/PAA:F127 hydrogel was developed, incorporating zinc-centered carbon nano-dodecahedrons (ZCND) and Erastin.^477^ ZCND PDT/PTT generates ROS, which reduce HSP70, destabilizing GPX4 and promoting ferroptosis, while Erastin also destabilizes GPX4 to enhance ferroptosis. In vitro and in vivo studies confirmed that this hydrogel effectively suppressed tumor recurrence after surgical resection. Coinhibition of GPX and Nrf2 also has a synergistic effect on ovarian cancer, which is particularly susceptible to ferroptosis due to its “iron addiction.” A study showed that combining Nrf2 inhibitors (e.g., ML385) with GPX4 inhibitors synergistically suppressed ovarian cancer by inducing ROS accumulation, lipid peroxidation, and caspase-3 activation, promoting ferroptosis and apoptosis.^478^ Coinhibition of GSTP and GSH enhances chemotherapy, as observed in a study on breast cancer, as Chlorambucil’s (CHL) efficacy is limited by GSH- and GSTP-pi-mediated resistance. A study developed a GSH-responsive prodrug (EA-SS-CHL) encapsulated in nanoparticles (ECPPs) that releases ethacrynic acid (EA) and CHL to deplete GSH, inhibit GSTP-pi, and amplify ROS, enhancing CHL’s cytotoxicity.^479^

##### Proteasome inhibition in combination therapy

Bortezomib has been shown to increase the efficacy of Actinomycin D in Wilms tumor cells by disrupting protein homeostasis and restoring apoptosis.^480^ Additionally, a Phase II trial (NCT01769209) explored the ability of Bortezomib to induce ROS in ALL blast cells and its potential to improve chemotherapy outcomes; the findings remain unpublished. Proteosome inhibition sensitizes CDT by elevating ROS levels and, with a macrophage membrane coating, ensures targeted tumor accumulation, as evidenced in a study on mCRC. This study used the MIL-88-MG132@M nanoplatform to overcome TME-induced CDT resistance.^481^ This Fe-MOF-based system, loaded with MG132, inhibited proteasome activity and NF-κB p65 phosphorylation, leading to the accumulation of proapoptotic proteins and the disruption of tumor homeostasis. Dual proteasome inhibition with Carfilzomib and Bortezomib has also demonstrated anticancer activity in melanoma while minimizing toxicity. A study showed that the low-dose Carfilzomib-Bortezomib combination amplifies antitumor effects through ROS-dependent apoptosis and ER stress, reducing Bortezomib-related toxicity.^482^

Co-inhibition of Glutaminase and proteasome might overcome Proteasome inhibitors (PIs) resistance, as evidenced in plasma cell myeloma (PCM). Combining the ASCT2 inhibitor V9302 with Carfilzomib synergistically elevated ROS levels, induced apoptosis, and activated a catastrophic UPR characterized by the upregulation of spliced Xbp1, ATF3, and CHOP, enhancing cytotoxicity in both PI-sensitive and resistant PCM cells.^483^ Coinhibition of the proteasome and Nrf2 can overcome proteasome inhibitor resistance by blocking the Nrf2-mediated antioxidant defense activated upon proteasome inhibition. For example, CuONPs inhibit proteasomes in vascular endothelial cells, inducing autophagy while stabilizing Nrf2 to increase antioxidant defenses. Conversely, Nrf2 inhibition blocks antioxidant gene activation, resulting in ROS accumulation, macromolecular damage, and cell death.^484^ This approach shows potential for use in ROS-mediated cancer therapy, warranting further exploration. Immune therapy-PI combination might enhance PI sensitivity, as evidenced in multiple myeloma (MM), where IL-33 enhances Bortezomib sensitivity by inducing excessive ROS.^485^ The IL-33-Bortezomib combination suppressed MM cell proliferation by boosting ROS levels, inhibiting NF-κB-p65 nuclear translocation, and downregulating the expression of stemness genes—effects reversed by the addition of ROS scavengers.

##### NQO1 inhibition in combination therapy

Coinhibition of GSTP1 and NQO1 shows antitumor potential in GBM, where EGFR mutations upregulate these enzymes to suppress ROS and drive tumor growth. The small-molecule inhibitor MNPC potently targets both NQO1 and GSTP1, disrupting redox homeostasis, elevating ROS, and inducing apoptosis in EGFRvIII-mutant GBM cells.^486^ Additionally, therapy-resistant CSCs, including those in TNBC, rely on DPP9-upregulated Nrf2-mediated antioxidant defenses (NQO1, SOD1) to suppress ROS and survive. Targeting these vulnerabilities, the NQO1-bioactivatable agent IB-DNQ generates ROS to kill CSCs, with its efficacy enhanced by concurrent SOD1 inhibition, which is elevated in TNBC.^487^

NQO1 inhibition may help overcome therapeutic resistance, as demonstrated in several preclinical and clinical trials. A study revealed that DPP9 promotes chemoresistance in liver cancer by binding to KEAP1, stabilizing Nrf2, and upregulating NQO1 expression to lower ROS and reduce chemotherapy efficacy.^107^ Inhibiting NQO1 with Dicoumarol restores ROS accumulation and enhances the effectiveness of chemotherapy in liver cancer. ARQ761 is under clinical evaluation with Gemcitabine and Nab-paclitaxel (NCT02514031) for treating NQO1-overexpressing tumors. A study enhanced CDT by using nanovesicles (NV-IONP-Lapa) loaded with iron oxide nanoparticles (IONPs) and β-Lapachone (Lapa). NQO1 catalyzed Lapa to generate H₂O₂, while IONPs released iron ions in the acidic tumor environment, converting H₂O₂ into cytotoxic OH• via the Fenton reaction. This system showed excellent tumor-targeting potential with minimal side effects, offering a promising approach for treating NQO1-overexpressing tumors.^488^

Targeting DDR and NQO1 may synergistically enhance antitumor efficacy while minimizing β-Lapachone-associated toxicity. Combining β-Lapachone with proliferating cell nuclear antigen (PCNA) inhibitor T2AA blocks DNA repair complex assembly, exacerbates ROS-induced DSBs, and enhances antitumor efficacy, as shown in Lewis lung carcinoma models.^489^ Similarly, combining the PARP inhibitor Rucaparib with β-Lapachone amplifies ROS generation and shifts the cell death mechanism from necrosis to caspase-dependent apoptosis, improving tumor-selective killing in NQO1-positive xenograft models.^490^ A clinical trial (NCT03575078) evaluating ARQ761 with Olaparib was initiated but withdrawn due to lack of patient enrollment.

##### Nrf2 inhibition in combination therapy

Nrf2 inhibition enhances antitumor therapy in various cancers by overcoming MDR linked to elevated ROS and Nrf2 levels. In a study on lung cancer, targeting the ROS/Nrf2 axis with Nrf2 siRNA or Tangeretin was shown to suppress Nrf2, overcome MDR (downregulating P-gp), and synergize with Paclitaxel and AZD9291 in A549/T xenografts.^491^ Similarly, to overcome hypoxia- and hyperthermia-induced phototherapy resistance, a MnO₂- and Ce6/PDA-PEG-FA**-**decorated silica nanonetwork loaded with Brusatol was engineered. MnO₂ generated oxygen to amplify Ce6-mediated PDT and PDA-driven PTT while targeting HSPs, and Brusatol inhibited the Nrf2‒GPX4 axis, inducing ferroptosis and phototherapeutic efficacy.^492^

##### Targeting DDR in combination therapy

Combining PARP inhibitors (PARPis) with ferroptosis inducers (FINs) may overcome PARPi resistance in BRCA-wild-type ovarian cancer. A study revealed that PARP inhibition induces ferroptosis via p53-mediated SLC7A11 downregulation, depleting glutathione and increasing ROS-driven lipid peroxidation in BRCA-mutant ovarian cancer. Ferroptosis suppression contributes to resistance in BRCA-wild-type cancers, while PARPi-FIN combinations synergistically enhance cytotoxicity in BRCA-proficient ovarian cancer models both in vitro and in vivo.^493^ Additionally, to address soft tissue sarcoma resistance to PDT, ZTN@COF@poloxamer nanocapsules were developed, integrating a Zr-based MOF, the photosensitizer Tetrakis (4-carbethoxyphenyl) porphyrin, and the PARP inhibitor Niraparib, where light-activated ROS generation induces apoptosis, enhancing PDT efficacy and blocking DNA repair.^494^

In addition, several other combination therapies targeting ROS promotion have been studied to overcome resistance to conventional anticancer agents. For example, recurrence in nasopharyngeal carcinoma often arises from radioresistance. A study showed that SOD2 knockdown amplifies radiation-induced superoxide production and lipid peroxidation, enhancing radiosensitivity via ferroptosis.^495^ However, DHODH inhibition in SOD2-depleted cells reduced oxidative stress, lipid peroxidation, and ferroptosis, improving survival and colony formation, suggesting that DHODH activity sustains radiation-induced ferroptosis. Therefore, caution is needed when combining DHODH inhibitors with radiotherapy, as they may counteract radiosensitization. NOX activator, Berbamine Hydrochloride has also been found to synergize with Cisplatin in lung carcinoma cells by inducing excessive ROS, impairing lysosomal acidification via NOX2 recruitment, triggering MAPK-mediated apoptosis, and increasing cell death.^496^ Photochemotherapy might be a promising strategy for precision nanotheranostics, as demonstrated by IMTD, a nanotheranostic coassembling ICG photosensitizer with Mannose (MAN)- Thioketal linker (TK)- Doxorubicin (DOX), for tumor-targeted, light/ROS-responsive photochemotherapy. IMTD prevents premature DOX leakage, enhances tumor accumulation via mannose receptor uptake, and enables spatiotemporal drug release through laser-triggered ROS generation and thioketal cleavage in vitro/in vivo.^497^

Tubeimoside-I, a heat shock protein inhibitor, has been reported to synergize with Oxaliplatin in CRC by increasing ROS levels, downregulating HSPD1, and upregulating ER stress and MAPK pathways, thereby enhancing the anticancer effects of Oxaliplatin.^498^ To overcome hypoxia and ROS limitations in tumor PDT, the cell membrane-targeting photosensitizer TBzT-CNQi was developed to generate ¹O_2_, OH•, and O_2_•⁻, inducing lipid peroxidation and enhancing ferroptosis.^499^ Moreover, combining TBzT-CNQi with ferroptosis suppressor protein 1 inhibitor (iFSP1) downregulated FSP1, reduced CoQ10, and amplified lipid peroxidation, triggering immunogenic ferroptosis. This combination activated immune responses, increased CD8^+^ T cells, and reduced tumor-associated macrophages, offering a promising strategy for photodynamic immunotherapy in hypoxic tumors. Immunotherapy faces challenges from poor tumor immunogenicity and immunosuppressive microenvironments. ICD inducers like Paclitaxel (PTX) stimulate immunity via DAMP release but are limited by poor targeting and immunosuppressive effects. Building on synergistic ICD inducers and IDO inhibitors, a study developed ROS-responsive PTX@PoxMTP NPs to codeliver PTX and 1-MT (IDO inhibitor).^500^ Tumor-specific ROS trigger PTX release, inducing ICD and amplifying ROS, whereas esterase-activated 1-MT disrupts the IDO1/kynurenine pathway to reduce immunosuppression. This synergy enhances dendritic cell maturation and T-cell infiltration, and suppresses Tregs and TAMs, resulting in robust tumor regression and metastasis control with minimal off-target effects.

#### Combination therapeutic strategies targeting ROS suppression

Several studies have investigated combination strategies to overcome resistance and improve tumor-specific responses by suppressing ROS levels. A novel regimen combining Sodium arsenite (NaAsO₂), Chloroquine (CQ), and Dichloroacetate (DCA) targets chemoresistance in oral squamous cell carcinoma (OSCC) by regulating ROS, autophagy, and glycolysis. NaAsO₂ induces cytotoxic ROS and inhibits glycolysis; DCA enhances this effect, while CQ blocks autophagy. This combination boosts antioxidant enzymes, restores redox balance, improves survival (90% vs. 35%), and reduces tumor progression in OSCC mouse models with minimal toxicity, offering a promising strategy against chemoresistance.^501^

The efficacy of immune checkpoint blockade (ICB) is often limited by MDSC-mediated immunosuppression. Tumor-derived GM-CSF activates STAT3, upregulating Fatty Acid Transport Protein 2 (FATP2) to drive lipid accumulation and ROS production, critical for MDSC function. A study demonstrated that FATP2 inhibition with Lipofermata disrupted lipid metabolism, reduced ROS, and blocked MDSC suppression, enhancing antitumor responses.^502^ This approach has synergized with anti-PD-L1 therapy by boosting CD8^+^ T-cell activity and restoring antitumor immunity, offering a metabolic reprogramming strategy to improve ICB efficacy.

In addition, many preclinical and clinical studies have been conducted and are ongoing to explore the antiproliferative, anti-invasive, antiangiogenic, and chemoresistance-modulating properties of antioxidants like Curcumin, Lycopene, and Vitamin C. A clinical trial (NCT03072992) revealed that intravenous Curcumin (CUC-1®) combined with Paclitaxel significantly improved the ORR (51%) compared to placebo (33%), with an even greater effect in patients completing treatment.^503^ Lycopene alone had no significant effect, but coadministration with Docetaxel showed low toxicity, favorable pharmacokinetics, and improved angiogenesis and IGF-1 signaling in metastatic prostate carcinoma (NCT0149519).^504^

### Personalized cancer treatment utilizing the ROS signature

ROS-related biomarkers are essential for personalized medicine because of the observed heterogeneity in redox responses across different cancer types, stages, and individuals with the same cancer. Protein carbonyls, arising from oxidative cleavage, deamination, and Michael addition, along with advanced glycation end products (AGEs) like carboxymethyl lysine (CML), are among these markers.^505,506^ Additionally, lipid peroxidation products such as acrolein, malondialdehyde (MDA), 4-hydroxy-2-nonenal (4-HNE), and F2-isoprostanes (IsoPs) are also indicative of ROS-related damage.^506,507^ Nucleic acid oxidation products like 8-oxo-dG (8-oxo-2’-deoxyguanosine) are commonly used biomarker for oxidative stress and DNA damage caused by ROS.^508^ Elevated 8-oxo-dG indicates oxidative damage, and to date, many clinical studies have utilized plasma levels of 8-oxo-dG to monitor oxidative stress and DNA damage in cancer.

The thiol‒disulfide balance, representing the equilibrium between reduced thiols (-SH groups) and oxidized disulfides (-S‒S‒ bonds) in cells, serves as a redox biomarker.^509^ In a study of 62 cervical cancer patients and 61 healthy women (NCT04258553), Sezgin et al. reported a significant positive correlation between disulfide levels and cervical cancer stage, proposing thiol-disulfide balance as a potential early biomarker for this disease.^510^ Antioxidant enzymes are key biomarkers of oxidative stress and are extensively studied as ROS-related indicators in numerous cancer clinical trials.^11^ A study in ovarian cancer (NCT03470857) reported that elevated SOD activity might be linked to increased ROS. Another study (NCT01985113) reported that plasma TRX levels are significantly regulated in lung cancer patients (>7.1 U/mL), indicating its diagnostic potential.

Promoter methylation sites such as 5-methylcytosine (5-mC), RUNX3, DNMT1, and p16 are the key gene targets for hypermethylation induced by ROS, and their epigenetic alterations can serve as useful biomarkers for cancer diagnosis.^511^ Noncoding RNAs (ncRNAs), including microRNAs (miRNAs) and long noncoding RNAs (lncRNAs), play critical roles in regulating ROS-related pathogenesis, making them promising biomarkers and therapeutic targets in cancer.^512^ For example, miR-373 functions as a redox regulator and promotes tumorigenesis in various cancers, while miR-371 is linked to cancer metastasis, drug resistance, and recurrent germ cell cancers (NCT04435756).^513^ Additionally, miR-155, linked to oxidative stress and tumorigenesis via the Nrf2 pathway, is targeted by Cobomarsen, a Phase II trial of a miR-155 inhibitor (NCT03713320) for cutaneous T-cell lymphoma (CTL) and mycosis fungoides.^514^ HOTAIR, a lncRNA, regulates chromatin structure and gene expression, promoting tumor progression and metastasis in breast, colorectal, and lung cancer.^515^ Mitochondrial lncRNAs like ASncmtRNA, a redox sensor with oncogenic roles, are targeted by Andes-1537, an antisense oligonucleotide showing promise in Phase I trials (NCT02508441, NCT03985072) for solid tumors.^516^ Conversely, miR-16 suppresses tumor growth by regulating oxidative stress and promoting apoptosis. In a Phase I trial (NCT02369198), an EGFR-targeted miR-16 mimic showed promise in treating mesothelioma and NSCLC.^517^ Another tumor suppressor involved in redox regulation, miR-34a, has been extensively tested in animal models. MRX34, a miR-34a mimic, was developed to assess its safety, pharmacokinetics, and pharmacodynamics in a Phase I trial (NCT01829971). Besides, transcription factors like Nrf2 and p53 act as crucial ROS-related biomarkers in cancer diagnosis and treatment, balancing between protecting cells from oxidative stress and contributing to tumor growth under certain conditions.

Some ROS-related biomarkers that are clinically used to assess the redox state of the body or specific tissues and cells in cancer are listed in Table 6. The data concerning the oxidative biomarkers have been collected from www.fda.gov, http://clinicaltrials.gov, http://go.drugbank.com, and different works of literature.

**Table 6:** List of ROS-related biomarkers and their clinical implications in cancer

Type of markers	Name of markers	Disease/condition	Clinical trials		
			Phase	ID	Status
Protein carbonyls and advanced glycation end products	Carboxymethyl Lysine (CML)	Prostate cancer	I/II	NCT03712371	Terminated
		Breast cancer	NA	NCT04716764	Unknown
		Breast cancer	NA	NCT05265715	Completed
Lipid peroxidation products	F2-isoprostanes (F2-IsoPs)	Lung cancer	II	NCT02719860	Completed
		Pancreatic cancer	II	NCT01515046	Terminated
		Prostate cancer	I	NCT00895115	Completed
		Breast cancer	II/III	NCT00393172	Completed
		Colon cancer	NA	NCT01470586	Completed
	Trans-4-hydroxy-2-nonenal (4-HNE)	Liver cancer	II	NCT00513461	Completed
		Anal cancer	I/II	NCT03386500	Recruiting
		High-grade glioma	Early I	NCT04559685	Recruiting
		Pediatric cancer	NA	NCT05255445	Completed
	Malondialdehyde (MDA)	Neoplasms	NA	NCT06247865	Completed
		Oral cancer	NA	NCT04267419	Completed
		Prostate cancer	IV	NCT02149628	Completed
		Breast cancer	MA	NCT00539422	Completed
		Polycystic ovary syndrome	NA	NCT02954120	Completed
		Liver cancer	II/III	NCT01964001	Completed
		Acute myeloid leukemia	I/II	NCT05580861	Recruiting
		NSCLC	NA	NCT04676009	Completed
		Gastrointestinal carcinoma	NA	NCT02871999	Completed
		Laryngeal cancer	NA	NCT05857202	Unknown
		Colon cancer	NA	NCT01596634	Completed
	Acrolein	Smoking-related carcinoma	II	NCT05121051	Recruiting
		Lung cancer	NA	NCT03402230	Completed
Nucleic acid oxidation products	8-oxo-2’-deoxyguanosine (8-oxo-dG)	Colon cancer	NA	NCT03550885	Active, not recruiting
		Premalignant lesion	NA	NCT03830710	Completed
		Esophageal carcinoma	II	NCT01097304	Completed
		Lung cancer	II	NCT02719860	Completed
		Bladder cancer	NA	NCT03286699	Terminated
		Oral cancer	NA	NCT04372914	Completed
		Breast cancer	NA	NCT02938780	Completed
		Prostate cancer	II	NCT03824652	Recruiting
Thiols	Thiol-disulfide balance	Cervix cancer	NA	NCT04258553	Completed
		Endometrium cancer	NA	NCT04175067	Completed
		Ovarian cancer	NA	NCT05011539	Completed
		Breast cancer	NA	NCT04479098	Completed
Antioxidant enzymes	Superoxide dismutase (SOD)	Head and neck cancer	NA	NCT01771991	Completed
		Neoplasm	NA	NCT03470857	Completed
		Liver cancer	II/III	NCT01964001	Completed
		Prostate cancer	NA	NCT00898274	Completed
	Catalase (CAT)	Polycystic ovarian syndrome	NA	NCT03792282	Completed
		Malignant neoplasm	Early I	NCT05985278	Recruiting
		Breast cancer	NA	NCT04354233	Recruiting
	Glutathione peroxidase (GPX)	Hepatocellular carcinoma	NA	NCT04601961	Unknown
		Prostate cancer	NA	NCT01917890	Completed
		Head and neck cancer	NA	NCT05451576	Recruiting
	Glutathione reductase (GR)	Breast cancer	NA	NCT03516253	Unknown
		Non-Hodgkin’s lymphoma	NA	NCT03260231	Unknown
	Glutathione S-transferases (GSTs)	Cancer	NA	NCT01293591	Completed
		NSCLC	NA	NCT06140082	Not yet recruiting
		Polycystic ovarian syndrome	IV	NCT02027337	Unknown
	Thioredoxin reductase	Lung cancer	NA	NCT01985113	Completed
			I	NCT02166242	Completed
				NCT01980212	Completed
		Pancreatic cancer	II	NCT00417287	Terminated
		Rectal cancer	NA	NCT00972751	Completed
Promoter methylation site	5-methylcytosine	AML, MDS, CMML	NA	NCT03526666	Completed
		Colorectal cancer	NA	NCT04196803	Completed
		Lung adenocarcinoma	NA	NCT06477211	Completed
	RUNX3	Gastric adenocarcinoma	II	NCT00674167	Unknown
		Lung cancer	II/III	NCT02416739	Unknown
		Colorectal cancer	NA	NCT05638542	Unknown
	DNMT1	Gastric carcinoma	NA	NCT05811546	Unknown
		Glioma	NA	NCT04097535	Completed
			II	NCT04765514	Recruiting
			NA	NCT02997423	Completed
	p16	Lung cancer	NA	NCT01038492	Completed
		Barrett’s Esophagus	III	NCT01566474	Completed
		Cervical cancer	NA	NCT05049252	Unknown
		Ewing sarcoma	NA	NCT00898053	Completed
		Breast cancer	NA	NCT01138345	Completed
		Oral epithelial dysplasia	NA	NCT00835341	Completed
		Non-Hodgkin’s lymphoma	NA	NCT00026910	Completed
Non-coding RNAs (ncRNA)	miR-373	Breast cancer	NA	NCT04720508	Unknown
	miR-155	Bladder cancer	NA	NCT03591367	Completed
	miR-371	Germ cell cancer	NA	NCT04435756	Active, not recruiting
	HOTAIR	Thyroid cancer	NA	NCT03469544	Unknown
	miR-16	Pleural mesothelioma lung cancer	I	NCT02369198	Completed
	miR-122	Liver cancer	I/II	NCT01646489	Completed
			I/II	NCT01727934	Unknown
			I/II	NCT01872936	Unknown
			I/II	NCT01200420	Completed
	miR-155	T-cell lymphoma	II	NCT03713320	Terminated
	miR-34a	Melanoma	I	NCT01829971	Terminated
			I	NCT02862145	Withdrawn
	mtlncRNA	Unresectable tumors	I	NCT02508441	Terminated
			I	NCT03985072	Completed
Transcription factors	Nrf2	Head and neck cancer	Early I	NCT03182959	Completed
		Smoking-related carcinoma	Early I	NCT03402230	Completed
	p53	Acute myeloid lymphoma	I	NCT03855371	Unknown
		Esophageal cancer	III	NCT00525200	Completed

## Conclusion and perspective

ROS play a dual role in mammalian cells and are essential yet potentially toxic to both normal and cancerous tissues, making selective targeting through ROS-modulating therapies inherently challenging. The “Threshold concept for cancer therapy” suggests that tumor cells, with higher inherent ROS levels, can be selectively targeted through pro-oxidant therapy, exploiting their impaired redox homeostasis.^518^ However, the lack of comparable normal cell controls highlights the need for larger paired studies and in vivo investigations to validate its translational potential.

Developing antioxidant-based therapies poses significant challenges due to the antioxidant paradox. While antioxidants protect healthy cells by mitigating oxidative stress, cancer cells exploit elevated antioxidant levels for survival.^14^ Nonspecificity presents another great challenge in developing this therapeutic regime. For example, NOX inhibitors like DPI and VAS3947 affect pathways beyond NOX.^519^ DPI disrupts mitochondrial respiration, NOS, and other complexes, causing off-target superoxide bursts, while VAS3947 induces apoptosis via cysteine thiol alkylation, independent of NOX activity. These off-target effects can lead to misleading and unexpected outcomes in studies of NOX-related oxidative metabolism in cancer. Moreover, antioxidants like Ascorbate, Dimethyl Fumarate (DMF), and Lycopene can act as pro-oxidants or antioxidants depending on the cellular context. Given these challenges, antioxidant-based therapies may be better suited for cancer prevention, as cancer cells can exploit antioxidants for survival. Research should prioritize understanding their context-dependent pro-oxidant and antioxidant effects to improve selectivity. TME-based studies could increase treatment efficacy, addressing nonspecificity and resistance. For example, NOX4 promotes tumor growth and metastasis under hypoxic conditions, making selective NOX4 inhibitors potential treatments for hypoxia-inducible cancers.^520^ Furthermore, in the absence of specific agents, genetic approaches like gene knockdown offer precision therapy.

Targeting ROS promotion faces challenges due to metabolic plasticity and off-target toxicity. While polypharmacology enhances versatility, achieving tumor selectivity remains difficult. The efficacy of ROS-inducing agents depends on tumor-specific genetics and metabolism. For example, RSL3, a GPX4 inhibitor, induces ferroptosis in pancreatic cancer but varies in effectiveness based on the KRAS mutations and metabolic adaptations.^521^ Such observations underscore the necessity of developing context-specific therapies, which require the consideration of oncogenic drivers, metabolic dependencies, and redox regulation. ROS like H₂O₂ can amplify cancer cell death via bystander effects but may also harm normal cells, necessitating tissue- and systemic-level impact assessment.

Targeting ROS-dependent pathways for drug development is challenging because of the dual role, complex regulation, and context-dependent activity of ROS. Key considerations include several critical questions: Is the molecule’s regulation by ROS conserved, and what is its mode of regulation? How does it influence specific pathways, and what is the interplay between ROS and those pathways, including potential interference from other ROS-regulated molecules? Furthermore, does the target molecule affect a single pathway or multiple pathways? The tumor microenvironment (TME) adds another layer of complexity, as shown in cervical cancer cells: MEK inhibition (MEKi) modulates ROS differently across cell lines, with ERK reducing ROS in C33-A cells but increasing it in SiHa and CaSki cells.^522^ Combined ROS and ERK inhibition showed synergistic effects on CaSki and HeLa cells but not on C33-A or SiHa cells, underscoring the context-dependent ROS-ERK interplay. These complexities highlight the importance of precisely understanding ROS–target interactions and pathway dynamics to effectively leverage ROS-dependent mechanisms while minimizing unintended effects.

Combination therapeutic strategies in cancer focus on ROS induction rather than suppression, primarily owing to the complex and context-dependent role of ROS in malignancy, as exemplified by the “Antioxidant Paradox”. ROS-inducing therapies, combined with chemotherapy or radiotherapy, offer promise by amplifying oxidative stress selectively. However, challenges like bioavailability, off-target effects, TME heterogeneity, and metabolic plasticity persist.^523^ Nanoparticle delivery systems enable precise, TME-responsive drug release by exploiting high levels of ROS, altered pH, or elevated NQO1 levels in tumors. Hypoxia further complicates ROS-based therapies, but oxygen-independent strategies—such as Fenton-like metal catalysts (Fe/Cu), hypoxia-activated prodrugs (HAPs), SDT, and NQO1 bioactivatable drugs (e.g., β-Lapachone)—show particular promise.^524^ Furthermore, a major hurdle in ROS-targeted cancer therapy is the translation of mechanistic insights into clinically viable drugs. To address this, combining gene-editing tools (e.g., CRISPR or RNAi) with conventional therapies offers a promising solution—enabling precise disruption of redox-regulating genes (e.g., NOX4 and HIF1α) while leveraging the broad cytotoxicity of chemotherapy.

With respect to ROS-related biomarkers for personalized treatment, measuring specific ROS biomarkers like F2-isoprostanes or 8-oxo-dG, along with oxidative damage products, enhances sensitivity but faces challenges due to the transient and fluctuating nature of ROS.^11^ Stable isotopes and advanced techniques such as LC‒MS or UPLC‒MS/MS might improve detection accuracy, whereas real-time monitoring tools, such as fluorescence spectroscopy and electrochemical sensors, can capture dynamic ROS activity.^525^ Tissue-specific analyses and redox modulators provide deeper insights into organ- or cell-specific ROS behavior. Recent advancements, including deep tissue fluorescence imaging, metabolomics, and mass spectrometry imaging, have enabled in vivo investigations of redox mechanisms in tumors,^526^ but further research is needed to achieve full precision and efficacy. Ultimately, unraveling how redox modulation drives tumor initiation, progression, and resistance will guide the rational design of context specific, clinically relevant therapies that exploit tumor-specific redox vulnerabilities while minimizing harm to normal tissues.

## Data Availability

All data used and/or analyzed in this review are available within the article and the referenced sources.
